# Parasites of Three Closely Related Antarctic Fish Species (Teleostei: Nototheniinae) from Elephant Island

**DOI:** 10.1007/s11686-021-00455-8

**Published:** 2021-07-17

**Authors:** Katharina G. Alt, Sarah Cunze, Judith Kochmann, Sven Klimpel

**Affiliations:** 1 Institute for Ecology, Evolution and Diversity, Goethe University, 60438 Frankfurt am Main, Germany; 2 Senckenberg Biodiversity and Climate Research Centre, Senckenberg Gesellschaft für Naturforschung, 60325 Frankfurt am Main, Germany

**Keywords:** Demersal fish, Marine food webs, Antarctic parasites, Southern Ocean

## Abstract

**Background:**

Studies of parasite communities and patterns in the Antarctic are an important knowledge base with the potential to track shifts in ecological relations and study the effects of climate change on host–parasite systems. Endemic Nototheniinae is the dominant fish group found in Antarctic marine habitats. Through their intermediate position within the food web, Nototheniinae link lower to higher trophic levels and thereby also form an important component of parasite life cycles. The study was set out to gain insight into the parasite fauna of *Nototheniops larseni*, *N. nudifrons* and *Lepidonotothen squamifrons* (Nototheniinae) from Elephant Island (Antarctica).

**Methods:**

Sampling was conducted at three locations around Elephant Island during the ANT-XXVIII/4 expedition of the research vessel Polarstern. The parasite fauna of three Nototheniine species was analysed, and findings were compared to previous parasitological and ecological research collated from a literature review.

**Results:**

All host species shared the parasites *Neolebouria antarctica* (Digenea), *Corynosoma bullosum* (Acanthocephala) and *Pseudoterranova decipiens* E (Nematoda). Other parasite taxa were exclusive to one host species in this study. *Nototheniops nudifrons* was infected by *Ascarophis nototheniae* (Nematoda), occasional infections of *N. larseni* with *Echinorhynchus petrotschenkoi* (Acanthocephala) and *L. squamifrons* with *Elytrophalloides oatesi* (Digenea) and larval tetraphyllidean Cestoda were detected.

**Conclusion:**

All examined fish species’ parasites were predominantly euryxenous regarding their fish hosts. The infection of *Lepidonotothen squamifrons* with *Lepidapedon garrardi* (Digenea) and *Nototheniops larseni* with *Echinorhynchus petrotschenkoi* represent new host records. Despite the challenges and limited opportunities for fishing in remote areas, future studies should continue sampling on a more regular basis and include a larger number of fish species and sampling sites within different habitats.

**Supplementary Information:**

The online version contains supplementary material available at 10.1007/s11686-021-00455-8.

## Introduction

The Antarctic Circumpolar Current (ACC) which forms the boundaries of the Southern Ocean is the biggest physio-thermal barrier found in the world oceans [1, 2]. With its strong eastward current and thermohaline frontal systems it serves as a natural boundary for most organisms inhabiting the Southern Ocean which promoted a high degree of endemism and adaptation to the distinctive features of this cold environment [3].

The Antarctic Peninsula (AP) extends from the Antarctic continent towards the southern extension of South America (Cape Horn, Chile), and is connected by the Drake Passage and the Polar Front found between 56.8° S and 59.3° S [4]. The area around Elephant Island (South Shetland Islands) is characterised by its high net production as one of the nursery areas of the keystone species Antarctic krill (*Euphausia superba* Dana, 1850), the region’s most important energy resource [5–9]. It belongs to the seasonal pack ice zone, which is defined by not being covered by an ice sheet perennially. As a result, the region has a different habitat structure compared to high Antarctic areas (from 70° South), such as a steep shelf slope, lack of littoral, vast depth range, and unique marine fauna [10]. Average temperatures at the western AP have increased during the last years of proceeding climate change and reached the maximum measured temperature of 18.4 °C, February 6th 2020, measured at Esperanza research base [11, 12] (World Meteorological Organization 2020). The resulting decrease in sea ice is expected to severely impact the local ecosystem [5]. Since krill depends on ice algae as their winter food, the decrease of ice could lead to a shift in the communities of pelagic feeders and all dependent organisms [5].

Inshore fish are an essential link for the energy flow between invertebrates and higher predators. They are mostly consumers of benthos and zooplankton and prey of predatory mammals and birds, which promotes energy transport from sea to land. In offshore regions, fish forage on nekton and zooplankton and are preyed on by larger fish, because they are out of reach for most seals and birds [13]. Despite krill being the most important factor for energy flow in offshore habitats of the Southern Ocean, demersal fish seem to be more important in inshore habitats than krill [13].

The Notothenioidea are the most dominant fish group found in the Southern Ocean and the paragon of the adaptive radiation of teleosts in the marine environment [14]. The Nototheniidae have evolved from strictly benthic ancestors characterised by the lack of a swim bladder. Important features shared by all Nototheniidae are slow ontogenesis and long generation time [2, 10, 15]. Throughout their radiation and diversification, some species have evolved to follow a benthopelagic lifestyle. The 16 nototheniid species occurring outside the Southern Ocean [16] are all adapted to a benthic habitat and possess a different parasite fauna than Antarctic Nototheniidae [16–20]. The subfamily Nototheniinae exclusively occurs in the Southern Ocean [17]. *Nototheniops larseni* (Lönnberg, 1905), *N. nudifrons* (Lönnberg, 1905) and *Lepidonotothen squamifrons* (Günther, 1880; syn. *L. kempi*) are among the most abundant fish species of the West Antarctic Peninsula and Southern Scotia Arc [13, 21, 22]. They are distinguished by their depth ranges and habitats which results in *Nototheniops nudifrons* being more abundant inshore (e.g. fjords), *N. larseni* (also occurs inshore) and *L. squamifrons* (exclusively found at the outer shelf) being more common in offshore areas [13]. With their intermediate position within the food web, these species create an important link for energy flow from lower to higher trophic levels and play an important part in the life cycles of parasites.

The diversity and abundance of parasite infection in a host are, among other factors (i.e. host age/size), connected to the variability and trophic level of its diet. Infection rates of zooplankton and macroinvertebrates with helminth parasite stages are lower than the parasite prevalence in small fish [23–25]. By including higher trophic level organisms into its diet, a host is more likely to acquire parasites from its food. But parasites can also be used as an indicator of diet components of their hosts in addition to stomach content analysis, which can only represent the diet at the time of sampling. If the parasites’ life cycles are known, infection patterns can provide information about the higher trophic levels and potential predators of their hosts.

The fundamental research in the field of Antarctic parasitology (notably K. Zdizitowiecki and colleagues) has provided a knowledge base to track the shift of ecological relations in an ecosystem eminently affected by increasing temperatures. However, early studies have mostly focused on parasite species descriptions, identification keys, and life cycles of certain taxa [26–29], while studies on the parasite communities of the fishes examined [30, 31] are more scarce. In this study, we aim to contribute to the monitoring of marine communities from the seasonal pack ice zone off Elephant Island. Based on parasitological data, collected during an expedition of the RV Polarstern, and a literature review we intend to gain insight into the ecology of three fish species of the subfamily Nototheniinae, *Nototheniops larseni*, *N. nudifrons* and *Lepidonotothen squamifrons*, more specifically their diet, potential predators and their position in the food web.

## Materials and Methods

### Sampling

**Fig. 1:**
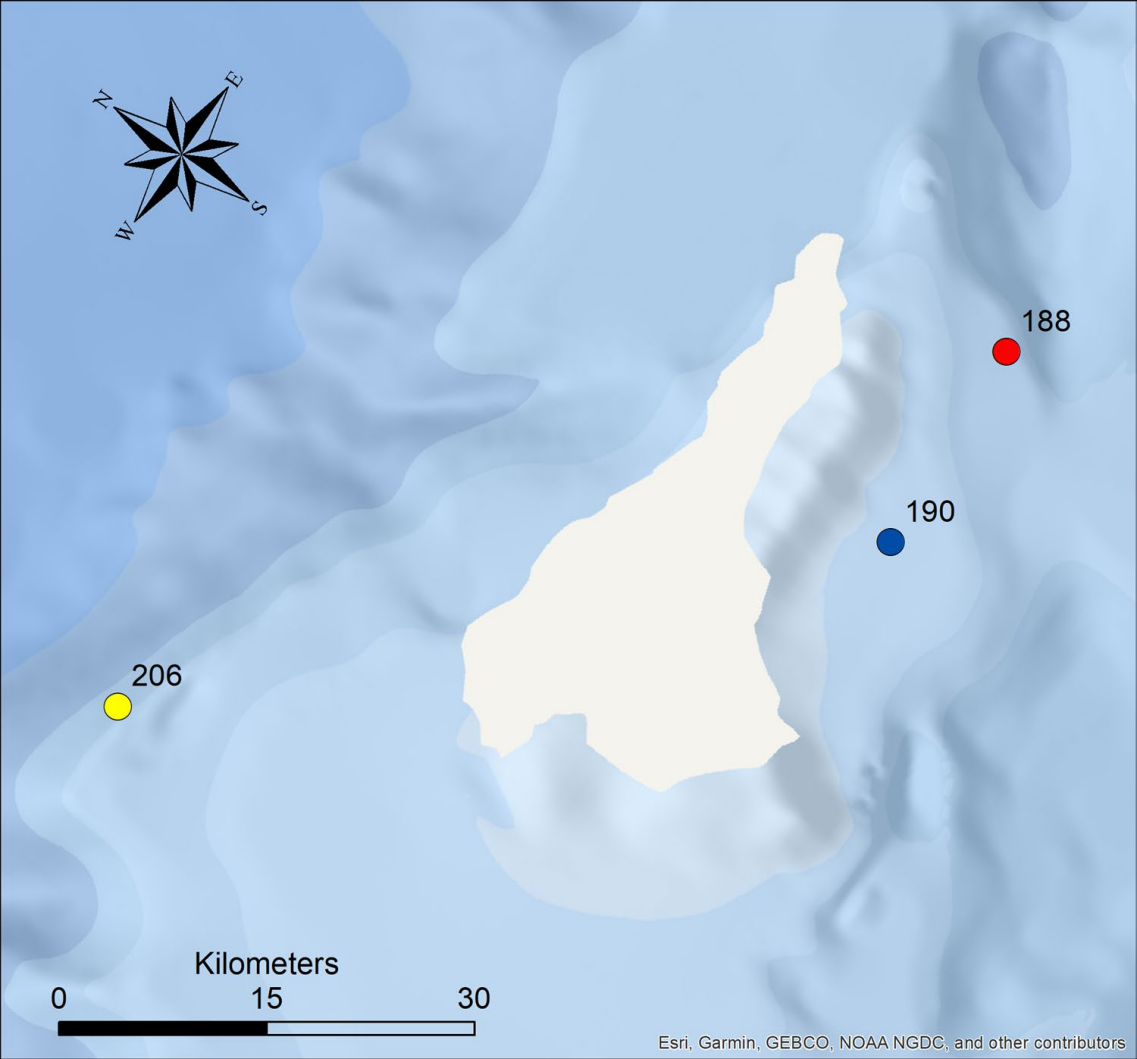
Sampling locations of bottom trawl fisheries around Elephant Island. Haul 188 = red, haul 190 = blue, haul 206 = yellow. (Projection: South Pole Lambert Azimuthal Equal Area, WKID: Authority: 102020 (Esri), Geographic Coordinate System: GCS_WGS_1984.) (colour figure online)

Host sampling of Nototheniidae, *Nototheniops larseni* (*n* = 40), *N. nudifrons* (*n* = 40) and *Lepidonotothen squamifrons* (*n* = 49), was conducted at the ANT-XXVIII/4 expedition of the RV Polarstern to the Antarctic Peninsula (CCAMLR Subarea 48.1), from March 13th to April 9th, 2012. Specific sampling locations were situated around Elephant Island (Fig. 1, Table S1). Fishing was performed through bottom trawling. The catch was sorted, identified, measured, weighted and fish samples were stored at − 20 °C until further examination.

### Host and Parasite Taxonomy

Despite contradictory database entries about the taxonomic status of the fishes examined in this study (the online platforms FishBase [32] and World Register of Marine Species [33] assign the genus *Lindbergichthys*) this study follows Eschmeyer’s Catalog of Fishes [34] referring to the taxonomy proposed by Near *et al.* [35], assigning the genus *Nototheniops* to both *N. larseni* and *N. nudifrons*. The taxonomy of *Lepidonotothen squamifrons* follows the findings of Miya *et al.* [36]. All references agree on the placement of *L. squamifrons, N. larseni* and *N. nudifrons* into the subfamily Nototheniinae.

Since the parasitological findings from this study need to be put into the context of previous research, a list of the recorded parasites of *Nototheniops larseni*, *N. nudifrons* and *Lepidonotothen squamifrons*, was compiled. This was based on the checklist of Antarctic fish parasites by Oğuz *et al.* [37] complemented by the entries in the host–parasite database of the Natural History Museum London [38] and search on Google Scholar. The changes of host taxonomy were taken into account by including the known synonyms: *Notothenia larseni, Lepidonotothen larseni* for *Nototheniops larseni; Notothenia nudifrons, Lepidonotothen nudifrons, Lindbergichthtys nudifrons, Notothenia mizops nudifrons* for *Nototheniops nudifrons* and *Notothenia squamifrons, Lepidonotothen kempi, Notothenia brevipectoralis, Notothenia kempi, Notothenia macrophthalma* for *Lepidonotothen squamifrons*. Outdated systematics of parasite records were revised to recent taxonomic classification using the World Register of Marine Species [33]. Despite their careful assembly, the lists might not be fully exhaustive. The findings were visualised as bipartite networks using R version 4.0.3, following a script by Brandl *et al.* [39].

### Host Morphometry and Diet

The fish samples were thawed at room temperature and morphometric measures were taken. The diet of the fishes was examined by analysing the stomach contents. Full and empty stomach weight was determined, and each food item was identified to the lowest possible taxonomic level. The different food organisms were counted and weighted. Trophic measures were calculated according to Hyslop [40].

### Parasitological Examination

The host fishes were examined for metazoan ecto- and endoparasites, using a stereomicroscope (Olympus SZ61) with transmitting light (Olympus KL1600 LED, Olympus Corporation). The body surface and buccal and nasal cavities were examined for ectoparasites, and the visceral cavity, organs, and alimentary tract were examined for endoparasites. Parasites were washed in saline solution or purified water (Acanthocephala), determined taxonomically, and counted. Parasitological parameters were calculated as stated in Bush *et al.* [41].

Parasite species were identified either by their morphological (Digenea, Cestoda and Acanthocephala) or molecular characteristics (Nematoda). For morphological identification, specimens were treated with 4% Roti-Histofix (Roth) and mounted on microscope slides in glycerine. The respective keys by Zdzitowiecki [42, 43] were used. Pictures were taken with an Olympus BX53 microscope and cellSens Standard software version 1.14 (Olympus Corporation). Specimens of the identified parasites were deposited in the scientific collection of the Senckenberg Research Institute and Natural History Museum, Frankfurt am Main, Germany, Catalogue Number SMF 15198 (*Elytrophalloides oatesi*), 15,199 (*Lepidapedon garrardi*), 15200 (*Neolebouria antarctica*), 17065 (*Corynosoma bullosum*) and 17066 (*Echinorhynchus petrotschenkoi*).

Nematodes were identified genetically because larval stages often lack distinct morphological features. Due to the high number of Nematoda infecting each host species, a subsample was taken to reduce sequencing costs. A random sub-sample was taken from the morphologically pre-sorted samples for molecular species determination. For this reason, the calculation of parasitological parameters according to Bush *et al.* [41] was omitted in the case of Nematoda.

Anisakidae were distinguished using internal transcribed spacers (ITS-1, 5.8 s, ITS-2) as described Zhu *et al.*, Shih and Klimpel *et al.* [44–47], using primers NC5 and NC2. For Cystidiocolidae specimens primers flanking the ribosomal small subunit (SSU) were designed using Geneious 8.17 software (f 93 5′-CCA ACG TGG ATA ACT GTG GT-3′; r 880 5′-CTC TCA CGC AGC GAT ACG AA-3′). The PCR was performed in 30 cycles with 60 s initiation at 95 °C, 30 cycles of 45 s denaturation at 94 °C, 45 s hybridisation at 52 °C, 45 s elongation at 72 °C and a 10 min final extension at 72 °C. Sanger sequencing was performed at Seqlab (Göttingen). A multiple alignment with sequences deposited in NCBI Genbank was performed using nBLAST [48]. Sequencing data are given in the supplemental information (Data S1).

## Results

### Host Diet

The digestion stage of the stomach contents of the three fish species varied. While there was mostly mucus detected in the stomachs of *Nototheniops larseni* and *L. nudifrons,* most food items of *L. squamifrons* were assigned to a taxonomic group (Table S2).

The sample of *N. larseni* (*n* = 40) included 18 stomachs with defined contents. The prey predominantly consisted of Crustacea (IRI = 18,041). Euphausiacea were identified as a food item and one specimen had preyed on fish.

The stomach contents of most samples of *N. nudifrons* (*n* = 40) were undefined. The contents of 7 stomachs could be assigned to a taxon, the rest contained undefined mucus. Based on these limited findings, Crustacea (IRI = 6409) were the most important food item of *N. nudifrons*, followed by benthic Mollusca (IRI = 3590).

Almost all food items of *L. squamifrons* (*n* = 49) could be identified. The most important group was Crustacea (IRI = 13,379, *F* = 97.62%), where Amphipoda and Euphausiacea were the most frequent (both taxa with *F* = 59.52%). Other rarer food items of *L. squamifrons* were Isopoda, Ostracoda, Mollusca (Gastropoda and Bivalvia), Polychaeta and Teleostei.

### Parasite Fauna

**Fig. 2:**
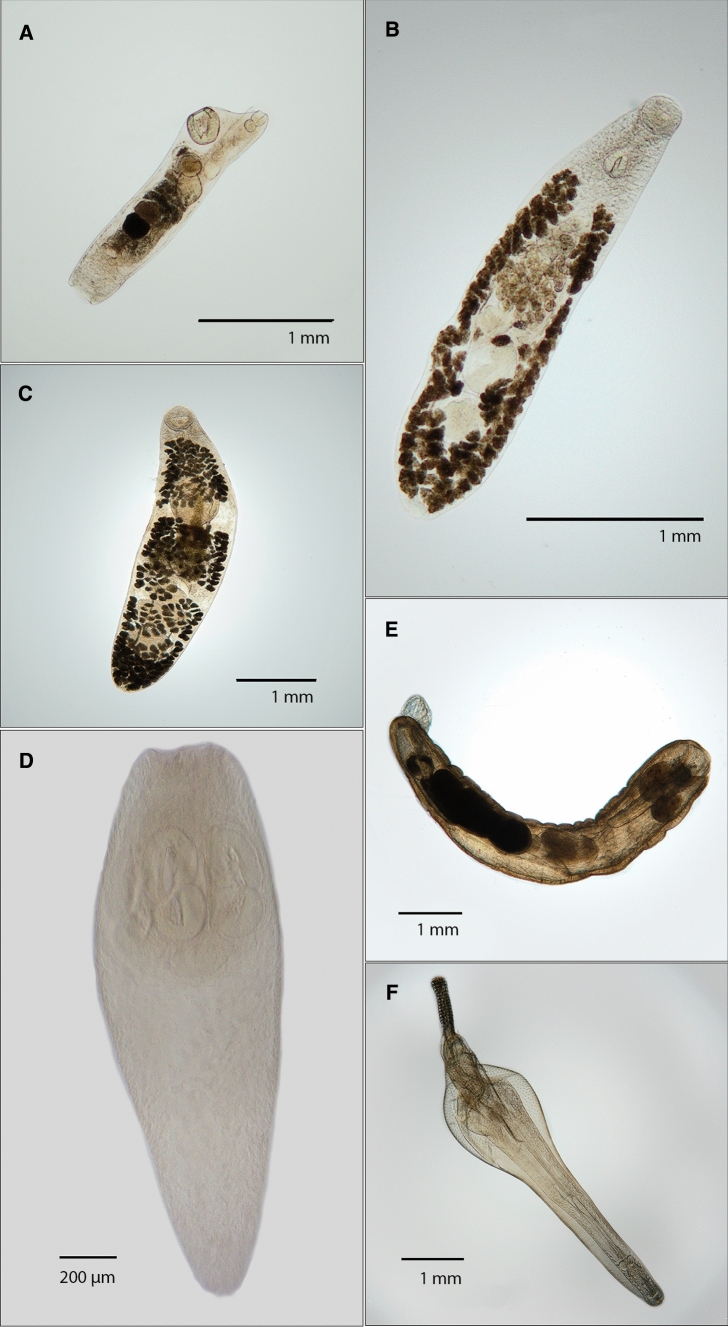
Parasites of notothenioid fishes from Elephant Island. **A** *Elytrophalloides oatesi*, **B** *Lepidapedon garrardi*, **C** *Neolebouria antarctica*, **D** Cestode cercoid with bilocular acetabula, **E** *Echinorhynchus petrotschenkoi*, **F** *Corynosoma bullosum* (colour figure online)

The parasite fauna of the three *Nototheniinae* spp. included the taxa Digenea, Acanthocephala, Cestoda and Nematoda. The three host species shared several parasites with different prevalences and intensities (Table 1). The digenean *Neolebouria antarctica* (Szidat & Graefe, 1967; Zdzitowiecki, 1990), the acanthocephalan *Corynosoma bullosum* (Linstow, 1892; Railliet & Henry 1907) and nematode *Pseudoterranova decipiens* E were detected in all host species. Photographs of the parasites are shown in Fig. 2.

**Table 1:** Parasites of *Nototheniops larseni*, *N. nudifrons* and *Lepidonotothen squamifrons* from the SSI

Host	Parasite	*n*	*P* [%]	*I*	mI	mA
*Nototheniops larseni*	Digenea	5	5	1–2	2.5	0.125
(*n* = 40)	Digenea indet	3	5	1–2	1.5	0.075
	*Neolebouria antarctica*	2	2.5	2	2	0.05
	Nematoda	499	97.5	1–52	12.79	12.48
	Acanthocephala	49	60	1–6	2.04	1.225
	Acanthocephala indet	1	2.5	1	1	0.025
	*Corynosoma* spp.	27	42.5	1–5	1.59	0.675
	*C. bullosum*	6	15	1	1	0.15
	*Echinorhynchus petrotschenkoi*	4	7.5	1–2	1.33	0.1
	*Metacanthocephalus* spp.	11	15	1–6	1.83	0.275
*Nototheniops nudifrons*	Digenea	11	7.5	1–4	3.67	0.275
(*n* = 40)	Digenea indet	5	5	2–3	2.5	0.125
	*Lepidapedon garrardi*	1	5	1	1	0.05
	*Neolebouria antarctica*	4	2.5	4	4	0.1
	Nematoda	503	100	1–38	12.58	12.58
	Acanthocephala	226	90	1–34	6.28	5.65
	Acanthocephala indet	51	50	1–7	2.55	1.275
	*Corynosoma* spp.	10	12.5	1–4	2	0.25
	*C. bullosum*	1	2.5	1	1	0.025
	*Metacanthocephalus* spp.	164	82.5	1–34	4.97	4.1
*Lepidonotothen squamifrons*	Digenea	78	59.2	1–11	2.69	1.591
(*n* = 49)	Digenea indet	50	40.8	1–11	2.5	1.02
	*Elytrophalloides oatesi*	1	2.04	1	1	0.02
	*Lepidapedon garrardi*	17	16.3	1–6	2.13	0.346
	*Neolebouria antarctica*	10	14.3	1–34	1.43	0.204
	Nematoda	437	100	1–36	8.92	8.92
	Cestoda	8	4.08	2–6	4	0.163
	Acanthocephala	298	89.8	1–19	6.77	6.081
	Acanthocephala indet	19	22.4	1–3	1.72	0.387
	*Corynosoma* spp.	142	73.5	1–19	3.94	2.897
	*C. bullosum*	127	73.5	1–10	3.53	2.591
	*Metacanthocephalus* spp.	10	16.3	1–3	1.25	0.204

Quantity = *n*, Prevalence = *P* [%], (mean) Intensity = (m)I, mean Abundace = mA

Taxon groups are underlined to better distinguish the parasitological parameters of the whole group from parasitological parameters of specimens that were not identified to a more specific taxon

Digenea were isolated from the alimentary tract of the host specimens. Three different species were identified in *Lepidonothothen squamifrons*, *Neolebouria antarctica*, *Lepidapedon garrardi* (Leiper & Atkinson, 1914; Manter, 1926) and *Elytrophalloides oatesi* (Leiper & Atkinson, 1914; Szidat & Graefe, 1967). *Lepidonotothen squamifrons* had the highest prevalence and diversity of Digenea. The specimens of *Nototheniops nudifrons* were infected with at least two different digenean species, *Neolebouria antarctica* and *Lepidapedon garrardi*. The lowest number of digeneans were isolated from *Nototheniops larseni*. Here, *Neolebouria antarctica* was identified as well.

Nematoda was the taxon with the highest prevalence and intensities in all host species. Nematodes were identified in all samples of *Nototheniops nudifrons* and *L. squamifrons*. *Nototheniops larseni* was the host with the highest infection intensity, one specimen was infected with 52 nematodes. The subsample of nematodes used for molecular barcoding revealed an infection with *P. decipiens* E and *Contracaecum osculatum* s.l. in all three host species (Table 2). *Ascarophis nototheniae* (Johnston & Mawson, 1945) was exclusively detected in the stomach of *N. nudifrons*. *Contracaecum osculatum* D was identified in *L. squamifrons*.

**Table 2:** Nematoda subsamples from *Nototheniops larseni*, *N. nudifrons* and *Lepidonotothen squamifrons*, stating number of hosts, number of parasites and NCBI-Accession numbers (Ref. ID)

Host	*Ascarophis nototheniae*		*Contracaecum osculatum* s.l		*Contraceacum osculatum* D		*Pseudoterranova decipiens* E	
	*n*	Ref. ID	*n*	Ref. ID	*n*	Ref. ID	*n*	Ref. ID
*Nototheniops larseni* (*n* = 27)	–	–	–	–	7	KY275507.1, MG787548.1	27	KF017610.1, KX378173.1, KX378174.1
*Nototheniops nudifrons* (*n* = 20)	13	DQ094172.1	3	KY275507.1	–	–	6	KX378173.1, KX378174.1
*Lepidonotothen squamifrons* (*n* = 19)	–	–	5	MT258528.1	2	KY275507.1, MG787549.1	12	KF017610.1, KX378173.1, KX378174.1

Cestoda only occurred in one host species. Unidentified tetraphyllidean larvae were isolated from two host specimens of *L. squamifrons*.

Acanthocephala were the second most frequent parasite taxon in all hosts, preceded by the Nematoda. *Nototheniops nudifrons* had the highest prevalence, followed by *L. squamifrons* and *N. larseni*. The parasites occurred as adults (*Echinorhynchus petrotschenkoi* (Rodjuk, 1984, Zdzitowiecki, 1989) and *Metacanthocephalus* spp.) or cystacanth stages (*Corynosoma* spp.).

Each fish species had some parasites that were either exclusive in the sample or occurred more frequently than in the other host species, especially from the group Acanthocephala. With an infection of *Echinorhynchus petrotschenkoi* in the stomach, *N. larseni* was the only host species infected by this parasite. *Nototheniops nudifrons* had the highest prevalence and infection intensities by the genus *Metacanthocephalus*, which occurred in the pyloric caeca and intestine. The highest prevalence of *Corynosoma bullosum* was detected in *L. squamifrons.*

**Fig. 3:**
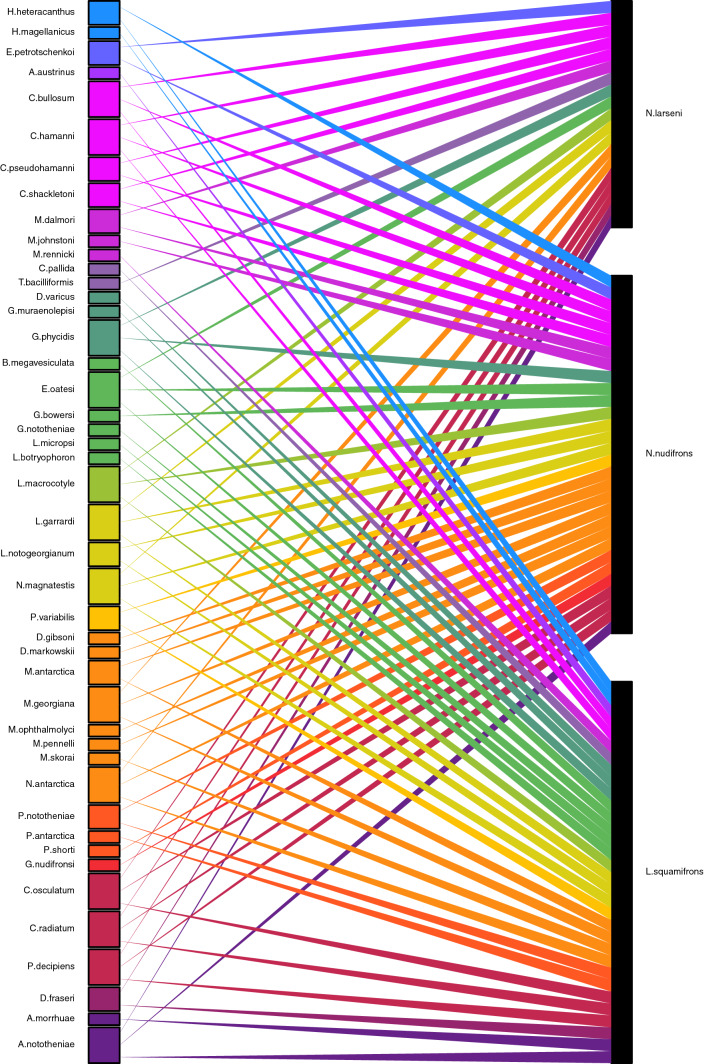
Reported parasites of *Nototheniops larseni, N. nudfrons* and *Lepidonotothen squamifrons* represented as bipartite network plot. Ray and box color indicates the parasite family, box size increases with the number of connections (colour figure online)

**Fig. 4:**
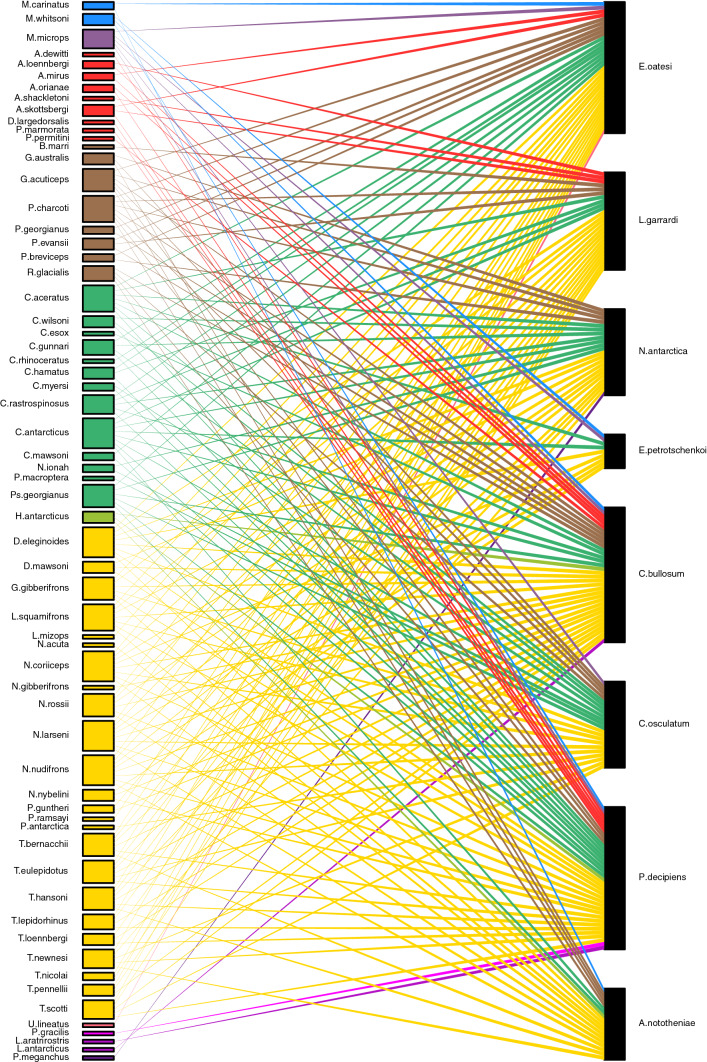
Reported host spectrum of the parasites of *Nototheniops larseni, N. nudifrons* and *Lepidonotothen squmaifrons* detected in this study represented as a bipartite network plot. Ray and box color indicates the host family, box size increases with the number of connections (colour figure online)

Parasite records of *Nototheniops larseni*, *N. nudifrons* and *Lepidonotothen squamifrons* and host spectrum of the identified parasites collated from literature are presented in Figs. 3 and 4, references of the records are stated in Tables S3 and S4.

## Discussion

Prey organisms can vary with the habitat of the fish and determine which parasites they ingest through their diet. Both the life cycles and infestation patterns of the parasites are used to gain insight into the ecology of the fish studied. We here discuss aspects of diet and parasitisation patterns of Antarctic fish (Nototheniinae), focusing on the three species, *Nototheniops larseni*, *N. nudifrons* and *Lepidonotothen squamifrons* and the results gained through our sampling and a current literature review.

### Diet

Dietary analyses depend on the food consumed directly before and digestion degree at the point of sampling. Indigestible components, bones, exoskeletons, shells, bristles, etc., can be used for morphological identification. Fish from the same size group caught in a single haul usually contain similar food items and digestion stages.

In this study, the stomach contents of *Nototheniops larseni* and *N. nudifrons* were in an advanced digestion stage, which resulted in a low sample and statistically unreliable calculations of dietary parameters, overestimating proportions and importance of food components. However, the few food items identified did not contradict previous findings.

Stomach contents of *N. larseni* were mostly identified as Euphausiacea and Teleostei (rare). These findings agree with the reported main food source of *N. larseni* being krill (*Euphausia superba*), amphipods (*Probolisca ovata, Gitanopsis squamosa, Oradarea bidentata, Prostebbingia gracilis, P. brevicornis, Paramoera* spp.) and copepods [49–51]. The food composition (krill, hyperiids, copepods, young fish), reports of benthic amphipoda and stones in the stomach of *N. larseni* indicate an epibenthic lifestyle [13, 51]. A positive connection between fish size and increasing consumption of Mysida (e.g. *Antarctomysis maxima*) and hyperiid Amphipoda (*Parathemisto gaudichaudii*) has been observed [50].

The discovered food items of *N. nudifrons* from this study point to a benthic feeding strategy, agreeing with previous research [13]. Other studies described the diet composition of *N. nudifrons* as a typical secondary consumer with an increasing number of different prey taxa with increasing total length [52, 53]. Previous research suggests an ontogenetic shift of the diet from cyclopoid and calanoid copepods (e.g. *Paraeuchaeta antarctica* and *Pleuromamma. gracilis*) and Mysida to benthic invertebrates, amphipods, polychaetes and isopods, but also krill (*E. superba*), and shrimp (*Crangon antarcticus, Chorismus antarcticus*) [13, 50, 52, 54, 55].

A variation of food components at different sites has been described for *N. larseni* and *N. nudifrons*, adapting to the availability of food items, related to the respective habitat conditions [52, 56–60].

The stomach contents of *L. squamifrons* were preserved best in this study, hence the highest diversity of food items. The higher variety of food organisms in adult specimens compared to subadult samples of *L. squamifrons* from this study indicate a size and maturity-related ontogenetic shift in feeding behaviour, as previously observed in other Nototheniinae [52]. Other studies found the diet of *L. squamifrons* to be similar to *N. larseni* [61]. Both fishes feed predominantly on krill, with a lower proportion of benthic invertebrates and *L. squamifrons* also feed on fish [61]. The reported feeding behaviour of *L. squamifrons* includes demersal and pelagic hunting [13]. Salps have been reported to be a component of *L. squamifrons* diet [13], which could be advantageous in the light of a growing salp population due to the increase of water temperatures [5, 62–64].

Krill is a dominant diet component of demersal fish summer diet at the South Shetland Islands region, and its biomass is positively correlated with demersal fish abundance. The reported distribution of krill in the water column reaches the bottom, which agrees with a demersal lifestyle of krill consuming fish species [61, 65–68]. The proportion of Amphipoda and krill in the demersal fish diet varies seasonally, more energy-rich Amphipoda are consumed during winter and krill during summer [13]. Our data support the importance of krill and amphipods in the diet of all examined fish species. Literature data agree with diet variations depending on size (ontogenetic shift) and between different sampling sites.

### Parasites of the Examined Nototheniinae

#### Digenea

In our study, infections with Digenea were rare in *Nototheniops larseni* and *N. nudifrons* and more frequent in *Lepidonotothen squamifrons*. Most known digeneans found in the Southern Ocean infect fish hosts associated with a benthic habitat. An increase in infection intensity of fish hosts with digeneans, but a site effect with a decrease in taxon diversity has been described in coastal areas compared to offshore sites [29].

*Elytrophalloides oatesi* (Hemiuridae) is frequently detected in Antarctic fish (e.g. *N. nudifrons*) [31] but it is not endemic to the Southern Ocean [69] and the reported host range is wide [37, 69, 70]. It is not host-specific to Nototheniidae and it does not imply a restriction of the host to the Southern Ocean. *Elytrophalloides oatesi* could be considered an indicator of a benthopelagic fish host, the life cycle of Hemiuridae includes a pelagic intermediate host [71, 72]. This is supported by our findings on the diet and parasitisation of *L. squamifrons*.

The general distribution and ecology of *Lepidapedon garrardi* (Lepocreadiidae) is similar to *E. oatesi*, as it occurs in Notothenioidei and is found throughout the Southern Ocean and Sub-Antarctic regions [29, 70]. The genus includes 30 species [72, 73]. In contrast to our findings, *L. garrardi* was the dominant digenean of *Nototheniops nudifrons* at Admiralty Bay, occurring with high intensities to 131 parasites per host [31]. This difference could be owed to a site effect between King George Island and Elephant Island. This effect had been described for *L. garrardi* infection of *T. bernacchii*, which could also apply to *L. garrardi* and *N. nudifrons* in this study [74].

*Neolebouria antarctica* (Opecoelidae) was detected in all host species examined in this study and is frequently found in Notothenioidei and Liparididae (Table S4, [29]). Its distribution covers West Antarctica and South Georgia, while its congener *N. terranovensis* is found in East Antarctica (Weddell and Ross Sea, Indian Sector) [29]. The life cycle of *N. antarctica* includes a metacercariae stage in crustaceans [70, 75], which could play an important role in the transmission to fishes. Its infection parameters seem to vary with site and host species. The low prevalence in *N. nudifrons* detected in this study agrees with findings from Laskowski & Zdzitowiecki [31] from Vernadsky Station (Argentine Islands, Antarctic Peninsula).

The aforementioned Digenea have all been previously detected in the examined host species (Table S3), except *Lepidapedon garrardi* in *Lepidonotothen squamifrons*, for which we provided a new host record.

#### Nematoda

Nematodes were the most prevalent and numerous parasites of the examined Nototheniidae. The nematode infection of the examined fishes is connected to their demersal lifestyle. The similarities of the euryxenous parasites of the three host species are also reflected within this group, including larval stages of the anisakids *Pseudoterranova decipiens* (s.l.) and *Contracaecum osculatum* (s.l.). Considering that we molecularly identified a subsample as *Pseudoterranova decipiens* E, which is the only species from the *P. decipiens* complex occurring in Antarctic fishes [76], it can be assumed that the parasites identified as *P. decipiens* (s.l.) are most likely *P. decipiens* E. This species has been reported to be characteristic of the lower Antarctic shelves of the seasonal pack ice zone [77]. *Pseudoterranova decipiens* E requires a pinniped definitive host, specifically the Weddell Seal (*Leptonychotes weddellii*) [76, 78]. The occurrence of *P. decipiens* E is limited by depth, they are found at the shallow shelf, because deep waters are not frequented by hunting Weddell seals [77]. *Pseudoterranova decipiens* E is restricted to benthic or benthopelagic fish hosts, it does not occur in pelagic fishes [77, 79]. Most seal species hunt in the water column [10, 80, 81] but isotopic analyses by Burns *et al.* [82] showed that *Leptonychotes weddellii* also consumes benthic prey. A more recent study by Daneri *et al.* [83] analysed the fish prey of *L. weddellii* at Hope Bay and showed that its main food were Nototheniidae (80%). *Nototheniops larseni* seemed to be an important food item (normalised IRI = 13.9%), while *N. nudifrons* and *Lepidonotothen squamifrons* were rarer prey of *Leptonychotes weddellii* [83]. The parasitological data from our study suggest that all examined fish hosts contribute to the transmission of *P. decipiens* E to its definitive host at the respective sampling site.

*Contracaecum osculatum* (s.l.) also uses seals as definitive host. Co-infections of the fish host, as observed in this study, are common, and suggestions of *Contracaecum* spp. outcompeting *Pseudoterranova* spp. in the definitive hosts have been questioned [25]. Similar to *Pseudoterranova decipiens* E, the life cycle of *Contracaecum osculatum* involves demersal intermediate hosts, while its congener *C. radiatum* (not detected in this study) is found in pelagic intermediate hosts [84].

Since anisakid nematodes tend to be less specific regarding paratenic fish hosts [85], they have been recorded in a wide range of hosts from the Southern Ocean.

The cystidicolid nematode *Ascarophis nototheniae* was only detected in *N. nudifrons* in this study. It is one of six Antarctic nematodes maturing in fish and is common in Notothenioidei, especially Nototheniidae [86]. *Ascarophis* spp. have been described to use Decapoda as their intermediate host [87]. This indicates that *N. nudifrons* infected by *A. nototheniae* have been feeding on its reported benthic shrimp hosts, such as *Chorismus antarcticus* and *Notocrangon antarcticus* [88].

#### Acanthocephala

Acanthocephala were abundant parasites of the examined fish species. While *Corynosoma* spp. were more frequent in *Nototheniops nudifrons* and *Lepidonotothen squmaifrons, Metacanthocephalus* spp. were more prevalent in *N. nudifrons*. The detection of *E. petrotschenkoi* in two specimens of *N. larseni* represents a new host record.

All Acanthocephala maturing in Antarctic fish have two hosts within their life cycle, if the definitive hosts are birds or seals, their life cycle includes three hosts [42, 70]. Antarctic acanthocephalans are frequent in demersal fish hosts and absent in fishes with a pelagic lifestyle, which suggests a benthic intermediate host and a bottom-feeding definitive host [42, 70]. Some Acanthocephala are typical for the region south of the ACC (lower Antarctica), *Metacanthocephalus johnstoni*, *M. dalmori*, *Aspersentis megarhynchus*, *Echinorhinchus petrotschenkoi, E. muranolepis, Corynosoma bullosum, C. arctocephali, C. hamanni, C. shackletoni* [70]. Most Acanthocephala found in the Southern Ocean are more common in coastal habitats and fish hosts acquire the parasite if they are associated to this habitat permanently or during specific life-history events, e.g. spawning [28].

The life cycle of *Corynosoma* spp. at the South Shetland Islands (Admiralty Bay) involves Amphipoda as intermediate hosts [89]. Cystacanths of *Corynosoma hamanni* and *C. pseudohamanni* were detected in *Prostibbingia brevicornis* and *Cheirimedon femoratus, Corynosoma bullosum* occurred in *Waldeckia obesa* and *Bovallia gigantea* [89]. *Corynosoma* spp. have a wide range of teleost hosts ([90], Table S4). *Corynosoma bullosum* is specific to its definitive host, the Southern Sea Elephant *Mirounga leonina* [77, 91], occurring at the lower latitudes of Antarctica, around the Antarctic Peninsula, SSI and Sub-Antarctica [92]. The definitive hosts of *Corynosoma pseudohamanni*, Crabeater Seals and Weddell Seals, only occur in higher Antarctica [93]. The reported abundance of *C. bullosum* at shelf and offshore sites matches our findings at shelf sampling sites around Elephant Island. *Corynosoma pseudohamanni* and *C. hamanni* rarely occur in fishes from shelf areas [28]. The infection of *Nototheniops larseni* and *N. nudifrons* and *Lepidonotothen squamifrons* with *Corynosoma* spp. points to a trophic connection as prey of pinnipeds.

The definitive hosts of *Metacanthocephalus* spp. (Rhadinorhynchidae) are fish [94, 95]. The genus occurs in 2–3 families belonging to the Notothenioidei [42, 70]. *Metacanthocephalus johnstoni* occurs in *Cheirimedon femoratus,* while *M. dalmori* has been found in deeper living Crustacean hosts, indicating different depth ranges [94]. Zdzitowiecki and Laskowski [96] reported that the infection intensity of *N. nudifrons* with Acanthocephala had increased compared to findings from 1978/1979. Similar to our findings, Rhadinorhynchida (*Metacanthocephalus* spp.) were more numerous in *N. nudifrons* than Polymorphida (*Corynosoma* spp.) [96].

*Echinorhynchus* spp. are known as parasites of gadiform fishes [28]. *Echinorhynchus petrotschenkoi* occurs in the Antarctic species from this taxonomic order, *Muraenolepis microps* and *M. whitsoni* (Macrouridae), but has also been reported in various Nototheniidae, e.g. *Nototheniops nudifrons* [28, 70, 97]. A connection between the infection intensity of *Echinorhynchus* sp. in nototheniids to the diversity of Crustacea in their habitat has been shown [74]. The prevalence of *Echinorhynchus* spp. is unusual in fjords and near shore habitats [28], the infection of *N. larseni* was acquired from an intermediate host at an offshore site and could be an indicator for migrations.

#### Parasite Specificity

A common feature of all parasites infecting hosts from the present study is their low host specificity regarding the teleost host. All parasites have a host spectrum including more than one taxonomic family, qualifying as ‘euryxenous’ [98]. Most hosts belong to the suborder Notothenioidei and the family Nototheniidae, which constitute the largest proportion of Antarctic fish diversity. Rohde and Heap [99] stated that the parasite fauna of one Antarctic fish species could be dominated by different parasite taxa, resulting in individual-specific parasitisation patterns.

Including previous records on the parasite fauna of the three species examined in this study (see Table S3), there seems to be a connection with parasite diversity and sampling effort (number of host species and number of sampling sites) but also host size, since the larger species *Lepidonotothen squamifrons* has a lot more parasite records than the smaller species *Nototheniops larseni*, which has also been less studied. The parasite diversity reported in *L. squamifrons* is probably owed to a higher trophic level and a sampling bias in favour of larger fish species. The parasite fauna of the Southern Ocean's largest teleosts, *Dissostichus* spp., is well studied due to commercial interest in these species as a fishery resource [32]. Possibly due to the top position in the food web and also a larger number of studies, a large number of parasites have been described for *Dissostichus* spp. (357 entries in Oğuz *et al.* [37]).

Observations of site effects on parasite patterns in Antarctic Nototheniidae have been made in previous studies. A site variability of infection patterns with Digenea and Acanthocephala has previously been described for *N. nudifrons* [31] and other Nototheniids [100] and could explain observations made in this study. Laskowski & Zdzitowiecki [31] found a site effect comparing the infection of *N. nudifrons* with the digeneans *Lepidapedon garrardi*, *Elytrophalloides oatesi* and *Neolebouria antarctica*. Also, the infection with the acantocephalan *Corynosoma pseudohamanni* varied between sites. This suggests that these parasites might use different macroinvertebrate hosts that thrive differently at the respective sampling sites. Opportunistic food choice and adaptation to different feeding strategies of the fish hosts resulting from the structural features (rocky, sandy, slope) of a site could also result in varying infection intensities between studies. Studies by Moser and Cowen [74] and Münster *et al.* [101] observed site effects in the parasite communities of *Trematomus bernacchii* and *Macrourus whitsoni*. The design of future studies using fish parasite communities as indicators of ecosystem changes needs to account for site effects.

## Conclusion

Polar ecosystems are currently subject to the serious effects of climate change. Parasite communities and their patterns and distributions in particular can be used for the management of Antarctic marine resources and the monitoring of climate change effects on biotic communities. However, biomonitoring of parasite communities in model host species should take into account potential site effects on infection numbers. Thus, despite the challenges and limited opportunities for fishing in these remote areas, biomonitoring must ideally be carried out periodically in a predefined range of habitats and sites, including as many levels as possible, to be able to track, compare, and better understand changes in the future.

## Supplementary Information

Below is the link to the electronic supplementary material.

Supplementary file1 (DOCX 187 KB)
