# Supplementary material for: Parasites of Three Closely Related Antarctic Fish Species (Teleostei: Nototheniinae) from Elephant Island
Source: Acta Parasitol. 2021 Jul 17;67(1):218–32. doi: 10.1007/s11686-021-00455-8 (PMC8938359; doi:10.1007/s11686-021-00455-8)
Supplement: Supplementary file 1 — Supplementary file1 (DOCX 187 KB) [file 11686_2021_455_MOESM1_ESM.docx]

**Parasites of three closely related Antarctic fish species (Teleostei: Nototheniinae) from Elephant Island**

Acta Parasitologica

Katharina G. Alt^1*^, Sarah Cunze^1^, Judith Kochmann^2^, Sven Klimpel^1,2^

^1^ Goethe University, Institute for Ecology, Evolution and Diversity, D-60438 Frankfurt/Main, Germany

^2^ Senckenberg Biodiversity and Climate Research Centre, Senckenberg Gesellschaft für Naturforschung, D-60325 Frankfurt/Main, Germany

*Corresponding author.

E-Mail: alt@bio.uni-frankfurt.de

**Table S1:** Sampling sites of *Nototheniops larseni*, *N. nudifrons* and *Lepidonotothen squamifrons* off Elephant Island (Antarctica).

| Haul | Latitude | Longitude | Sampled species | Depth1 [m] | Depth2 [m] |
| --- | --- | --- | --- | --- | --- |
| 188 | 61° 11,22' S | 54° 35,30' W | *N. larseni, L. squamifrons* | 277.5 | 355.9 |
| 190 | 61° 12,00' S | 54° 52,49' W | *N. nudifrons* | 71.3 | 52.8 |
| 206 | 60° 49,77' S | 55° 37,25' W | *L. squamifrons* | 479.7 | 470.6 |

**Table S2:** Food items of *Nototheniops larseni*, *N. nudifrons* and *Lepidonotothen squamifrons.* Frequency of occurrence (F[%]), numerical percentage of prey (N[%]), weight percentage of prey (W[%]) and index of relative importance (IRI).

| Fish species | Full stomachs | Food item | F[%] | N[%] | W[%] | IRI |
| --- | --- | --- | --- | --- | --- | --- |
| *Nototheniops larseni* (n=40) | 18 |  |  |  |  |  |
|  |  | Crustacea | 94.44 | 97.78 | 93.25 | 18041.13 |
|  |  | Euphausiacea | 22.22 | 11.11 | 26.25 | 830.32 |
|  |  | Teleostei | 5.56 | 2.22 | 6.75 | 49.87 |
| *N. nudifrons* (n=40) | 8 |  |  |  |  |  |
|  |  | Crustacea | 50.00 | 50.00 | 78.20 | 6409.92 |
|  |  | Amphipoda | 12.50 | 12.50 | 1.28 | 172.21 |
|  |  | Euphausiacea | 25.00 | 25.00 | 73.30 | 2457.38 |
|  |  | Mollusca | 50.00 | 50.00 | 21.80 | 3590.08 |
|  |  | Bivalvia | 37.50 | 37.50 | 19.73 | 2145.99 |
| *Lepidonotothen squamifrons* (n=49) | 42 |  |  |  |  |  |
|  |  | Crustacea | 97.62 | 89.32 | 47.75 | 13379.89 |
|  |  | Amphipoda | 59.52 | 56.16 | 4.96 | 3638.14 |
|  |  | Euphausiacea | 59.52 | 17.81 | 26.20 | 2643.44 |
|  |  | Isopoda | 14.29 | 2.74 | 3.63 | 90.93 |
|  |  | Ostracoda | 4.76 | 0.55 | 0.02 | 2.69 |
|  |  | Mollusca | 11.90 | 1.37 | 11.51 | 153.35 |
|  |  | Bivalvia | 7.14 | 0.82 | 11.21 | 85.92 |
|  |  | Gastropoda | 2.38 | 0.27 | 0.30 | 1.38 |
|  |  | Polychaeta | 7.14 | 0.82 | 0.44 | 8.99 |
|  |  | Teleostei | 7.14 | 0.82 | 40.30 | 293.76 |

**Table S3:** List of parasites of *Nototheniops larseni*, *N. nudifrons* and *Lepidonotothen squamifrons*. Synonyms included in the list are *Notothenia larseni, Lepidonotothen larseni* for *Nototheniops larseni; Notothenia nudifrons, Lepidonotothen nudifrons, Lindbergichthtys nudifrons, Notothenia mizops nudifrons* for *Nototheniops nudifrons* and *Notothenia squamifrons, Lepidonotothen kempi, Notothenia brevipectoralis, Notothenia kempi, Notothenia macrophthalma* for *Lepidonotothen squamifrons*. Region abbreviations: LB = Lena Bank, OB = Ob Bank, SGI = South Georgia Island, SSI = South Shetland Islands, PF = Port Foster (Deception Island), AB = Admiralty Bay, KGI = King George Island, EI = Elephant Island, SG = South Georgia, BI = Bouvet Island, SOI = South Orkney Islands, PEI = Prince Edward Island, MI = Marion Island, CI = Cozet Islands, KS = Kerguelen Subregion, VS = Vernadsky Station, LI = Loinville Island, HI = Heard Island, McI =Macquarie Island, PB = Prydz Bay, SB = Skif Bank, NSR = North Scotia Ridge, SR = Shag Rocks, KI = Kherd Island. Compiled from a checklist by Oğuz et al 2015^1^, the Host-parasite database of the NHM London^2^ and Google Scholar search of publications since 2015; the taxonomy was checked in WoRMS 2020 and updated.

| Host | Group | Family | Species | Region | Ref. |
| --- | --- | --- | --- | --- | --- |
| *Nototheniops larseni* | Digenea | Derogenidae | *Gonocerca phycidis* | LB, OB | ^3^ |
|  |  | Hemiuridae | *Elytrophalloides oatesi* | SGI | ^4^ |
|  |  |  | *Lecithochirium* sp. | OB | ^3^ |
|  |  | Lecithasteridae | *Lecithaster macrocotyle* | SSI | ^5^ |
|  |  | Lepocreadiidae | *Lepidapedon garrardi* | PF, SGI | ^4,6^ |
|  |  |  | *Neolepidapedon magnatestis* (as syn. *Opechona magnatestis*) | LB | ^3^ |
|  |  | Opecoelidae | *Macvicaria georgiana* | PF | ^6^ |
|  |  |  | *Neolebouria antarctica* | PF, SGI, AB, KGI, EI, SG, SSI | ^4,6,7^ |
|  | Cestoda | Oncobothriidae | Unspecified Pseudophyllidea sp. | SSI | ^5^ |
|  |  | Diphyllobothriidae | Unspecified plerocercoid | PF | ^6^ |
|  |  | Phyllobotrhiidae | Phyllobothrium cercoid V | SGI | ^8^ |
|  |  | Tetraphyllidae | Unspecified larvae type „Scolex pleuronectis“ | LB, OB | ^3^ |
|  |  |  | Unspecified Tetraphyllidae gen. sp. | LB, OB, PF | ^3,6^ |
|  | Acanthocephala | Polymorphoidea | unspecified | SGI | ^9^ |
|  |  |  | *Corynosoma* spp. | SSI | ^5^ |
|  |  |  | *Corynosoma bullosum* (as syn. *C. arctocephali*) | PF, SGI, SSI | ^5,6,9^ |
|  |  |  | *Corynosoma hamanni* | PF, LB, SSI | ^3,5,6^ |
|  |  |  | *Corynosoma pseudohamanni* | PF | ^6^ |
|  |  |  | *Corynosoma shackletoni* | SSI | ^5^ |
|  |  | Rhadinorhynchidae | *Metacanthocephalus* sp. | PF, SSI | ^5,6^ |
|  |  |  | *Metacanthocephalus dalmori* | SSI | ^5^ |
|  | Nematoda | Anisakidae | *Contracaecum* sp. | LB, OB | ^6^ |
|  |  |  | *Contracaecum osculatum* (s.l.) | SSI | ^5^ |
|  |  |  | *Contracaecum radiatum* | SSI | ^5^ |
|  |  |  | *Pseudoterranova decipiens* (s.l.) | SSI, PF | ^5,6,10^ |
|  |  | Cucullanidae | *Dichelene (Cucullanellus) fraseri* (as syn. *Cucullanellus fraseri*) | LB | ^3^ |
|  |  | Cystidicolidae | *Ascarophis nototheniae* | SSI | ^5^ |
|  | Annelida | Piscicolidae | *Trulliobdella bacilliformis*  (valid?) | BI, SSI, SOI, SGI, PEI, MI, CI, KS | ^11^ |

| Host | Group | Family | Species | Region | Ref. |
| --- | --- | --- | --- | --- | --- |
| *Nototheniops nudifrons* | Monogenea | Capsalidae | *Pseudobenedenia nototheniae* |  | ^12^ |
|  |  |  | *Pseudobenedenoides shorti* |  | ^12^ |
|  |  | Gyrodactylidae | *Gyrodactylus* sp. | AB, SSI | ^13^ |
|  |  |  | *Gyrodactylus nudifronsi* | AB | ^13^ |
|  | Digenea | Derogenidae | *Gonocerca phycidis* | SGI, VS | ^4,14^ |
|  |  | Hemiuridae | *Elytrophalloides oatesi* | SGI, VS, AB | ^4,14^ |
|  |  |  | *Genolinea bowersi* | SSI, SGI, VS, AB | ^4,14,15^ |
|  |  | Lecithasteridae | *Lecithaster macrocotyle* | SGI, AB, EI, LI | ^4,5,16^ |
|  |  | Lepocreadiidae | *Lepidapedon garrardi* | SSI, VB, AB | ^14,17^ |
|  |  |  | *Lepidapedon notogeorgianum* | SGI | ^4^ |
|  |  |  | *Neolepidapedon magnatestis* (as syn. *Opechona magnatestis*) |  | ^18^ |
|  |  | Monoorchiidae | *Postmonorchis variabilis* | SGI | ^4^ |
|  |  | Opecoelidae | *Discoverytrema gibsoni* | SGI | ^4^ |
|  |  |  | *Discoverytrema markowskii* |  | ^19^ |
|  |  |  | *Macvicaria antarctica* | SGI | ^4^ |
|  |  |  | *Macvicaria georgiana* (as *Macvicaria lobata georgiana*) | VS, AB | ^14^ |
|  |  |  | *Macvicaria ophthalmolyci* |  | ^20^ |
|  |  |  | *Macvicaria pennelli* | SG, SSI | ^21,22^ |
|  |  |  | *Neolebouria antarctica* | SSI, KGI, EI, SGI, VS | ^5,7,14^ |
|  | Cestoda | unspecified | Cercoid IV bilocular | SSI | ^8^ |
|  |  | Oncobothriidae | Unspecified Pseudophyllidea | SSI | ^5^ |
|  |  |  | *Onchobothrium antarcticum* |  | ^8^ |
|  |  | Diphyllobothriidae | Unspecified plerocercoid | PF | ^6^ |
|  |  | Phyllobothriidae | Phyllobothrium cercoid I | SSI | ^8^ |
|  |  |  | Phyllobothrium cercoid IV | SSI | ^8^ |
|  |  |  | Phyllobothrium cercoid V | SSI | ^8^ |
|  |  |  | Phyllobothrium cercoid VI | SSI | ^8^ |
|  | Acanthocephala | Arhythmacanthidae | *Heterosentis heteracanthus* | SGI | ^9^ |
|  |  | Echinorhynchidae | unspecified | SGI | ^9^ |
|  |  |  | *Echinorhynchus petrotschenkoi* | SG | ^22^ |
|  |  | Heteroacanthocephalidae | *Aspersentis austrinus* | SSI | ^23^ |
|  |  | Polymorphoidea | unspecified | SSI | ^9^ |
|  |  |  | *Corynosoma bullosum* (syn. *Corynosoma arctocephali*) | PF, SSI, SGI, VS | ^5,6,9,24^ |
|  |  |  | *Corynosoma hamanni* | SSI, PF | ^6,24^ |
|  |  |  | *Corynosoma pseudohamanni* | SSI, PF, AB, VS | ^6,14,24^ |
|  |  |  | *Corynosoma shackletoni* | SSI, SG | ^9^ |
|  |  | Rhadinorhynchidae | *Metacanthocephalus spp.* | PF, SSI | ^5,6^ |
|  |  |  | *Metacanthocephalus dalmori* | VS, AB | ^14^ |
|  |  |  | *Metacanthocephalus johnstoni* | SSI, VS, AB | ^14,24^ |
|  | Nematoda | Anisakidae | *Contracaecum* spp. | PF, VS, AB, KGI | ^6,14,25^ |
|  |  |  | *Contracaecum osculatum* | SSI | ^5^ |
|  |  |  | *Contracaecum radiatum* | SSI | ^5^ |
|  |  |  | *Pseudoterranova decipiens* (s.l.) | SSI, PF, KGI, AB, VS | ^6,10,14,25^ |
|  |  | Cystidiocolidae | *Ascarophis nototheniae* | SSI, VS, AB | ^5,14^ |

| Host | Group | Family | Species | Region | Ref. |
| --- | --- | --- | --- | --- | --- |
| *Lepidonotothen squamifrons* | Monogenea | Capsalidae | *Pseudobenedenoides antarctica* | HI, McI, LB, OB | ^3,26,27^ |
|  |  |  | *Pseudobenedenia nototheniae* |  | ^27^ |
|  |  | Gyrodactylidae | *Gyrodactylus* spp. | PB, HI, McI | ^27,28^ |
|  | Digenea | Derogenidae | *Derogenes varicus* | OB, KS | ^3,29,30^ |
|  |  |  | *Gonocerca muraenolepisi* | SB, OB, LB | ^29^ |
|  |  |  | *Gonocerca phycidis* | KS, SB, OB, LB | ^29,30^ |
|  |  | Hemiuridae | *Boreascotia megavesicula* | NSR | ^31^ |
|  |  |  | *Elytrophalloides oatesi* | OB, CI, SGI | ^3,4,29,30^ |
|  |  |  | *Genolinea nototheniae* (as syn. *Pseudobunocotyla nototheniae*) |  | ^20^ |
|  |  | Lecithasteridae | *Lecithaster macrocotyle* (as syn. *Lecithaster australis*) | KS, OB, LB, SB, SGI | ^4,29,30^ |
|  |  |  | *Lecithaster micropsi* | SR | ^16^ |
|  |  |  | *Lecithophyllum botryophoron* (as syn. *Lecithophyllum anteroporum*) | KS, SB, OB | ^3,29,30^ |
|  |  | Lepocreadiidae | *Lepidapedon notogeorgianum* | SGI | ^4,32^ |
|  |  |  | *Neolepidapedon magnatestis* (as syn. *Opechona magnatestis*) | NSR, SGI, SR, KS, CO, OB | ^3,29,30,32,33^ |
|  |  | Monorchiidae | *Postmonorchis variabilis* | LB, OB | ^3^ |
|  |  | Opecoelidae | *Macvicaria georgiana* | SR | ^29^ |
|  |  |  | *Macvicaria antarctica* | NSR, KS, CI, OB, LB | ^3,29,30,33^ |
|  |  |  | *Macvicaria skorai* | NSR | ^33^ |
|  |  |  | *Neolebouria antarctica* | AB, KGI, EI, SGI, SG, KGI, SSI | ^4,5,7^ |
|  | Cestoda | Oncobothriidae | Unspecified Pseudophyllidea | SSI | ^5^ |
|  |  |  | Unspecified Tetraphyllidae | KS, CI, SB, OB, LB | ^3,29,30^ |
|  |  |  | Unspecified Tetraphylidae (bilocular bothria) | HI | ^34^ |
|  |  | Diphyllobothriidae | Phyllobothrium cercoid II | SGI | ^8^ |
|  |  |  | Phyllobothrium cercoid IV | SGI | ^8^ |
|  |  |  | Phyllobothrium cercoid V | SGI | ^8^ |
|  |  | Tetraphyllidae | Unspecified type “Scolex pleuronectis” | KS, CI, SB, OB, LB, | ^3,29,30^ |
|  |  | Triaenophoriedae | *Eubothrium* sp. | KS, CI, SB, OB, LB | ^29,30^ |
|  | Acanthocephala | Arhythmacanthidae | *Heterosentis hetaracanthus* | SGI | ^9^ |
|  |  |  | *Hypoechinorhynchus magellanicus* | LB | ^3^ |
|  |  | Echinorhynchoidea | unspecified | LB | ^9^ |
|  |  | Heteroacanthocephalidae | *Aspersentis austrinus* (as syn. *Aspersentis megarhynchus*) | SSI, OB | ^3,5^ |
|  |  | Polymorphoidea | unspecified | OB, SSI | ^9^ |
|  |  |  | *Corynosoma* spp. | SSI | ^5^ |
|  |  |  | *Corynosoma bullosum* (also as syn. *Corynosoma arctocephali*) | SSI, SGI | ^5,9^ |
|  |  |  | *Corynosoma hamanni* | KS, CI, SB, LB | ^3,29,30^ |
|  |  | Rhadinorhynchidae | *Metacanthocephalus spp.* | SSI | ^5^ |
|  |  |  | *Metacanthocephalus rennicki* (as syn. *Echinorhynchus debenhami*) | KS, CI, OB, LB | ^29,30^ |
|  | Nematoda | Anisakidae | *Anisakis* sp. | KS, CI, SB, OB, LB | ^3,29,30^ |
|  |  |  | *Contracaecum* sp. | KS, CI, SB, OB, LB, KI | ^3,29,30^ |
|  |  |  | *Contracaecum osculatum* | SSI | ^5^ |
|  |  |  | *Contracaecum radiatum* | SSI | ^5^ |
|  |  |  | *Pseudoterranova decipiens* (s.l.) | SSI | ^10^ |
|  |  | Cucullanidae | *Dichelene (Cucullanellus) fraseri* (as syn. *Cucullanus fraseri* and var. *nototheniae*) | KS, CI, SB, OB, LB | ^29,30^ |
|  |  | Cystidicolidae | *Ascarophis morrhuae* | KS, CI, SB, OB, LB | ^30^ |
|  |  |  | *Ascarophis nototheniae* | SSI, KS, CI, SB, OB, LB, SG | ^3,29^ |
|  | Annelida | Unspecified | Unspecified Hirudinea |  | ^28^ |
|  |  | Piscicolidae | *Cryobdella* sp. | CI | ^29,30^ |
|  |  |  | *Cryobdella pallida* | CI | ^11^ |

**Table S4:** List of hosts of the parasites detected in this study. Compiled from a on a checklist by Oğuz et al 2015^1^, the Host-parasite database of the NHM London^2^ and Google Scholar search of publications since 2015; the taxonomy was checked in WoRMS 2020 and updated.

| Species | Host | Host Family | Ref |
| --- | --- | --- | --- |
| *Elytrophalloides oatesi* | *Artedidraco mirus* | Artedidraconidae (Notothenioidei) | ^4^ |
|  | *Artedidraco skottsbergi* |  | ^35^ |
|  | *Gymnodraco acuticeps* | Bathydraconidae (Notothenioidei) | ^36^ |
|  | *Parachaenichthys charcoti* |  | ^4,37,38^ |
|  | *Parachaenichthys georgianus* |  | ^4,15^ |
|  | *Prionodraco evansii* |  | ^39,40^ |
|  | *Psilodraco breviceps* |  | ^4^ |
|  | *Chaenocephalus aceratus* | Channichthyidae (Notothenioidei) | ^4,15,29^ |
|  | *Champsocephalus gunnari* |  | ^15,29^ |
|  | *Channichthys rhinoceratus* |  | ^29,41^ |
|  | *Chionodraco hamatus* |  | ^42–44^ |
|  | *Chionodraco rastrospinosus* |  | ^4^ |
|  | *Cryodraco antarcticus* |  | ^4,38,42^ |
|  | *Cygnodraco mawsoni* |  | ^36,42^ |
|  | *Pseudochaenichthys georgianus* |  | ^15,29,45^ |
|  | *Macrourus carinatus* | Macrouridae (Gadiformes) | ^46^ |
|  | *Upeneichthys lineatus* | Mullidae (Perciformes) | ^42^ |
|  | *Muraenolepis microps* | Muraenolepididae (Gadiformes) | ^4^ |
|  | *Dissostichus eleginoides* | Nototheniidae | ^3,4,29,30,47,48^ |
|  | *Gobionotothen gibberifrons* |  | ^4,29^ |
|  | *Lepidonotothen squamifrons* |  | ^3,4,29,30^ |
|  | *Lindbergichthys mizops* |  | ^42^ |
|  | *Notothenia coriiceps* |  | ^15,43,44,49,50^ |
|  | *Notothenia rossii* |  | ^3,4,15,29,30^ |
|  | *Nototheniops larseni* |  | ^4^ |
|  | *Nototheniops nudifrons* |  | ^4,14^ |
|  | *Patagonotothen ramsayi* |  | ^29^ |
|  | *Trematomus bernacchii* |  | ^14,37,42,51^ |
|  | *Trematomus eulepidotus* |  | ^17,39^ |
|  | *Trematomus hansoni* |  | ^4,15,44^ |
|  | *Trematomus lepidorhinus* |  | ^17,42^ |
|  | *Trematomus newnesi* |  | ^14,42,52^ |
|  | *Trematomus nicolai* |  | ^17^ |
|  | *Trematomus pennellii* |  | ^42^ |
|  | *Trematomus scotti* |  | ^17,42^ |
| *Lepidapedon garrardi* | *Artedidraco loennbergi* | Artedidraconidae (Notothenioidei) | ^35,53^ |
|  | *Artedidraco shackletoni* |  | ^42^ |
|  | *Artedidraco skottsbergi* |  | ^42^ |
|  | *Bathydraco marri* | Bathydraconidae (Notothenioidei) | ^40,53^ |
|  | *Parachaenichthys charcoti* |  | ^4,54^ |
|  | *Prionodraco evansii* |  | ^40,42,53^ |
|  | *Chaenocephalus aceratus* | Channichthyidae (Notothenioidei) | ^15^ |
|  | *Chionodraco hamatus* |  | ^42^ |
|  | *Chionodraco rastrospinosus* |  | ^4^ |
|  | *Cryodraco antarcticus* |  | ^42^ |
|  | *Dissostichus eleginoides* | Nototheniidae | ^4^ |
|  | *Gobionotothen gibberifrons* |  | ^4,15,19^ |
|  | *Notothenia coriiceps* |  | ^15,43,44,49,50^ |
|  | *Notothenia rossii* |  | ^4,15^ |
|  | *Nototheniops larseni* |  | ^4,6^ |
|  | *Nototheniops nudifrons* |  | ^14,17^ |
|  | *Patagonotothen guntheri* |  | ^4^ |
|  | *Trematomus bernacchii* |  | ^14,42,51^ |
|  | *Trematomus eulepidotus* |  | ^17,53^ |
|  | *Trematomus hansoni* |  | ^17,43,44,53^ |
|  | *Trematomus lepidorhinus* |  | ^17,53^ |
|  | *Trematomus nicolai* |  | ^17,53^ |
|  | *Trematomus pennellii* |  | ^42^ |
|  | *Trematomus scotti* |  | ^17,53^ |
|  | *Tremotomus loennbergi* |  | ^17,53^ |
| *Neolebouria antarctica* | *Gerlachea australis* | Bathydraconidae (Notothenioidei) | ^55^ |
|  | *Gymnodraco acuticeps* |  | ^55^ |
|  | *Parachaenichthys charcoti* |  | ^4,7,54^ |
|  | *Psilodraco breviceps* |  | ^4,7^ |
|  | *Chaenocephalus aceratus* | Channichthyidae (Notothenioidei) | ^4,5,7,15,56^ |
|  | *Chaenodraco wilsoni* |  | ^45^ |
|  | *Champsocephalus gunnari* |  | ^4,7,38^ |
|  | *Chionodraco rastrospinosus* |  | ^4,7^ |
|  | *Cryodraco antarcticus* |  | ^4,7,38^ |
|  | *Neopagetopsis ionah* |  | ^7,38^ |
|  | *Pseudochaenichthys georgianus* |  | ^4,7,45^ |
|  | *Paraliparis meganchus* | Liparidae (Scorpaeniformes) | ^7^ |
|  | *Dissostichus eleginoides* | Nototheniidae | ^7,47,48^ |
|  | *Dissostichus mawsoni* |  | ^7^ |
|  | *Lepidonotothen squamifrons* |  | ^4,5,7^ |
|  | *Notothenia coriiceps* |  | ^7,50^ |
|  | *Notothenia rossii* |  | ^4,7^ |
|  | *Nototheniops larseni* |  | ^4–7^ |
|  | *Nototheniops nudifrons* |  | ^5,7,14^ |
|  | *Trematomus bernacchii* |  | ^36^ |
|  | *Trematomus eulepidotus* |  | ^5,7,14^ |
|  | *Trematomus hansoni* |  | ^7^ |
|  | *Trematomus newnesi* |  | ^7,14^ |
| *Echinorhynchus petrotschenkoi* | *Chaenocephalus aceratus* | Channichthyidae (Notothenioidei) | ^9^ |
|  | *Cryodraco antarcticus* |  | ^9^ |
|  | *Macrourus whitsoni* | Macrouridae (Gadiformes) | ^57^ |
|  | *Muraenolepis microps* | Muraenolepididae (Gadiformes) | ^5,9^ |
|  | *Dissostichus eleginoides* | Nototheniidae | ^9,48,58^ |
|  | *Notothenia coriiceps* |  | ^49,50^ |
|  | *Nototheniops nybelini* |  | ^9^ |
| *Corynosoma bullosum* | *Artedidraco loennbergi* | Artedidraconidae (Notothenioidei) | ^57^ |
|  | *Artedidraco mirus* |  | ^9^ |
|  | *Artedidraco orianae* |  | ^57^ |
|  | *Pogonophryne permitini* |  | ^57^ |
|  | *Gymnodraco acuticeps* | Bathydraconidae (Notothenioidei) | ^55^ |
|  | *Parachaenichthys charcoti* |  | ^5,9,24,54,55^ |
|  | *Parachaenichthys georgianus* |  | ^9,59^ |
|  | *Prionodraco evansii* |  | ^57^ |
|  | *Racovitzia glacialis* |  | ^57^ |
|  | *Chaenocephalus aceratus* | Channichthyidae (Notothenioidei) | ^5,9,24,60^ |
|  | *Champsocephalus esox* |  | ^61,62^ |
|  |  |  |  |
|  | *Chionodraco rastrospinosus* |  | ^24^ |
|  | *Cryodraco antarcticus* |  | ^9,24^ |
|  | *Pseudochaenichthys georgianus* |  | ^45^ |
|  | *Harpagifer antarcticus* | Harpagiferidae (Notothenioidei) | ^14^ |
|  | *Macrourus whitsoni* | Macrouridae (Gadiformes) | ^57,63^ |
|  | *Muraenolepis microps* | Muraenolepididae (Gadiformes) | ^5,9^ |
|  | *Dissostichus eleginoides* | Nototheniidae | ^9,24,47,48,64^ |
|  | *Dissostichus mawsoni* |  | ^9,58^ |
|  | *Gobionotothen gibberifrons* |  | ^5,60^ |
|  | *Lepidonotothen squamifrons* |  | ^5,9^ |
|  | *Notothenia coriiceps* |  | ^24,50,60^ |
|  | *Notothenia gibberifrons* |  | ^9,24^ |
|  | *Notothenia rossii* |  | ^9,24^ |
|  | *Nototheniops larseni* |  | ^5,6,9^ |
|  | *Nototheniops nudifrons* |  | ^5,6,9,14,24^ |
|  | *Nototheniops nybelini* |  | ^9^ |
|  | *Patagonotothen guntheri* |  | ^9^ |
|  | *Trematomus bernacchii* |  | ^6,14,24^ |
|  | *Trematomus eulepidotus* |  | ^5,57^ |
|  | *Trematomus hansoni* |  | ^24,57,59^ |
|  | *Trematomus lepidorhinus* |  | ^57^ |
|  | *Trematomus loennbergi* |  | ^57^ |
|  | *Trematomus pennellii* |  | ^57^ |
|  | *Trematomus scotti* |  | ^57^ |
|  | *Lycodichthys antarcticus* | Zoarcidae (Perciformes) | ^57^ |
| *Contracaecum osculatum (s.l.)* | *Gerlachea australis* | Bathydraconidae (Notothenioidei) | ^55,65^ |
|  | *Gymnodraco acuticeps* |  | ^55^ |
|  | *Parachaenichthys charcoti* |  | ^5,55^ |
|  | *Racovitzia glacialis* |  | ^55^ |
|  | *Chaenocephalus aceratus* | Channichthyidae (Notothenioidei) | ^5^ |
|  | *Chaenodraco wilsoni* |  | ^45^ |
|  | *Champsocephalus gunnari* |  | ^45^ |
|  | *Chionodraco myersi* |  | ^65^ |
|  | *Cryodraco antarcticus* |  | ^65^ |
|  | *Neopagetopsis ionah* |  | ^45^ |
|  | *Pagetopsis macroptera* |  | ^45^ |
|  | *Pseudochaenichthys georgianus* |  | ^45^ |
|  | *Muraenolepis microps* | Muraenolepididae (Gadiformes) | ^5^ |
|  | *Dissostichus eleginoides* | Nototheniidae | ^47^ |
|  | *Gobionotothen gibberifrons* |  | ^5^ |
|  | *Lepidonotothen squamifrons* |  | ^5^ |
|  | *Notothenia coriiceps* |  | ^49^ |
|  | *Nototheniops larseni* |  | ^5^ |
|  | *Nototheniops nudifrons* |  | ^5^ |
|  | *Pleuragramma antarctica* |  | ^65^ |
|  | *Trematomus eulepidotus* |  | ^5^ |
|  | *Trematomus newnesi* |  | ^52^ |
|  | *Trematomus scotti* |  | ^65^ |
| *Pseudoterranova decipiens (s.l.)* | *Acanthodraco dewitti* | Artedidraconidae (Notothenioidei) | ^25^ |
|  | *Artedidraco orianae* |  | ^10^ |
|  | *Artedidraco skottsbergi* |  | ^10^ |
|  | *Dolloidraco largedorsalis* |  | ^10^ |
|  | *Pogonophryne marmorata* |  | ^25^ |
|  | *Gymnodraco acuticeps* | Bathydraconidae (Notothenioidei) | ^55^ |
|  | *Parachaenichthys charcoti* |  | ^10,54,55^ |
|  | *Racovitzia glacialis* |  | ^55^ |
|  | *Chaenocephalus aceratus* | Channichthyidae (Notothenioidei) | ^5,10,25,29^ |
|  | *Chaenodraco wilsoni* |  | ^25^ |
|  | *Champsocephalus gunnari* |  | ^25^ |
|  | *Chionodraco hamatus* |  | ^10,43^ |
|  | *Chionodraco myersi* |  | ^10^ |
|  | *Chionodraco rastrospinosus* |  | ^10,25^ |
|  | *Cryodraco antarcticus* |  | ^10,25^ |
|  | *Cygnodraco mawsoni* |  | ^65^ |
|  | *Pseudochaenichthys georgianus* |  | ^10,29,45^ |
|  | *Paradiplospinus gracilis* | Gempylidae (Perciformes) | ^10^ |
|  | *Harpagifer antarcticus* | Harpagiferidae (Notothenioidei) | ^14,66^ |
|  | *Macrourus whitsoni* | Macrouridae (Gadiformes) | ^63^ |
|  | *Muraenolepis microps* | Muraenolepididae (Gadiformes) | ^5,10,25^ |
|  | *Dissostichus eleginoides* | Nototheniidae | ^48^ |
|  | *Dissostichus mawsoni* |  | ^25^ |
|  | *Gobionotothen gibberifrons* |  | ^5,10,25^ |
|  | *Lepidonototen squamifrons* |  | ^25^ |
|  | *Notothenia coriiceps* |  | ^43^ |
|  | *Notothenia rossii* |  | ^3,25,29,30^ |
|  | *Nototheniops larseni* |  | ^5,6,10^ |
|  | *Nototheniops nudifrons* |  | ^6,10,14,25^ |
|  | *Nototheniops nybelini* |  | ^25^ |
|  | *Trematomus bernacchii* |  | ^14,25^ |
|  | *Trematomus eulepidotus* |  | ^5,10,25^ |
|  | *Trematomus hansoni* |  | ^10,25,43^ |
|  | *Trematomus lepidorhinus* |  | ^10,25^ |
|  | *Trematomus loennbergi* |  | ^10^ |
|  | *Trematomus newnesi* |  | ^14,25^ |
|  | *Trematomus scotti* |  | ^10,65^ |
|  | *Lycenchelys aratrirostris* | Zoarcidae (Perciformes) | ^25^ |
| *Ascarophis nototheniae* | *Gerlachea australis* | Bathydraconidae (Notothenioidei) | ^55^ |
|  | *Gymnodraco acuticeps* |  | ^36^ |
|  | *Parachaenichthys charcoti* |  | ^5,54,55^ |
|  | *Racovitzia glacialis* |  | ^55^ |
|  | *Cryodraco antarcticus* | Channichthyidae (Notothenioidei) | ^67^ |
|  | *Pseudochaenichthys georgianus* |  | ^45^ |
|  | *Harpagifer antarcticus* | Harpagiferidae (Notothenioidei) | ^66^ |
|  | *Macroururs carinatus* | Macrouridae (Gadiformes) | ^46^ |
|  | *Dissostichus eleginoides* | Nototheniidae | ^48^ |
|  | *Gobionotothen gibberifrons* |  | ^5^ |
|  | *Lepidonotothen squamifrons* |  | ^3,5,29^ |
|  | *Notothenia acuta* |  | ^29^ |
|  | *Notothenia coriiceps* |  | ^43,49,50^ |
|  | *Notothenia rossi* |  | ^29^ |
|  | *Nototheniops larseni* |  | ^5^ |
|  | *Nototheniops nudifrons* |  | ^5,14^ |
|  | *Trematomus bernacchii* |  | ^14,43,51^ |
|  | *Trematomus hansoni* |  | ^43^ |
|  | *Trematomus newnesi* |  | ^14^ |

**References**

1. Oğuz, M. C. *et al.* Metazoan parasites of Antarctic fishes. *Turkiye parazitolojii dergisi* (2015) doi:10.5152/tpd.2015.3661.

2. Gibson, D. I., Bray, R. A. & Harris, E. A. Host-Parasite Database of the Natural History Museum, London. http://www.nhm.ac.uk/research-curation/scientific-resources/taxonomy-systematics/host-parasites/ (2005).

3. Parukhin, A. M. Peculiarities of nototheniid fish helminth fauna in subantarctic sector of Indian Ocean. *Vestnik Zoologii* **3**, 6–10 (1986).

4. Zdzitowiecki, K. Occurrence of digeneans in open sea fishes off the South Shetland Islands and South Georgia, and a list of fish digeneans in the Antarctic. *Polish Polar Research* **12**, 55–72 (1991).

5. Palm, H. W., Klimpel, S. & Walter, T. Demersal fish parasite fauna around the South Shetland Islands: high species richness and low host specificity in deep Antarctic waters. *Polar Biol* **30**, 1513–1522 (2007).

6. Ruhl, H. A., Hastings, P. A., Zarubick, L. A., Jensen, R. M. & Zdzitowiecki, K. Fish populations of Port Foster, Deception Island, Antarctica and vicinity. *Deep Sea Research Part II: Topical Studies in Oceanography* **50**, 1843–1858 (2003).

7. Zdzitowiecki, K., Pisano, E. & Vacchi, M. Antarctic representatives of the genus *Neolebouria* Gibson, 1976 (Digenea, Opecoelidae), with description of one new species. *Acta Parasitologica* **38**, 11–14 (1993).

8. Wojciechowska, A. The tetraphyllidean and tetrabothriid cercoids from Antarctic bony fishes. II. Occurrence of cercoids in various fish species. *Acta Parasitologica* **38**, 113–118 (1993).

9. Zdzitowiecki, K. Occurrence of acanthocephalans in fishes of the open sea off the South Shetlands and South Georgia (Antarctic). *Acta Parasitologica Polonica* **35**, 131–141 (1990).

10. Palm, H. W. Ecology of *Pseudoterranova decipiens* (Krabbe, 1878) (Nematoda : Anisakidae) from Antarctic waters. *Parasitol. Res.* **85**, 638–646 (1999).

11. Utevsky, A. Y. An Identification Key to Antarctic Fish leeches (Hirudinea: Piscicolidae). *Ukrainian Antarctic Journal* (2005).

12. García, R. G. G. F., Pradi-García, M. M., Del Valle, M. T. & Rodríguez Diego. New species of hosts and localization for *Pseudobenedenia nototheniae* and *Pseudobenedenoides shorti* (Monogenea: Capsalidae) in Antarctic fishes. *Revista de Salud Animal* **22**, 61–63 (2000).

13. Rokicka, M. Report on species of *Gyrodactylus* Nordmann, 1832, distribution in polar regions. *Polar Science* **3**, 203–206 (2009).

14. Laskowski, Z. & Zdzitowiecki, K. The helminth fauna of some notothenioid fishes collected from the shelf of Argentine Islands, West Antarctica. *Pol. Polar Res.* **26**, 315–324 (2005).

15. Zdzitowiecki, K. Digenetic trematodes in alimentary tracts of fishes of south Georgia and South Shetland Islands (Antarctica). *Acta Ichthyologica et Piscatoria* **9**, 15–30 (1979).

16. Zdzitowiecki, K. Antarctic representatives of the genus *Lecithaster* Lühe, 1901 (Digenea, Hemiuridae), with the description of a new species. *Acta Parasitologica* **37**, 57–63 (1992).

17. Zdzitowiecki, K. Occurrence of digenea in fishes of the family Nototheniidae in the Weddell Sea. *Acta Parasitologica* **47**, 154–158 (2002).

18. Bray, R. A. & Gibson, D. I. The Lepocreadiidae (Digenea) of fishes of the North-East Atlantic - Review of the genera *Opechona* Looss, 1907 and *Prodistomum* Linton, 1910. *Syst. Parasitol.* **15**, 159–202 (1990).

19. Zdzitowiecki, K. *Antarctic digenea parasites of fishes*. (Koeltz Scientific Books, 1997).

20. Zdzitowiecki, K. Antarctic representatives of the genus *Macvicaria* Gibson & Bray, 1982 (Digenea Opecoelidae), with descriptions of two new species. *Syst Parasitol* **16**, 169–179 (1990).

21. Zdzitowiecki, K. Occurrence of digenetic trematodes in fishes off South Shetlands (Antarctic). *Acta Parasitologica Polonica* **33**, 155–167 (1988).

22. Zdzitowiecki, K. & White, M. G. Digenean Trematoda infection of inshore fish at South Georgia. *Antarctic Science* **4**, 51–55 (1992).

23. Zdzitowiecki, K. & Rokosz, B. Prevalence of acanthocephalans in fishes of South Shetland Islands (Antarctic). II. *Aspersentis austrinus* Van Cleave, 1929 and remarks on the validity of *Heteracanthocephalus hureaui* Dollfus, 1965. *Acta Parasitologica Polonica* **30**, 161–171 (1986).

24. Zdzitowiecki, K. Prevalence of acanthocephalans in fishes of South Shetland Islands (Antarctic) III *Metacanthocephalus johnstoni* Zdzitowiecki, 1983, *M. dalmori* Zdzitowiecki, 1983 and notes on other species; general conclusions. *Acta Parasitologica Polonica* **31**, 125–141 (1986).

25. Rokicki, J., Rodjuk, G., Zdzitowiecki, K. & Laskowski, Z. Larval ascaridoid nematodes (Anisakidae) in fish from the South Shetland Islands (Southern Ocean). *Pol. Polar. Res.* **30**, 49–58 (2009).

26. Kovalyova, A. A. & Gaevskaya, A. V. Two species of Monogenea parasites of Antarctic fishes. **56**, 783–786 (1977).

27. Rohde, K., Ho, J.-S., Smales, L. & Williams, R. Parasites of Antarctic fishes: Monogenea, Copepoda and Acanthocephala. *Mar. Freshwater Res.* **49**, 121–125 (1998).

28. Rohde, K., Hayward, C. & Heap, M. Aspects of the ecology of metazoan ectoparasites of marine fishes. *International Journal for Parasitology* **25**, 945–970 (1995).

29. Parukhin, A. M. & Lyadov, V. N. Parasitofauna of Notothenioidei from waters of the Atlantic and Indian Oceans. *Vestnik Zoologii* **3**, 90–94 (1981).

30. Parukhin, A. M. & Lyadov, V. N. Helminth fauna of food Nototheniidae fishes from Kerguelen Subregion region. *Ekologiya Morya* **10**, 49–56 (1982).

31. Bray, R. A. & Zdzitowiecki, K. *Boreascotia megavesicula* n. g., n. sp. (Digenea: Hemiuridae: Lecithochiriinae) in the nototheniid fish *Lepidonotothen macrophthalma* (Norman) from the sub-Antarctic Atlantic. *Syst Parasitol* **46**, 29–32 (2000).

32. Zdzitowiecki, K. Little known and new Antarctic Digenea species of the genera *Neolepidapedon* and *Lepidapedon* (Lepocreadiidae). *Acta Parasitologica Polonica* **35**, 19–30 (1990).

33. Zdzitowiecki, K. Digeneans of the families Opecoelidae and Lepocreadiidae, parasites of *Lepidonotothen macrophthalma* from the North Scotia Ridge, and remarks on the discrimination of *Neolepidapedon magnatestis* and *N. trematomi*. *Acta Parasitologica* **44**, 233–240 (1999).

34. Wojciechowska, A., Pisano, E. & Zdzitowiecki, K. Cestodes in fishes at the Heard Island (Subatarctic). *Pol. Polar Res.* **16**, 205–212 (1995).

35. Zdzitowiecki, K. Occurrence of digenea in fishes of the family Artedidraconidae in the Weddell Sea and other areas of Antarctica. *Acta Parasitologica* **47**, 306–309 (2002).

36. Laskowski, Z., Rocka, A., Ghigliotti, L. & Pisano, E. New data on the occurrence of internal parasitic worms in the *Gymnodraco acuticeps* and *Cygnodraco mawsoni* [Bathydraconidae] fish in the Ross Sea, Antarctica. *Polish Polar Research* **26**, 37–40 (2005).

37. Szidat, L. & Graefe, G. *Estudios sobre la fauna de parasitos de peces antarticos II-Los parasitos de* Parachaenichthys charcoti. (Servicio de Hidrografia Naval, 1967).

38. Zdzitowiecki, K. Occurrence of digenea in fishes of the family Channichthyidae in the Weddell Sea and other sub-continental areas of the Antarctica. *Acta Parasitologica* **47**, 159–162 (2002).

39. Kock, K.-H., Schneppenheim, R. & Siegel, V. A contribution to the fish fauna of the Weddell sea. *Archiv für Fischereiwissenschaft* **34**, 103–120 (1984).

40. Zdzitowiecki, K. Occurrence of digenea in fishes of the family Bathydraconidae in the Weddell Sea and other areas of Antarctica. *Acta Parasitologica* **47**, 310–313 (2002).

41. Lyadov, V. N., Parukhin, A. M. & Mironova, A. V. Helminth Fauna of the Family Chaenichthidae from the Region of Kerguelen Subregion Islands. *Zoologicheskii Zhurnal* **60**, 142–144 (1981).

42. Prudhoe, S. & Bray, R. A. Digenetic trematodes from fishes. *B.A.N.Z. Antarctic Res Expedition, Reports B (Zoology and Botany)* **8**, 195–225 (1973).

43. Zdzitowiecki, K., Rocka, A., Pisano, E. & Ozouf-Costaz, C. A list of fish parasitic worms collected off Adelie Land (Antarctica). *Acta Parasitologica* **43**, 71–74 (1998).

44. Zdzitowiecki, K. New data on the occuurence of fish endoparasitic worms off Adelie Land, Antarctica. *Pol. Polar Res.* **22**, 159–165 (2001).

45. Kuhn, T. *et al.* Lighten up the dark: metazoan parasites as indicators for the ecology of Antarctic crocodile icefish (Channichthyidae) from the north-west Antarctic Peninsula. *PeerJ* **6**, e4638 (2018).

46. Gaevskaya, A. V. & Rodyuk, G. N. Ecological characteristics of the parasite fauna of *Macrourus carinatus* Gunther. *Biologicheskie Nauki* **2**, 21–25 (1988).

47. Gaevskaya, A. V., Kovalyova, A. A. & Parukhin, A. M. Peculiarities and formation of parasitofauna of the Patagonian toothfish *Dissostichus eleginoides*. *Biologiya Morya* **4**, 23–28 (1990).

48. Brickle, P., MacKenzie, K. & Pike, A. Parasites of the Patagonian toothfish, *Dissostichus eleginoides* Smitt 1898, in different parts of the Subantarctic. *Polar Biol.* **288**, 633–671 (2005).

49. Palm, H. W., Reimann, N., Spindler, M. & Plötz, J. The role of the rock cod *Notothenia coriiceps* Richardson, 1844 in the life-cycle of Antarctic parasites. *Polar Biol* **19**, 399–406 (1998).

50. Zdzitowiecki, K. & Laskowski, Z. Helminths of an Antarctic fish, *Notothenia coriiceps*, from the Vernadsky Station (Western Antarctica) in comparison with Admiralty Bay (South Shetland Islands). *Helminthologia* **41**, 201–207 (2004).

51. Moser, M. & Cowen, R. K. The effects of periodic eutrophication on parasitism and stock identification of *Trematomus bernacchii* (Pisces: Nototheniidae) in McMurdo Sound, Antarctica. *The Journal of Parasitology* **77**, 551–556 (1991).

52. Laskowski, Z., A, R., K, Z. & C, O.-C. Occurrence of endoparasitic worms in dusky notothen, *Trematomus newnesi* [Actinopterygii Nototheniidae], at Adelie Land, Antarctica. *Polish Polar Research* **1**, (2007).

53. Zdzitowiecki, K. & Cielecka, D. Digenea of fishes of the Weddell Sea. III. The Lepocreadiidae (genera *Neolepidapedon* and *Lepidapedon*), parasites of Notothenioidea. *Acta Parasitologica* **42**, 84–91 (1997).

54. Zdzitowiecki, K. Occurrence of endoparasitic worms in fish, *Parachaenichthys charcoti* (Bathydraconidae), off the South Shetland Islands (Antarctica). *Acta Parasitologica* **46**, 18–23 (2001).

55. Münster, J., Kochmann, J., Grigat, J., Klimpel, S. & Kuhn, T. Parasite fauna of the Antarctic dragonfish *Parachaenichthys charcoti* (Perciformes: Bathydraconidae) and closely related Bathydraconidae from the Antarctic Peninsula, Southern Ocean. *Parasites Vectors* **10**, 235 (2017).

56. Zdzitowiecki, K. Digenea of fishes of the Weddell Sea. IV. Three Opecoelid species of the genera *Neolebouria, Helicometra*, and *Stenakron*. *Acta Parasitologica* **42**, 138–143 (1997).

57. Zdzitowiecki, K. Acanthocephala in fish in the Weddell Sea (Antarctic). *Acta Parasitologica* **41**, 199–203 (1996).

58. Gordeev, I. I. & Sokolov, S. G. Parasites of the Antarctic toothfish (Dissostichus mawsoni Norman, 1937) (Perciformes, Nototheniidae) in the Pacific sector of the Antarctic. *Polar Research* **35**, 29364 (2016).

59. Zdzitowiecki, K. Acanthocephalans of marine fishes in the regions of South Georgia and South Orkneys (Antarctic). *Acta Parasitologica* **31**, 211–217 (1987).

60. Zdzitowiecki, K. & White, M. G. Acanthocephalan infection of inshore fishes at the South Orkney Islands. *Antarctic Science* **8**, 273–276 (1996).

61. Laskowski, Z., Jeżewski, W. & Zdzitowiecki, K. Cystacanths of Acanthocephala in notothenioid fish from the Beagle Channel (sub-Antarctica). *Syst Parasitol* **70**, 107–117 (2008).

62. Laskowski, Z. & Zdzitowiecki, K. Occurrence of Acanthocephalans in Notothenioid Fishes in the Beagle Channel (Magellanic Sub-Region, Sub-Antarctic). *Pol. Polar Res.* **30**, 179–186 (2009).

63. Münster, J., Kochmann, J., Klimpel, S., Klapper, R. & Kuhn, T. Parasite fauna of Antarctic *Macrourus whitsoni* (Gadiformes: Macrouridae) in comparison with closely related macrourids. *Parasites & Vectors* **9**, 403 (2016).

64. Brickle, P., MacKenzie, K. & Pike, A. Variations in the parasite fauna of the Patagonian toothfish (*Dissostichus eleginoides* Smitt, 1898), with length, season and depth of habitat around the Falkland Islands. *J Parasitol* **92**, 282–291 (2006).

65. Klöser, H., Plötz, J., Palm, H., Bartsch, A. & Hubold, G. Adjustment of anisakid nematode life cycles to the high Antarctic food web as shown by &lt;span class=&quot;italic&quot;&gt;Contracaecum radiatum&lt;/span&gt; and &lt;span class=&quot;italic&quot;&gt;C. osculatum&lt;/span&gt; in the Weddell Sea. *Antarctic Science* (1992).

66. Zdzitowiecki, K. & Zadrozny, T. Endoparasitic worms of *Harpagifer antarcticus* off the South Shetland Islands (Antarctic). *Acta Parasitologica* **44**, 125–130 (1999).

67. Rocka, A. Biometrical variability and occurrence of *Ascarophis nototheniae* (Nematoda, Cystidicolidae), a parasitic nematode of Antarctic and subantarctic fishes. *Acta Parasitologica* **44**, 188–192 (1999).

**Data S1:** Sequences of Nematoda subsamples from *Nototheniops larseni*, *N. nudifrons* and *Lepidonotothen squamifrons* in .fasta format.

>ant-Nlar-1.521.1_NC5

TCATTATCGAGCGAATCCAAAACGAANAAGTCTCCCAACGTGCATACCATCCATTTGCATGTTGTTGTGAGCCGCATAGAAACTCATACACGCGTGGTGGCAGCCGTCTGCTGTGCTTTATCGTGCAGACAATGGCTTATGAGTGGCTGTGTGATTGTTGAACAACGGTGACCAATTTGGCGTCTACGCCGTATCTAGCTTCCGCCTGGACCGTCGGTAGCGATGAAAGATGCGGAGGAAGTTCCTCTGTTTTGGTTTCAACGCTAACGCAGAGTTGAGCAGACTTAATGAGCCACGCTTGGTGGCCGCCAAAACCCAAAACACAACCAGTCTATTTTAACGTTTGTTGATATGTTAATGTACAAATCTTGGCGGTGGATCACTCGGTTCGTGGATCGATGAAGAACGCAGCCAGCTGCGATAAATAGTGCGAATTGCAGACACATTGAGCACTAAGAATTCGAACGCACATTGCGCTATCGGGTTCATTCCCGATGGCACGTCTGGCTGAGGGTCGAATTGTAGTAAACTGTCTTCAAGTACTTTTTATGGTCGTGAAGTATTCGGCAAGCAGTTGTCGGATAGTTGTTTTTGTTGATCGTCCGTTCGTTCGGTCGGTCCAGAGCAATATTCTGAGGCTCCTTGCTTAGTTGCGTTTTGGTAGACGTTAACACCCGAACAATATGTGGTGGTGATATTTGGNNNNRNTGGCGAGAATCATNGCCGCTTCAATGGNGGCAGCAACCAGCATACGCTAATGACAGTTGGTTGATTGAAGCCAGGGCAACGGAGTGATGTGTGGCGATCATTTAACGTTTGTATTTGACCTCAGCTCAGTCGTGATTACCCGCTGAATTTAAGCATATAATTAAGCGGAGAAAAAAAAAAYYAAAAAAN

>ant-Nlar-1.522.1_NC5

TCTCCCAACGTGCATACCATCCATTTGCATGTTGTTGTGAGCCGCATAGAAACTCATACACGCGTGGTGGCAGCCGTCTGCTGTGCTTTATCGTGCAGACAATGGCTTATGAGTGGCTGTGTGATTGTTGAACAACGGTGACCAATTTGGCGTCTACGCCGTATCTAGCTTCCGCCTGGACCGTCGGTAGCGATGAAAGATGCGGAGGAAGTTCCTCTGTTTTGGTTTCAACGCTAACGCAGAGTTGAGCAGACTTAATGAGCCACGCTTGGTGGCCGCCAAAACCCAAAACACAACCAGTCTATTTTAACGTTTGTTGATATGTTAATGTACAAATCTTGGCGGTGGATCACTCGGTTCGTGGATCGATGAAGAACGCAGCCAGCTGCGATAAATAGTGCGAATTGCAGACACATTGAGCACTAAGAATTCGAACGCACATTGCGCTATCGGGTTCATTCCCGATGGCACGTCTGGCTGAGGGTCGAATTGTAGTAAACTGTCTTCAAGTACTTTTTATGGTCGTGAAGTATTCGGCAAGCAGTTGTCGGATAGTTGTTTTTGTTGATCGTCCGTTCGTTCGGTCGGTCCAGAGCAATATTCTGAGGCTCCTTGCTTAGTTGCGTTTTGGTAGACGTTAACACCCGAACAATATGTGGTGGTGATATTGGTGATGNNNASAA

>ant-Nlar-10.555.1_NC5

TCTCCCAACGTGCATACCATCCATTTGCATGTTGTTGTGAGCCGCATAGAAACTCATACACGCGTGGTGGCAGCCGTCTGCTGTGCTTTATCGTGCAGACAATGGCTTATGAGTGGCTGTGTGATTGTTGAACAACGGTGACCAATTTGGCGTCTACGCCGTATCTAGCTTCCGCCTGGACCGTCGGTAGCGATGAAAGATGCGGAGGAAGTTCCTCTGTTTTGGTTTCAACGCTAACGCAGAGTTGAGCAGACTTAATGAGCCACGCTTGGTGGCCGCCAAAACCCAAAACACAACCAGTCTATTTTAACGTTTGTTGATATGTTAATGTACAAATCTTGGCGGTGGATCACTCGGTTCGTGGATCGATGAAGAACGCAGCCAGCTGCGATAAATAGTGCGAATTGCAGACACATTGAGCACTAAGAATTCGAACGCACATTGCGCTATCGGGTTCATTCCCGATGGCACGTCTGGCTGAGGGTCGAATTGTAGTAAACTGTCTTCAAGTACTTTTTATGGTCGTGAAGTATTCGGCAAGCAGTTGTCGGATAGTTGTTTTTGTTGATCGTCCGTTCGTTCGGTCGGTCCAGAGCAATATTCTGAGGCTCCTTGCTTAGTTGCGTTTTGGTAGACGTTAACACCCGAACAATATGTGGTGGTGATATTGKTGATGSWGAG

>ant-Nlar-14.562.1_NC5

ANTCTCCCAACGTGCATACCATCCATTTGCATGTTGTTGTGAGCCGCATAGAAACTCATACACGCGTGGTGGCAGCCGTCTGCTGTGCTTTATCGTGCAGACAATGGCTTATGAGTGGCTGTGTGATTGTTGAACAACGGTGACCAATTTGGCGTCTACGCCGTATCTAGCTTCCGCCTGGACCGTCGGTAGCGATGAAAGATGCGGAGGAAGTTCCTCTGTTTTGGTTTCAACGCTAACGCAGAGTTGAGCAGACTTAATGAGCCACGCTTGGTGGCCGCCAAAACCCAAAACACAACCAGTCTATTTTAACGTTTGTTGATATGTTAATGTACAAATCTTGGCGGTGGATCACTCGGTTCGTGGATCGATGAAGAACGCAGCCAGCTGCGATAAATAGTGCGAATTGCAGACACATTGAGCACTAAGAATTCGAACGCACATTGCGCTATCGGGTTCATTCCCGATGGCACGTCTGGCTGAGGGTCGAATTGTAGTAAACTGTCTTCAAGTACTTTTTATGGTCGTGAAGTATTCGGCAAGCAGTTGTCGGATAGTTGTTTTTGTTGATCGNCCGWYCGNTCG

>ant-Nlar-15.568.1_NC5

TCTCCCAACGTGCATACCATCCATTTGCATGTTGTTGTGAGCCGCATAGAAACTCATACACGCGTGGTGGCAGCCGTCTGCTGTGCTTTATCGTGCAGACAATGGCTTATGAGTGGCTGTGTGATTGTTGAACAACGGTGACCAATTTGGCGTCTACGCCGTATCTAGCTTCCGCCTGGACCGTCGGTAGCGATGAAAGATGCGGAGGAAGTTCCTCTGTTTTGGTTTCAACGCTAACGCAGAGTTGAGCAGACTTAATGAGCCACGCTTGGTGGCCGCCAAAACCCAAAACACAACCAGTCTATTTTAACGTTTGTTGATATGTTAATGTACAAATCTTGGCGGTGGATCACTCGGTTCGTGGATCGATGAAGAACGCAGCCAGCTGCGATAAATAGTGCGAATTGCAGACACATTGAGCACTAAGAATTCGAACGCACATTGCGCTATCGGGTTCATTCCCGATGGCACGTCTGGCTGAGGGTCGAATTGTAGTAAACTGTCTTCAAGTACTTTTTATGGTCGTGAAGTATTCGGCAAGCAGTTGTCGGATAGTTGTTTTTGTTGATCGNCCGTTCGTTCGGTCGGTCCAGAGYAATATTCTGAGGCTCCTTGCTTAGTTGCGTTTTGGTAGACGTTAACACCCGAACAATATGWGKTGNNG

>ant-Nlar-18.576.1_NC5

NNTCTCCCAACGTGCATACCATCCATTTGCATGTTGTTGTGAGCCGCATAGAAACTCATACACGCGTGGTGGCAGCCGTCTGCTGTGCTTTATCGTGCAGACAATGGCTTATGAGTGGCTGTGTGATTGTTGAACAACGGTGACCAATTTGGCGTCTACGCCGTATCTAGCTTCCGCCTGGACCGTCGGTAGCGATGAAAGATGCGGAGGAAGTTCCTCTGTTTTGGTTTCAACGCTAACGCAGAGTTGAGCAGACTTAATGAGCCACGCTTGGTGGCCGCCAAAACCCAAAACACAACCAGTCTATTTTAACGTTTGTTGATATGTTAATGTACAAATCTTGGCGGTGGATCACTCGGTTCGTGGATCGATGAAGAACGCAGCCAGCTGCGATAAATAGTGCGAATTGCAGACACATTGAGCACTAAGAATTCGAACGCACATTGCGCTATCGGGTTCATTCCCGATGGCACGTCTGGCTGAGGGTCGAATTGTAGTAAACTGTCTTCAAGTACTTTTTATGGTCGTGAAGTATTCGGCAAGCAGTTGTCGGATAGTTGTTTTTGTTGATCGTCCGTTCGTTCGGTCGGTCCAGAGCAATATTCTGAGGCTCCTTGCTTAGTTGCGTTTTGGTAGACGTTAACACCCGAACAATATGTGGTGGTGATATTGGTGATGSCGAGAANCAN

>ant-Nlar-2.526.1_NC5

NTCTCCCAACGTGCATACCATCCATTTGCATGTTGTTGTGAGCCGCATAGAAACTCATACACGCGTGGTGGCAGCCGTCTGCTGTGCTTTATCGTGCAGACAATGGCTTATGAGTGGCTGTGTGATTGTTGAACAACGGTGACCAATTTGGCGTCTACGCCGTATCTAGCTTCCGCCTGGACCGTCGGTAGCGATGAAAGATGCGGAGGAAGTTCCTCTGTTTTGGTTTCAACGCTAACGCAGAGTTGAGCAGACTTAATGAGCCACGCTTGGTGGCCGCCAAAACCCAAAACACAACCAGTCTATTTTAACGTTTGTTGATATGTTAATGTACAAATCTTGGCGGTGGATCACTCGGTTCGTGGATCGATGAAGAACGCAGCCAGCTGCGATAAATAGTGCGAATTGCAGACACATTGAGCACTAAGAATTCGAACGCACATTGCGCTATCGGGTTCATTCCCGATGGCACGTCTGGCTGAGGGTCGAATTGTAGTAAACTGTCTTCAAGTACTTTTTATGGTCGTGAAGTATTCGGCAAGCAGTTGTCGGATAGTTGTTTTTGTTGATCGTCCGTTCGTTCGGTCGGTCCAGAGCAATATTCTGAGGCTCCTTGCTTAGTTGCGTTTTGGTAGACGTTAACACCCGAACAATATGTGGTGGTGATAWTTGGGTGRAGGNCRRAGAATCATGCCGNCTTCAATGGGNGCAGCAACCAGCATACGCTAATGACAGTTGGTTGATTGAAGCCAGGGCAACGGAGTGATGTGTGGCGATCATTTAACGTTTGTATTTGACCTCAGCTCAGTCGTGATTACCCGCTGAATTTAAGCATATAATTAAGCGAGAANN

>ant-Nlar-2.527.1_NC5

TTATCGAGCGAATCCAAAACGAAAAAGTCTCCCAACGTGCATACCATCCATTTGCATGTTGTTGTGAGCCGCATAGAAACTCATACACGCGTGGTGGCAGCCGTCTGCTGTGCTTTATCGTGCAGACAATGGCTTATGAGTGGCTGTGTGATTGTTGAACAACGGTGACCAATTTGGCGTCTACGCCGTATCTAGCTTCCGCCTGGACCGTCGGTAGCGATGAAAGATGCGGAGGAAGTTCCTCTGTTTTGGTTTCAACGCTAACGCAGAGTTGAGCAGACTTAATGAGCCACGCTTGGTGGCCGCCAAAACCCAAAACACAACCAGTCTATTTTAACGTTTGTTGATATGTTAATGTACAAATCTTGGCGGTGGATCACTCGGTTCGTGGATCGATGAAGAACGCAGCCAGCTGCGATAAATAGTGCGAATTGCAGACACATTGAGCACTAAGAATTCGAACGCACATTGCGCTATCGGGTTCATTCCCGATGGCACGTCTGGCTGAGGGTCGAATTGTAGTAAACTGTCTTCAAGTACTTTTTATGGTCGTGAAGTATTCGGCAAGCAGTTGTCGGATAGTTGTTTTTGTTGATCGTCCGTTCGTTCGGTCGGTCCAGAGCAATATTCTGAGGCTCCTTGCTTAGTTGCGTTTTGGTAGACGTTAACACCCGAACAATATGTGGTGGTGATAWTTGGNTGATGNNCNAGAATCATGCCGCTTCAATGGGGCAGCAACCAGCATACGCTAATGACAGTTGGTTGATTGAAGCCAGGGCAACGGAGTGATGTGTGGCGATCATTTAACGTTTGTATTTGACCTCAGCTCAGTCGTGATTACCCGCTGAATTTAAGCATATAATTAAGCGGAGGAAAANAAAACTAAAAANNN

>ant-Nlar-20.584.1_NC5

ATACCATCCATTTGCATGTTGTTGTGAGCCGCATAGAAACTCATACACGCGTGGTGGCAGCCGTCTGCTGTGCTTTATCGTGCAGACAATGGCTTATGAGTGGCTGTGTGATTGTTGAACAACGGTGACCAATTTGGCGTCTACGCCGTATCTAGCTTCCGCCTGGACCGTCGGTAGCGATGAAAGATGCGGAGGAAGTTCCTCTGTTTTGGTTTCAACGCTAACGCAGAGTTGAGCAGACTTAATGAGCCACGCTTGGTGGCCGCCAAAACCCAAAACACAACCAGTCTATTTTAACGTTTGTTGATATGTTAATGTACAAATCTTGGCGGTGGATCACTCGGTTCGTGGATCGATGAAGAACGCAGCCAGCTGCGATAAATAGTGCGAATTGCAGACACATTGAGCACTAAGAATTCGAACGCACATTGCGCTATCGGGTTCATTCCCGATGGCACGTCTGGCTGAGGGTCGAATTGTAGTAAACTGTCTTCAAGTACTTTTTATGGTCGTGAAGTATTCGGCAAGCAGTTGTCGGATAGTTGTTTTTGTTGATCGTCCGTTCGTTCGGTCGGTCCAGAGCAATATTCTGAGGCTCCTTGCTTAGTTGCGTTTTGGTAGACGTTAACACCCGAACAATATGTGGTGGTGAT

>ant-Nlar-21.588.1_NC5

NTCTCCCAACGTGCATACCATCCATTTGCATGTTGTTGTGAGCCGCATAGAAACTCATACACGCGTGGTGGCAGCCGTCTGCTGTGCTTTATCGTGCAGACAATGGCTTATGAGTGGCTGTGTGATTGTTGAACAACGGTGACCAATTTGGCGTCTACGCCGTATCTAGCTTCCGCCTGGACCGTCGGTAGCGATGAAAGATGCGGAGGAAGTTCCTCTGTTTTGGTTTCAACGCTAACGCAGAGTTGAGCAGACTTAATGAGCCACGCTTGGTGGCCGCCAAAACCCAAAACACAACCAGTCTATTTTAACGTTTGTTGATATGTTAATGTACAAATCTTGGCGGTGGATCACTCGGTTCGTGGATCGATGAAGAACGCAGCCAGCTGCGATAAATAGTGCGAATTGCAGACACATTGAGCACTAAGAATTCGAACGCACATTGCGCTATCGGGTTCATTCCCGATGGCACGTCTGGCTGAGGGTCGAATTGTAGTAAACTGTCTTCAAGTACTTTTTATGGTCGTGAAGTATTCGGCAAGCAGTTGTCGGATAGTTGTTTTTGTTGATCGNCCGTTCGTTCGGTCGGTCCAGAGCAATATTCTGAGGYTCCTTGCTTAGYTGCGTTTTGGTAGACGTTAACACCCGAACAAYAYGCGKTGK

>ant-Nlar-22.591.1_NC5

TCTCCCAACGTGCATACCATCCATTTGCATGTTGTTGTGAGCCGCATAGAAACTCATACACGCGTGGTGGCAGCCGTCTGCTGTGCTTTATCGTGCAGACAATGGCTTATGAGTGGCTGTGTGATTGTTGAACAACGGTGACCAATTTGGCGTCTACGCCGTATCTAGCTTCCGCCTGGACCGTCGGTAGCGATGAAAGATGCGGAGGAAGTTCCTCTGTTTTGGTTTCAACGCTAACGCAGAGTTGAGCAGACTTAATGAGCCACGCTTGGTGGCCGCCAAAACCCAAAACACAACCAGTCTATTTTAACGTTTGTTGATATGTTAATGTACAAATCTTGGCGGTGGATCACTCGGTTCGTGGATCGATGAAGAACGCAGCCAGCTGCGATAAATAGTGCGAATTGCAGACACATTGAGCACTAAGAATTCGAACGCACATTGCGCTATCGGGTTCATTCCCGATGGCACGTCTGGCTGAGGGTCGAATTGTAGTAAACTGTCTTCAAGTACTTTTTATGGTCGTGAAGTATTCGGCAAGCAGTTGTCGGATAGTTGTTTTTGTTGATCGTCCGTTCGTTCGGTCGGTCCAGAGCAATATTCTGAGGCTCCTTGCTTAGTTGCGTTTTGGTAGACGTTAACACCCGAACAATATGTGGTGGTGATATTGNTGATGYNGAGA

>ant-Nlar-23.594.1_NC5

TCTCCCAACGTGCATACCATCCATTTGCATGTTGTTGTGAGCCGCATAGAAACTCATACACGCGTGGTGGCAGCCGTCTGCTGTGCTTTATCGTGCAGACAATGGCTTATGAGTGGCTGTGTGATTGTTGAACAACGGTGACCAATTTGGCGTCTACGCCGTATCTAGCTTCCGCCTGGACCGTCGGTAGCGATGAAAGATGCGGAGGAAGTTCCTCTGTTTTGGTTTCAACGCTAACGCAGAGTTGAGCAGACTTAATGAGCCACGCTTGGTGGCCGCCAAAACCCAAAACACAACCAGTCTATTTTAACGTTTGTTGATATGTTAATGTACAAATCTTGGCGGTGGATCACTCGGTTCGTGGATCGATGAAGAACGCAGCCAGCTGCGATAAATAGTGCGAATTGCAGACACATTGAGCACTAAGAATTCGAACGCACATTGCGCTATCGGGTTCATTCCCGATGGCACGTCTGGCTGAGGGTCGAATTGTAGTAAACTGTCTTCAAGTACTTTTTATGGTCGTGAAGTATTCGGCAAGCAGTTGTCGGATAGTTGTTTTTGTTGATCGTCCGTTCGTTCGGTCGGTCCAGAGCAATATTCTGAGGCTCCTTGCTTAGTTGCGTTTTGGTAGACGTTAACACCCGAACAATATGTGGTGGTGATATTGGTGATGGCGAGAATCATGCCGCTTCAATGGNGNAGSMMCSAGCAWACGCTAATGACAGATGGYTGATTGMAGCCAGGKAAAMGKAWTGATGTGTGTCGATCATTTAGCGTWTGTATKTGAC

>ant-Nlar-24.595.1_NC5

TCTCCCAACGTGCATACCATCCATTTGCATGTTGTTGTGAGCCGCATAGAAACTCATACACGCGTGGTGGCAGCCGTCTGCTGTGCTTTATCGTGCAGACAATGGCTTATGAGTGGCTGTGTGATTGTTGAACAACGGTGACCAATTTGGCGTCTACGCCGTATCTAGCTTCCGCCTGGACCGTCGGTAGCGATGAAAGATGCGGAGGAAGTTCCTCTGTTTTGGTTTCAACGCTAACGCAGAGTTGAGCAGACTTAATGAGCCACGCTTGGTGGCCGCCAAAACCCAAAACACAACCAGTCTATTTTAACGTTTGTTGATATGTTAATGTACAAATCTTGGCGGTGGATCACTCGGTTCGTGGATCGATGAAGAACGCAGCCAGCTGCGATAAATAGTGCGAATTGCAGACACATTGAGCACTAAGAATTCGAACGCACATTGCGCTATCGGGTTCATTCCCGATGGCACGTCTGGCTGAGGGTCGAATTGTAGTAAACTGTCTTCAAGTACTTTTTATGGTCGTGAAGTATTCGGCAAGCAGTTGTCGGATAGTTGTTTTTGTTGATCGTCCGTTCGTTCGGTCGGTCCAGAGCAATATTCTGAGGCTCCTTGCTTAGTTGCGTTTTGGTAGACGTTAACACCCGAACAATATGTGGTGNTGATA

>ant-Nlar-26.598.1_NC5

CGTGCATAAATTCCATTTGCGCGTAATCGTGAGCCATGCAGCAAGCCATACACATGTGGTGGCAGCCGTCAGCTGTTTYGTGGCAGACAATGGCTTTGGCTTGCTGTGTGTGTTGAGGGGAAGTGAGTGACCCGATATGCTTCAAAGGCGGGTCGATTGCGCTCATTTTCTCGTTATTCGTAAAAACGGTATCCACTTTGGCGTCTACGCCGTACCTAGCTACTGCCTGGACCGTCGGTAGCGATGAAAGGTGCGGAGAAAGTTCCTCATTTTGAGTTGAGCAGACTTAATGAGCCCGGCAAGAGGGCCGCCAAAACCAAAACACAACCATTTCTTTATTTTTAATAGTTGATCGGTGAATCGGGGTGTTCGTTATGTGACAACATGTGGTTGTCGCTGCGGATGTCTTGGTGAATCGTTAACTACAAATCTTGGCGGTGGATCACTCGGTTCGTGGATCGATGAAGAACGCAGCCAGCTGCGATAAATAGTGCGAATTGCAGACACATTGAGCACTAAAATTTCGAACGCACATTGCGCCATCGGGTTCATTCCCGCTGGCACGTCTGGCTGAGGGTCGAAATGTTCGACACTATCCGCACAATACTTCCTGTGAGCAGGGAAGTGTGTGGTGCATTCGGCAAGCAGTGATTCTTGAGCATTGCTCTCGAAAGCGCTCCTTGCTTAGACGGAACGGTAAATAATTGCTAAATTTTACTCGGTAAGGTGCAATTATAGCCGCGAATATGTTGCTTCTCGATGCGGCTTCCAGTATTTGTTGATTGTCGATAGCGACTGGAAGTATGGCATCGGATGCCTGATAGAGTGATATGTCTTGNTTGNTATG

>ant-Nlar-27.602.1_NC5

ACGTGCATACCATCCATTTGCATGTTGTTGTGAGCCGCATAGAAACTCATACACGCGTGGTGGCAGCCGTCTGCTGTGCTTTATCGTGCAGACAATGGCTTATGAGTGGCTGTGTGATTGTTGAACAACGGTGACCAATTTGGCGTCTACGCCGTATCTAGCTTCCGCCTGGACCGTCGGTAGCGATGAAAGATGCGGAGGAAGTTCCTCTGTTTTGGTTTCAACGCTAACGCAGAGTTGAGCAGACTTAATGAGCCACGCTTGGTGGCCGCCAAAACCCAAAACACAACCAGTCTATTTTAACGTTTGTTGATATGTTAATGTACAAATCTTGGCGGTGGATCACTCGGTTCGTGGATCGATGAANAACGCAGCCAGCTGCGATAAATAGNGCGAATTGCAGACACNTTGAGCACTA

>ant-Nlar-28.605.1_NC5

NTCTCCCAACGTGCATACCATCCATTTGCATGTTGTTGTGAGCCGCATAGAAACTCATACACGCGTGGTGGCAGCCGTCTGCTGTGCTTTATCGTGCAGACAATGGCTTATGAGTGGCTGTGTGATTGTTGAACAACGGTGACCAATTTGGCGTCTACGCCGTATCTAGCTTCCGCCTGGACCGTCGGTAGCGATGAAAGATGCGGAGGAAGTTCCTCTGTTTTGGTTTCAACGCTAACGCAGAGTTGAGCAGACTTAATGAGCCACGCTTGGTGGCCGCCAAAACCCAAAACACAACCAGTCTATTTTAACGTTTGTTGATATGTTAATGTACAAATCTTGGCGGTGGATCACTCGGTTCGTGGATCGATGAAGAACGCAGCCAGCTGCGATAAATAGTGCGAATTGCAGACACATTGAGCACTAAGAATTCGAACGCACATTGCGCTATCGGGTTCATTCCCGATGGCACGTCTGGCTGAGGGTCGAATTGTAGTAAACTGTCTTCAAGTACTTTTTATGGTCGTGAAGTATTCGGCAAGCAGTTGTCGGATAGTTGTTTTTGTTGATCGTCCGTTCGTTCGGTCGGTCCAGAGCAATATTCTGAGGCTCCTTGCTTAGTTGCGTTTTGGTAGACGTTAACACCCGAACAATATGTGGTGGTGATATTGGTGATGSCGAGAANCANGN

>ant-Nlar-29.606.1_NC5

CGTGCATACCATCCATTTGCATGTTGTTGTGAGCCGCATAGAAACTCATACACGCGTGGTGGCAGCCGTCTGCTGTGCTTTATCGTGCAGACAATGGCTTATGAGTGGCTGTGTGATTGTTGAACAACGGTGACCAATTTGGCGTCTACGCCGTATCTAGCTTCCGCCTGGACCGTCGGTAGCGATGAAAGATGCGGAGGAAGTTCCTCTGTTTTGGTTTCAACGCTAACGCAGAGTTGAGCAGACTTAATGAGCCACGCTTGGTGGCCGCCAAAACCCAAAACACAACCAGTCTATTTTAACGTTTGTTGATATGTTAATGTACAAATCTTGGCGGTGGATCACTCGGTTCGTGGATCGATGAAGAACGCAGCCAGCTGCGATAAATAGTGCGAATTGCAGACACATTGAGCACTAAGAATTCGAACGCACATTGCGCTATCGGGTTCATTCCCGATGGCACGTCTGGCTGAGGGTCGAATTGTAGTAAACTGTCTTCAAGTACTTTTTATGGTCGTGAAGTATTCGGCAAGCAGTTGTCGGATAGTTGTTTTTGTTGATCGTCCGTTCGTTCGGTCGGTCCAGAGCAATATTCTGAGGCTCCTTGCTTAGTTG

>ant-Nlar-3.530.1_NC5

CCCAACGTGCATACCATCCATTTGCATGTTGTTGTGAGCCGCATAGAAACTCATACACGCGTGGTGGCAGCCGTCTGCTGTGCTTTATCGTGCAGACAATGGCTTATGAGTGGCTGTGTGATTGTTGAACAACGGTGACCAATTTGGCGTCTACGCCGTATCTAGCTTCCGCCTGGACCGTCGGTAGCGATGAAAGATGCGGAGGAAGTTCCTCTGTTTTGGTTTCAACGCTAACGCAGAGTTGAGCAGACTTAATGAGCCACGCTTGGTGGCCGCCAAAACCCAAAACACAACCAGTCTATTTTAACGTTTGTTGATATGTTAATGTACAAATCTTGGCGGTGGATCACTCGGTTCGTGGATCGATGAAGAACGCAGCCAGCTGCGATAAATAGTGCGAATTGCAGACACATTGAGCACTAAGAATTCGAACGCACATTGCGCTATCGGGTTCATTCCCGATGGCACGTCTGGCTGAGGGTCGAATTGTAGTAAACTGTCTTCAAGTACTTTTTATGGTCGTGAAGTATTCGGCAAGCAGTTGTCGGATAGTTGTTTTTGTTGATCGTCCGTTCGTTCGGTCGGTCCAGAGCAATATTCTGAGGCTCCTTGCTTAGTTGCGTTTTGGTAGACGTTAACACCCGAACAATATGTGGTGNTG

>ant-Nlar-3.531.1_NC5

ACGTGCATAAATTCCATTTGCGCGTAATCGTGAGCCATGCAGCAAGCCATACACATGTGGTGGCAGCCGTCAGCTGTTTTGTGGCAGACAATGGCTTTGGCTTGCTGTGTGTGTTGAGGGGAAGTGAGTGACCCGATATGCTTCAAAGGCGGGTCGATTGCGCTCATTTTCTCGTTATTCGTAAAAACGGTATCCACTTTGGCGTCTACGCCGTACCTAGCTACTGCCTGGACCGTCGGTAGCGATGAAAGGTGCGGAGAAAGTTCCTCATTTTGAGTTGAGCAGACTTAATGAGCCCGGCAAGAGGGCCGCCAAAACCAAAACACAACCATTTCTTTATTTTTAATAGTTGATCGGTGAATCGGGGTGTTCGTTATGTGGCAACATGTGGTTGTCGCTGCGGATGTCTTGGTGAATCGTTAACTACAAATCTTGGCGGTGGATCACTCGGTTCGTGGATCGATGAAGAACGCAGCCAGCTGCGATAAATAGTGCGAATTGCAGACACATTGAGCACTAAAATTTCGAACGCACATTGCGCCATCGGGTTCATTCCCGCTGGCACGTCTGGCTGAGGGTCGAAATGTTCGACACTATCCGCACAATACTTCCTGTGAGCAGGGAAGTGTGTGGTGCATTCGGCAAGCAGTGATTCTTGAGCATTGCTCTCGAAAGCGCTCCTTGCTTAGACGGAACGGTAAATAATTGCTAAATTTTACTCGGTAAGGTGCAATTATAGCCGCGAATATGTTGCTTCTCGATGCGGCTTCCAGTATTTGTTGATTGTCGATAGCGACTGGAAGTATGGCATCGGATGCCTGATAGAGTGATATGTCTTGGTTGGTAATGTGTTTTTGACCTCAKCTCAGTCGTGATTACCCGCTGAATTTAAGCATATAATTAAKCGGAGGAAAAW

>ant-Nlar-31.612.1_NC5

TCTCCCAACGTGCATACCATCCATTTGCATGTTGTTGTGAGCCGCATAGAAACTCATACACGCGTGGTGGCAGCCGTCTGCTGTGCTTTATCGTGCAGACAATGGCTTATGAGTGGCTGTGTGATTGTTGAACAACGGTGACCAATTTGGCGTCTACGCCGTATCTAGCTTCCGCCTGGACCGTCGGTAGCGATGAAAGATGCGGAGGAAGTTCCTCTGTTTTGGTTTCAACGCTAACGCAGAGTTGAGCAGACTTAATGAGCCACGCTTGGTGGCCGCCAAAACCCAAAACACAACCAGTCTATTTTAACGTTTGTTGATATGTTAATGTACAAATCTTGGCGGTG

>ant-Nlar-32.615.1_NC5

GTGCATAAATTCCATTTGCGCGTAATCGTGAGCCATGCAGCAAGCCATACACATGTGGTGGCAGCCGTCAGCTGTTTTGTGGCAGACAATGGCTTTGGCTTGCTGTGTGTGTTGAGGGGAAGTGAGTGACCCGATATGCTTCAAAGGCGGGTCGATTGCGCTCATTTTCTCGTTATTCGTAAAAACGGTATCCACTTTGGCGTCTACGCCGTACCTAGCTACTGCCTGGACCGTCGGTAGCGATGAAAGGTGCGGAGAAAGTTCCTCATTTTGAGTTGAGCAGACTTAATGAGCCCGGCAAGAGGGCCGCCAAAACCAAAACACAACCATTTCTTTATTTTTAATAGTTGATCGGTGAATCGGGGTGTTCGTTATGTGGCAACATGTGGTTGTCGCTGCGSATGTCTTGGKGAATCGNTAACTACAAATCTTGGSGGTGGATCACTCGGTTCGTGSATCGATGAAGAACGCAGCCAGCTGCGATAAATAGTGCGAATTGCAGACACATTGAGCACTAAAATTTCGAACGCACATTGCGCCATCGKKTTCATTCCCGCTGSCACGTCTGGSTGAGKSRCGAAATGTGCGACACTATCCGCAYAATACTTCCTGTGAGCA

>ant-Nlar-33.616.1_NC5

NNTCTCCCAACGTGCATACCATCCATTTGCATGTTGTTGTGAGCCGCATAGAAACTCATACACGCGTGGTGGCAGCCGTCTGCTGTGCTTTATCGTGCAGACAATGGCTTATGAGTGGCTGTGTGATTGTTGAACAACGGTGACCAATTTGGCGTCTACGCCGTATCTAGCTTCCGCCTGGACCGTCGGTAGCGATGAAAGATGCGGAGGAAGTTCCTCTGTTTTGGTTTCAACGCTAACGCAGAGTTGAGCAGACTTAATGAGCCACGCTTGGTGGCCGCCAAAACCCAAAACACAACCAGTCTATTTTAACGTTTGTTGATATGTTAATGTACAAATCTTGGCGGTGGATCACTCGGTTCGTGGATCGATGAAGAACGCAGCCAGCTGCGATAAATAGTGCGAATTGCAGACACATTGAGCACTAAGAATTCGAACGCACATTGCGCTATCGGGTTCATTCCCGATGGCACGTCTGGCTGAGGGTCGAATTGTAGTAAACTGTCTTCAAGTACTTTTTATGGTCGTGAAGTATTCGGCAAGCAGTTGTCGGATAGTTGTTTTTGTTGATCGTCCGTTCGTTCGGTCGGTCCAGAGCAATATTCTGAGGCTCCTTGCTTAGTTGCGTTTTGGTAGACGTTAACACCCGAACAATATGTGGTGKTGATATTGKTGWTGC

>ant-Nlar-34.620.1_NC5

NTCTCCCAACGTGCATACCATCCATTTGCATGTTGTTGTGAGCCGCATAGAAACTCATACACGCGTGGTGGCAGCCGTCTGCTGTGCTTTATCGTGCAGACAATGGCTTATGAGTGGCTGTGTGATTGTTGAACAACGGTGACCAATTTGGCGTCTACGCCGTATCTAGCTTCCGCCTGGACCGTCGGTAGCGATGAAAGATGCGGAGGAAGTTCCTCTGTTTTGGTTTCAACGCTAACGCAGAGTTGAGCAGACTTAATGAGCCACGCTTGGTGGCCGCCAAAACCCAAAACACAACCAGTCTATTTTAACGTTTGTTGATATGTTAATGTACAAATCTTGGCGGTGGATCACTCGGTTCGTGGATCGATGAAGAACGCAGCCAGCTGCGATAAATAGTGCGAATTGCAGACACATTGAGCACTAAGAATTCGAACGCACATTGCGCTATCGGGTTCATTCCCGATGGCACGTCTGGCTGAGGGTCGAATTGTAGTAAACTGTCTTCAAGTACTTTTTATGGTCGTGAAGTATTCGGCAAGCAGTTGTCGGATAGTTGTTTTTGTTGATCGTCCGTTCGTTCGGTCGGTCCAGAGCAATATTCTGAGGCTCCTTGCTTAGTTGCGTTTTGGTAGACGTTAACACCCGAACAATATGYGGWGKNGATATTGKTGATGC

>ant-Nlar-35.623.1_NC5

ATAAATTCCNTTTGCGCGTAATCGTGAGCCATGCAGCAAGCCATACACATGTGGTGGCAGCCGTCAGCTGTTTTGTGGCAGACAATGGCTTTGGCTTGCTGTGTGTGTTGAGGGGAAGTGAGTGACCCGATATGCTTCAAAGGCGGGTCGATTGCGCTCATTTTCTCGTTATTCGTAAAAACGGTATCCACTTTGGCGTCTACGCCGTACCTAGCTACTGCCTGGACCGTCGGTAGCGATGAAAGGTGCGGAGAAAGTTCCTCATTTTGAGTTGAGCAGACTTAATGAGCCCGGCAAGAGGGCCGCCAAAACCAAAACACAACCATTTCTTTATTTTTAATAGTTGATCGGTGAATCGGGGTGTTCGTTATGTGGCAACATGTGGTTGTCGCTGCGGATGTCTTGGTGAATCGTTAACTACAAATCTTGGCGGTGGATCACTCGGTTCGTGGATCGATGAAGAACGCAGCCAGCTGCGATAAATAGTGCGAATTGCAGACACATTGAGCACTAAAATTTCGAACGCACATTGCGCCATCGGGTTCATTCCCGCTGGCACGTCTGGCTGAGGGTCGAAATGTTCGACACTATCCGCACAATACTTCCTGTGAGCAGGGAAGTGTGTGGTGCATTCGGCAAGCAGTGATTCTTGAGCATTGCTCTCGAAAGCGCTCCTTGCTTAGACGGAACGGTAAATAATTGCTAAATTTTACTCGGTAAGGTGCAATTATAGCCGCGAATATGTTGCTTCTCGATGCGGCTTCCAGTATTTGTTGATTGTCGATAGCGACTGGAAGTATGGCATCGGATGCCTGATAGAGTGATATGTCTTGKTTGTTATGNGTTTTTGACCTCAGCTCAGTCGTGATTACCCGCTGAATTTAAKCATATAAT

>ant-Nlar-37.629.1_NC5

NTCTCCCAACGTGCATACCATCCATTTGCATGTTGTTGTGAGCCGCATAGAAACTCATACACGCGTGGTGGCAGCCGTCTGCTGTGCTTTATCGTGCAGACAATGGCTTATGAGTGGCTGTGTGATTGTTGAACAACGGTGACCAATTTGGCGTCTACGCCGTATCTAGCTTCCGCCTGGACCGTCGGTAGCGATGAAAGATGCGGAGGAAGTTCCTCTGTTTTGGTTTCAACGCTAACGCAGAGTTGAGCAGACTTAATGAGCCACGCTTGGTGGCCGCCAAAACCCAAAACACAACCAGTCTATTTTAACGTTTGTTGATATGTTAATGTACAAATCTTGGCGGTGGATCACTCGGTTCGTGGATCGATGAAGAACGCAGCCAGCTGCGATAAATAGTGCGAATTGCAGACACATTGAGCACTAAGAATTCGAACGCACATTGCGCTATCGGGTTCATTCCCGATGGCACGTCTGGCTGAGGGTCGAATTGTAGTAAACTGTCTTCAAGTACTTTTTATGGTCGTGAAGTATTCGGCAAGCAGTTGTCGGATAGTTGGTTTTTGTTGATCGTCCGTTCGTTCGGTCGGTCCAGAGCAATATTCTGAGGCTCCTTGCTTAGTTGCGTTTTGGTAGACGTTAACACCCGAACATATGTGGTGGTGAWAWTTGGTKGANNGNCAANAATCATGCCGCTTCAATGGGGCAGCAACCAGCATACGCTAATGACAGTTGGTTGATTGAAGCCAGGNCAACGGAGTGATGTGTGGCGATCATTTAACGTTTGTATTTGACCTCAGCTCAGTCGTGATTACCCGCTGAATTTAAGCATATAATTATCCGAGA

>ant-Nlar-38.633.1_NC5

TCTCCCAACGTGCATACCATCCATTTGCATGTTGTTGTGAGCCGCATAGAAACTCATACACGCGTGGTGGCAGCCGTCTGCTGTGCTTTATCGTGCAGACAATGGCTTATGAGTGGCTGTGTGATTGTTGAACAACGGTGACCAATTTGGCGTCTACGCCGTATCTAGCTTCCGCCTGGACCGTCGGTAGCGATGAAAGATGCGGAGGAAGTTCCTCTGTTTTGGTTTCAACGCTAACGCAGAGTTGAGCAGACTTAATGAGCCACGCTTGGTGGCCGCCAAAACCCAAAACACAACCAGTCTATTTTAACGTTTGTTGATATGTTAATGTACAAATCTTGGCGGTGGATCACTCGGTTCGTGGATCGATGAAGAACGCAGCCAGCTGCGATAAATAGTGCGAATTGCAGACACATTGAGCACTAAGAATTCGAACGCACATTGCGCTATCGGGTTCATTCCCGATGGCACGTCTGGCTGAGGGTCGAATTGTAGTAAACTGTCTTCAAGTACTTTTTATGGTCGTGAAGTATTCGGCAAGCAGTTGTCGGATAGTTGTTTTTGTTGATCGTCCGTTCGTTCGGTCGGTCCAGAGCAATATTCTGAGGCTCCTTGCTTAGTTGCGTTTTGGTAGACGTTAACACCCGAACAATATGTGGTGGTGATATTGGTGATGNCGAGAATCATGCCGCTTCAATGGGGCAGCAACCAGCATACGCTAATGACAGWTGGTTGATTGAAGCCAGGNCAACGGAGTGATGTGTGGCGATCATTRARCGTTTGKATTTGACCTCAGSTCAGCCGWGATTACCCGTTGAATTTMAGCATATAATKATACG

>ant-Nlar-38.635.1_NC5

GTGCATAAATTCCATTTGCGCGTAATCGTGAGCCATGCAGCAAGCCATACACATGTGGTGGCAGCCGTCAGCTGTTTCGTGGCAGACAATGGCTTTGGCTTGCTGTGTGTGTTGAGGGGAAGTGAGTGACCCGATATGCTTCAAAGGCGGGTCGATTGCGCTCATTTTCTCGTTATTCGTAAAAACGGTATCCACTTTGGCGTCTACGCCGTACCTAGCTACTGCCTGGACCGTCGGTAGCGATGAAAGGTGCGGAGAAAGTTCCTCATTTTGAGTTGAGCAGACTTAATGAGCCCGGCAAGAGGGCCGCCAAAACCAAAACACAACCATTTCTTTATTTTTAATAGTTGATCGGTGAATCGGKGTGTTCGTTATGTGGCAACATGTGGTTGTCGCTGCGGATGTCTTGSTGAATCGTTAACTACAAATCTTGGCGRTGGATCACTCGGTTCGTGGATCGATGAAGAACGCAGCCAGCTGCGATAAATAGTGCGAATTGCAGACACATTGAGCACTAAAATTTCGAACGCACATTGCGCCATCGGSTTCATTCCCGCTGGCACGTCTGGCTGAGGGTCGAAATGTTCGACACTATCCGCACAATACTTCCTGTGAGCAGGGAAGTGTGTGGTGCATTCGGCAAGCAGTGATTCTTGAGCATTGCTCTCGAAAGCGCTCCTTGCTTAGACGGAACGGTAAATAATTGCTAAATTTTACTCGGTAAGGTGCAATTATAGCCGCGAATATGTTGCTTCTCGATGCGGCTTCCAGTATTTGTTGATTGTCGATAGCGACTGGAAGTATGGCATCGGATGCCTGATAGAGTGATATGTCTTGGTTGTTATGNGNTTTTGN

>ant-Nlar-38.637.1_NC5

TACGTGCATAAATTCCATTTGTGCGTAATCGTGAGCCATGCATCAAGCCATACACATGTGGTGGCAGCCGTCCGCTGTTTTGTGGCAGACAATGGCTTTGGCTTGATGTGTGATGAGGGGAAN

>ant-Nlar-4.534.1_NC5

CGAGCGAATCCAAAACGAAAAAGTCTCCCAACGTGCATACCATCCATTTGCATGTTGTTGTGAGCCGCATAGAAACTCATACACGCGTGGTGGCAGCCGTCTGCTGTGCTTTATCGTGCAGACAATGGCTTATGAGTGGCTGTGTGATTGTTGAACAACGGTGACCAATTTGGCGTCTACGCCGTATCTAGCTTCCGCCTGGACCGTCGGTAGCGATGAAAGATGCGGAGGAAGTTCCTCTGTTTTGGTTTCAACGCTAACGCAGAGTTGAGCAGACTTAATGAGCCACGCTTGGTGGCCGCCAAAACCCAAAACACAACCAGTCTATTTTAACGTTTGTTGATATGTTAATGTACAAATCTTGGCGGTGGATCACTCGGTTCGTGGATCGATGAAGAACGCAGCCAGCTGCGATAAATAGTGCGAATTGCAGACACATTGAGCACTAAGAATTCGAACGCACATTGCGCTATCGGGTTCATTCCCGATGGCACGTCTGGCTGAGGGTCGAATTGTAGTAAACTGTCTTCAAGTACTTTTTATGGTCGTGAAGTATTCGGCAAGCAGTTGTCGGATAGTTGTTTTTGTTGATCGTCCGTTCGTTCGGTCGGTCCAGAGCAATATTCTGAGGCTCCTTGCTTAGTTGCGTTTTGGTAGACGTTAACACCCGAACAATATGTGGTGGTGATATTGGTGATGGCGAGAATCATGCCGCTTCAATGGGGCAGCAACCAGCATACGCTAATGACAGTTGGTTGATTGAAGCCAGGGCAACGGAGTGATGTGTGGCGATCATTTAACGTTTGTATTTGACCTCAGCTCAGTCGTGATTACCCGCTGAATTTAAGCATATAATTAAGCGAGAAANNNAAA

>ant-Nlar-4.535.1_NC5

GAATCCAAAACGAAAAAGTCTCCCAACGTGCATACCATCCATTTGCATGTTGTTGTGAGCCGCATAGAAACTCATACACGCGTGGTGGCAGCCGTCTGCTGTGCTTTATCGTGCAGACAATGGCTTATGAGTGGCTGTGTGATTGTTGAACAACGGTGACCAATTTGGCGTCTACGCCGTATCTAGCTTCCGCCTGGACCGTCGGTAGCGATGAAAGATGCGGAGGAAGTTCCTCTGTTTTGGTTTCAACGCTAACGCAGAGTTGAGCAGACTTAATGAGCCACGCTTGGTGGCCGCCAAAACCCAAAACACAACCAGTCTATTTTAACGTTTGTTGATATGTTAATGTACAAATCTTGGCGGTGGATCACTCGGTTCGTGGATCGATGAAGAACGCAGCCAGCTGCGATAAATAGTGCGAATTGCAGACACATTGAGCACTAAGAATTCGAACGCACATTGCGCTATCGGGTTCATTCCCGATGGCACGTCTGGCTGAGGGTCGAATTGTAGTAAACTGTCTTCAAGTACTTTTTATGGTCGTGAAGTATTCGGCAAGCAGTTGTCGGATAGTTGTTTTTGTTGATCGTCCGTTCGTTCGGTCGGTCCAGAGCAATATTCTGAGGCTCCTTGCTTAGTTGCGTTTTGGTAGACGTTAACACCCGAACAATATGTGGTGGTGATATTTGGGTGATGGCNGAGAATCATGCCGCTTCAATGGGGCAGCAACCAGCATACGCTAATGACAGTTGGTTGATTGAAGCCAGGGCAACGGAGTGATGTGTGGCGATCATTTAACGTTTGTATTTGACCTCAGCTCAGTCGTGATTACCCGCTGAATTTAAGCATATAATTAAGCGGAGAAAANANA

>ant-Nlar-40.643.1_NC5

NTCTCCCAACGTGCATACCATCCATTTGCATGTTGTTGTGAGCCGCATAGAAACTCATACACGCGTGGTGGCAGCCGTCTGCTGTGCTTTATCGTGCAGACAATGGCTTATGAGTGGCTGTGTGATTGTTGAACAACGGTGACCAATTTGGCGTCTACGCCGTATCTAGCTTCCGCCTGGACCGTCGGTAGCGATGAAAGATGCGGAGGAAGTTCCTCTGTTTTGGTTTCAACGCTAACGCAGAGTTGAGCAGACTTAATGAGCCACGCTTGGTGGCCGCCAAAACCCAAAACACAACCAGTCTATTTTAACGTTTGTTGATATGTTAATGTACAAATCTTGGCGGTGGATCACTCGGTTCGTGGATCGATGAAGAACGCAGCCAGCTGCGATAAATAGTGCGAATTGCAGACACATTGAGCACTAAGAATTCGAACGCACATTGCGCTATCGGGTTCATTCCCGATGGCACGTCTGGCTGAGGGTCGAATTGTAGTAAACTGTCTTCAAGTACTTTTTATGGTCGTGAAGTATTCGGCAAGCAGTTGTCGGATAGTTGTTTTTGTTGATCGTCCGTTCGTTCGGTCGGTCCAGAGCAATATTCTGAGGCTCCTTGCTTAGTTGCGTTTTGGTAGACGTTAACACCCGAACAATATGTGGTGGTGATATTGKTGATGNCGAGAMTCATGCCGCTTCAAYGSNGNTKGYNCGASCATACGCTAATGACAGATGGYTGMYTGCAGGCRGGY

>ant-Nlar-5.536.1_NC5

NTCTCCCAACGTGCATACCATCCATTTGCATGTTGTTGTGAGCCGCATAGAAACTCATACACGCGTGGTGGCAGCCGTCTGCTGTGCTTTATCGTGCAGACAATGGCTTATGAGTGGCTGTGTGATTGTTGAACAACGGTGACCAATTTGGCGTCTACGCCGTATCTAGCTTCCGCCTGGACCGTCGGTAGCGATGAAAGATGCGGAGGAAGTTCCTCTGTTTTGGTTTCAACGCTAACGCAGAGTTGAGCAGACTTAATGAGCCACGCTTGGTGGCCGCCAAAACCCAAAACACAACCAGTCTATTTTAACGTTTGTTGATATGTTAATGTACAAATCTTGGCGGTGGATCACTCGGTTCGTGGATCGATGAAGAACGCAGCCAGCTGCGATAAATAGTGCGAATTGCAGACACATTGAGCACTAAGAATTCGAACGCACATTGCGCTATCGGGTTCATTCCCGATGGCACGTCTGGCTGAGGGTCGAATTGTAGTAAACTGTCTTCAAGTACTTTTTATGGTCGTGAAGTATTCGGCAAGCAGTTGTCGGATAGTTGGTTTTTGTTGATCGTCCGTTCGTTCGGTCGGTCCAGAGCAATATTCTGAGGCTCCTTGCTTAGTTGCGTTTTGGTAGACGTTAACACCCGAACAATATGTGGTGGTGATATTTGNTGATGNCNANAATCATGCCGCTTCAATGGGGCAGCAACCAGCATACGCTAATGACAGWTGGYTGATTGAAGCCAGGRCAACGGAGTGATGTGTGRCGATCATTTAACGTWTGTATTTGACCTCAGCTCAGTCGTGATTACCCGCTGAATTTAAGCATATAATTATKCGGAGGA

>ant-Nlar-5.537.1_NC5

GTGCATAAATTCCATTTGCGCGTAATCGTGAGCCATGCAGCAGGCCATACACATGTGGTGGCAGCCGTCAGCTGTTTTGTGGCAGACAATGGCTTTGGCTTGCTGTGTGTGTTGAGGGGAAGTGAGTGACCCGATATGCTTCAAAGGCGGGTCGAATGCGCTCATTTTCTCGTTATTCGTAAAAACGGTATCCACTTTGGCGTCTACGCCTCACCTAGCTACTGCCTGGACCGTCGGTAGCTATGAAAGGTGCGGAGAAAGTTCCTCATTTTGAGTTGAGCAGACTTAATGAGCCCGGCAAGAGGGCCGCCAAAACCAAAACACAACCATTTCTTTATTTTTAATAGTTGATCGGTGAATCGGGGTGTTCGTTATGTGGCAACATGTGGTTGTCGCTGCGGATGTCTCGGTGAATCGTTAACTACAAATCTTGGCGGTGGATCACTCGGTTCGTGGATCGATGAAGAACGCAGCCAGCTGCGATAAATAGTGCGAATTGCAGACACATTGAGCACTAAAATTTCGAACGCACATTGCGCCATCGGGTTCATTCCCGCTGGCACGTCTGGCTGAGGGTCGAAATGTTCGACACTATCCGCACAGGGTTTCCTACGATTAGGGAACTGTGTGTGGTGCATTCGGCAAGCAGTGATTCTTGAGCGTTGCTCTCGAAAGCGCTCCTTGCTTAGACGGAACGGTAAATAATTGCTAAATTTTACTCGGTAAGGTGCAATTATGGWCGCGAATATGTTGCTTCTCGATGCGGCTTCCAGTATTTGTTGATTGTCGATAGTGACTGGAAGTATGNCATCGYATGCCTGATAGA

>ant-Nlar-8.544.1_NC5

AACGTGCATACCATCCATTTGCATGTTGTTGTGAGCCGCATAGAAACTCATACACGCGTGGTGGCAGCCGTCTGCTGTGCTTTATCGTGCAGACAATGGCTTATGAGTGGCTGTGTGATTGTTGAACAACGGTGACCAATTTGGCGTCTACGCCGTATCTAGCTTCCGCCTGGACCGTCGGTAGCGATGAAAGATGCGGAGGAAGTTCCTCTGTTTTGGTTTCAACGCTAACGCAGAGTTGAGCAGACTTAATGAGCCNCGCTTGGNGGCCGCCAAAACCCAAAACACAACCAGTCTATTTTAACGTTTGTTGATATGTTA

>ant-Nnud-1.373.1_NC5

CCCARCGTGCATACCATCCATTTGCATGTTGTTGTGAGCCGCATAGAAACTCATACACGCGTGGTGGCAGCCGTCTGCTGTGCTTTATCGTGCAGACAATGGCTTATGAGTGGCTGTGTGATTGTTGAACAACGGTGACCAATTTGGCGTCTACGCCGTATCTAGCTTCCGCCTGGACCGTCGGTAGCGATGAAAGATGCGGAGGAAGTTCCTCTGTTTTGGTTTCAACGCTAACGCAGAGTTGAGCAGACTTAATGAGCCACGCTTGGTGGCCGCCAAAACCCAAAACACAACCAGTCTATTTTAACGTTTGTTGATATGTTAATGTACAAATCTTGGCGGTGGATCACTCGGTTCGTGGATCGATGAAGAACGCAGCCAGCTGCGATAAATAGTGCGAATTGCAGACACATTGAGCACTAAGAATTCGAACGCACATTGCGCTATCGGGTTCATTCCCGATGGCACGTCTGGCTGAGGGTCGAATTGTAGTAAACTGTCTTCAAGTACTTTTTATGGTCGTGAAGTATTCGGCAAGCAGTTGTCGGATAGTTGTTTTTGTTGATCGTCCGTTCGTTCGGTCGGTCCAGAGCAATATTCTGAGGCTCCTTGCTTAGTTGCGTTTTGGTAGACGTTAACACCCGAACAATATGTGGTGGTGATATTTGGTGATGGCGAGAATCATGCCGCTTCAATGGGGCAGCAACCAGCATACGCTAATGACAGTTGGTTGATTGAAGCCAGGGCAACGGAGTGATGTGTGGCGATCATTTAACGTTTGTATTTGACCTCAGCTCAGTCGTGATTACCCGCTGAATTTAAGCATATAATTAAGCGGAGAAAANNN

>ant-Nnud-1.375.1_NC5

GCATAAATTCCATTTGCGCGTAATCGTGAGCCATGCAGCAAGCCATACACATGTGGTGGCAGCCGTCAGCTGTTTTGTGGCAGACAATGGCTTTGGCTTGCTGTGTGTGTTGAGGGGAAGTGAGTGACCCGATATGCTTCAAAGGCGGGTCGATTGCGCTCATTTTCTCGTTATTCGTAAAAACGGTATCCACTTTGGCGTCTACGCCGTACCTAGCTACTGCCTGGACCGTCGGTAGCGATGAAAGGTGCGGAGAAAGTTCCTCATTTTGAGTTGAGCAGACTTAATGAGCCCGGCAAGAGGGCCGCCAAAACCAAAACACAACCATTTCTTTATTTTTAATAGTTGATCGGTGAATCGGGGTGTTCGTTATGTGACAACATGTGGTTGTCGCTGCGGATGTCTTGGTGAATCGTTAACTACAAATCTTGGCGGTGGATCACTCGGTTCGTGGATCGATGAAGAACGCAGCCAGCTGCGATAAATAGTGCGAATTGCAGACACATTGAGCACTAAAATTTCGAACGCACATTGCGCCATCGGGTTCATTCCCGCTGGCACGTCTGGCTGAGGGTCGAAATGTTCGACACTATCCGCACAATACTTCCTGTGAGCAGGGAAGTGTGTGGTGCATTCGGCAAGCAGTGATTCTTGAGCATTGCTCTCGAAAGCGCTCCTTGCTTAGACGGAACGGTAAATAATTGCTAAATTTTACTCGGTAAGGTGCAATTATAGCCGCGAATATGTTGCTTCTCGATGCGGCTTCCAGTATTTGTTGATTGTCGATAGCGACTGGAAGTATGGCATCGGATGCCTGATAGAGTGATATGTCTTGGTTGGTTATGTGTTTTTGACCTCAGCTCAGNCGTGATTACCCGCTGAATTTAAGCATATAATTAAGCGGAGG

>ant-Nnud-11.413.1_93

TGTCTGCCTTATCAACTTTCGATGGTAGTTTATGTGCCTACCATGGTTGTAACGGGTAACGGAGAATAARGGTTCGACTCCGGARAGGGAGCCTGAGAAACGGCTACCACATCCAAGGAAGGCRGCAGGCGCGCAAATTACCCACTCTCGKCATGAGGAGGTAGTGACGAAAAATAACGAGACCGTTCTCTTCGAGGCCGGTTATCGGAATGGGTACAATTTAAACCCGTTAACGAGGATCTATGAGAGGGCAAGTCTGGTGCCAGCAGCCGCGGTAATTCCAGCTCTCAAAGTGTATATCGGCATTGCTGCGGGTAARAAGCTCGTAGTTGGATCTGCGCCTCAGGACCTGGTCCGTCTTTTGSACGAGAACTGGGATCCTAGGCKAGTACTGTCGGTTTTCCCTGCGTTGCCTTCATCGGTCGCGCAGGGTGGCCGGYGAGTTTACCTTGARAAAATTAGAGTGCTCAACGCGGKCTGACGCCTGAATACTCGTGCATGGAATAATGARATARGATCTCGGTTCTATTTTGTTGGTTTTCTGATCTGAGATCGGTCGCGCAGGGTGGCCGGYGAGTTTACCTTGAAAAAATTAGAGTGCTCAACGCGGRCTGACGCCTGAATACTCGTGCATGGAATAATGAGATAGGATCTCGGTTCTATTTTGTTGYTTTTCTGATCTGAGATAATGGTTA

>ant-Nnud-13.419.1_NC5

NGTCTCCCAACGTGCATACCATCCATTTGCATGTTGTTGTGAGCCGCATAGAAACTCATACACGCGTGGTGGCAGCCGTCTGCTGTGCTTTATCGTGCAGACAATGGCTTATGAGTGGCTGTGTGATTGTTGAACAACGGTGACCAATTTGGCGTCTACGCCGTATCTAGCTTCCGCCTGGACCGTCGGTAGCGATGAAAGATGCGGAGGAAGTTCCTCTGTTTTGGTTTCAACGCTAACGCAGAGTTGAGCAGACTTAATGAGCCACGCTTGGTGGCCGCCAAAACCCAAAACACAACCAGTCTATTTTAACGTTTGTTGATATGTTAATGTACAAATCTTGGCGGTGGATCACTCGGTTCGTGGATCGATGAAGAACGCAGCCAGCTGCGATAAATAGTGCGAATTGCAGACACATTGAGCACTAAGAATTCGAACGCACATTGCGCTATCGGGTTCATTCCCGATGGCACGTCTGGCTGAGGGWCGAATTGTAGTAAACTGTCTTCTGTACTTTTTATGK

>ant-Nnud-14.429.1_93

TTGGTGACTCTGAATAGCTATGGCTGATCGCATGGTCTTGTACCGGCGACATGTCTATCAMGTGTCTGCCTTATCAACTTTCGATGGTAGTTTATGTGCCTACCATGGTTGTAACGGGTAACGGASAATAARGGTTCGACTCCGGARAGGGAGCCTGAGAAACGGCTACCACATCCMAGGAAGGCRGCARGCGCGCAAATTACCCACTCTCGKCATGAGGAGGTAGTGACGAAAAATAACGAGACCGTTCTCTTCGAGGCCGGTTATCGGAATGGGTACAATTTAAACCCGTTAACGAGGATCTATGAGAGGGCAAGTCTGGTGCCAGCAGCCGCGGTAATTCCAGCTCTCAAAGTGTATATCGKCATTGCTGCGKKTAARAAGCTCGTAGTTGGATCTGCGCCTCAGGACCTGGTCCGTCTTTTGSACGAGAACTGGGATCCTAGCTGCGGGTAARAAGCTCGTAGTTGGATCTGCGCCTCAGGACCTGGTCCGTCTTTTGGACGAGAACTGGGATCCTAGGCTAGTACTGTCGTTTTTCCCTGCGTTGCCTTCATCGGTCG

>ant-Nnud-15.435.1_93

TTTGCTCGTCAATTGGTGACTCTGAATAGCTATGGCTGATCGCATGGTCTTGTACCGGCGACATGTCTATCAAGTGTCTGCCTTATCAACTTTCGATGGTAGTTTATGTGCCTACCATGGTTGTAACGGGTAACGGAGAATAARGGTTCGACTCCGGARAGGGAGCCTGAGAAACGGCTACCACATCCMAGGAAGGCRGCARGCGCGCAAATTACCCACTCTCGKCATGAGGAGGTAGTGACGAAAAATAACGAGACCGTTCTCTTCGAGGCCGGTTATCGGAATGGGTACAATTTAAACCCGTTAACGAGGATCTATGAGAGGGCAAGTCTGGTGCCAGCAGCCGCGGTAATTCCAGCTCTCAAAGTGTATATCGKCATTGCTGCGGKTAARAAGCTCGTAGTTGGATCTGCGCCTCAGGACCTGGTCCGTCTTTTGSACGAGAACTGGGATCCTAGGCKAGTACTGTCGGTTTTCCCTGCGTTGCCTTCATCGGTCGMGCAGGGTGGCCGGYGAGTTTACCTTGAAAAAATTAGAGTGCTCAACGCGGKCTGACGCCTGAATACTCGTGCATGGAATAATGARATAGGATCTCGGTTCTATTTTGTTGGTTTTCTGATCTGAACTCGTGCATGGAATAATGAAATAGGATCTCGGTTCTATTTTGTTGGTTTTCTGATCTGAGATAATGGTTAAGA

>ant-Nnud-18.451.1_93

TCGTCAATTGGTGACTCTGAATAGCTATGGCTGATCGCATGGTCTTGTACCGGCGACATGTCTATCAAGTGTCTGCCTTATCAACTTTCGATGGTAGTTTATGTGCCTACCATGGTTGTAACGGGTAACGGAGAATAARGGTTCGACTCCGGARAGGGAGCCTGAGAAACGGCTACCACATCCMAGGAAGGCRGCARGCGCGCAAATTACCCACTCTCGKCATGAGGAGGTAGTGACGAAAAATAACGAGACCGTTCTCTTCGAGGCCGGTTATCGGAATGGGTACAATTTAAACCCGTTAACGAGGATCTATGAGAGGGCAAGTCTGGTGCCAGCAGCCGCGGTAATTCCAGCTCTCAAAGTGTATATCGKCATTGCTGCGGKTAARAAGCTCGTAGTTGGATCTGCGCCTCAGGACCTGGTCCGTCTTTTGSACGAGAACTGGGATCCTAGGCKAGTACTGTCGGTTTTCCCTGCGTTGCCTTCATCGGTCGTCCGTCTTTTGGACGAGAACTGGGATCCTAGGCTAGTACTGTCGGTTTTCCCTGCGTTGCCTTCATCGGTCGCGCAGGGTGGCCGG

>ant-Nnud-19.455.1_93

TCGTCAATTGGTGACTCTGAATAGCTATGGCTGATCGCATGGTCTTGTACCGGCGACATGTCTATCAMGTGTCTGCCTTATCAACTTTCGATGGTAGTTTATGTGCCTACCATGGTTGTAACGGGTAACGGAGAATAARGGTTCGACTCCGGARAGGGAGCCTGAGAAACGGCTACCACATCCAAGGAAGGCRGCARGCGCGCAAATTACCCACTCTCGKCATGAGGAGGTAGTGACGAAAAATAACGAGACCGTTCTCTTCGAGGCCGGTTATCGGAATGGGTACAATTTAAACCCGTTAACGAGGATCTATGAGAGGGCAAGTCTGGTGCCAGCAGCCGCGGTAATTCCAGCTCTCAAAGTGTATATCGKCATTGCTGCGGKTAARAAGCTCGTAGTTGGATCTGCGCCTCAGGACCTGGTCCGTCTTTTGGACGAGAACTGGGATCCTAGGCKAGTACTGTCGGTTTTCCCTGCGTTGCCTTCATCGGTCGCGCAGGGTGGCCGGYGAGTTTACCTTGAAAAAATTAGAGTGCTCAACGCGGKCTGACGCCTGAATACTCGTGCATGGAATAATGARATAGGATCTCGGTTCTATTTTGTTGGTTTTCTGATCTGAGATA

>ant-Nnud-2.379.1_NC5

TCTCCCAACGTGCATACCATCCATTTGCATGTTGTTGTGAGCCGCATAGAAACTCATACACGCGTGGTGGCAGCCGTCTGCTGTGCTTTATCGTGCAGACAATGGCTTATGAGTGGCTGTGTGATTGTTGAACAACGGTGACCAATTTGGCGTCTACGCCGTATCTAGCTTCCGCCTGGACCGTCGGTAGCGATGAAAGATGCGGAGGAAGTTCCTCTGTTTTGGTTTCAACGCTAACGCAGAGTTGAGCAGACTTAATGAGCCACGCTTGGTGGCCGCCAAAACCCAAAACACAACCAGTCTATTTTAACGTTTGTTGATATGTTAATGTACAAATCTTGGCGGTGGATCACTCGGTTCGTGGATCGATGAAGAACGCAGCCAGCTGCGATAAATAGTGCGAATTGCAGACACATTGAGCACTAAGAATTCGAACGCACATTGCGCTATCGGGTTCATTCCCGATGGCACGTCTGGCTGAGGGTCGAATTGTAGTAAACTGTCTTCAAGTACTTTTTATGGTCGTGAAGTATTCGGCAAGCAGTTGTCGGATAGTTGTTTTTGTTGATCGTCCGTTCGTTCGGTCGGTCCAGAGCAATATTCTGAGGCTCCTTGCTTAGTTGCGTTTTGGTAGACGTTAACACCCGAACAATATGTGGTGGTGATATTTGGTGATGGCGAGAATCATGCCGCTTCAATGGGGCAGCAACCAGCATACGCTAATGACAGTTGGTTGATTGAAGCCAGGGCAACGGAGTGATGTGTGGCGATCATTTAACGTTTGTATTTGACCTCAGCTCAGTCGTGATTACCCGCTGAATTTAAGCATATAATTAAGCGGAGAAANNNAAA

>ant-Nnud-21.460.1_93

TTGGTGACTCTGAATAGCTATGGCTGATCGCATGGTCTTGTACCGGCAGCATGTCTATCAMGTGTCTGCCTTATCAACTTTCGATGGTAGTTTATGTGCCTACCATGGTTGTAACGGGTAACGGAGAATAARGGTTCGACTCCGGARAGGGAGCCTGAGAAACGGMTACCACATCCAAGGAAGGCRGCARGCGCGCAAATTACCCACTCTCGKCATGAGGAGGTAGTGACGAAAAATAACGAGACCGTTCTCTTCGAGGCCGGTTATCGGAATGGGTACAATTTAAACCCGTTAACGAGGATCTATGAGAGGGCAAGTCTGGTGCCAGCAGCCGCGGTAATTCCAGCTCTCAAAGTGTATATCGKCATTGCTGCATGAGAGGGCAAGTCTGGTGCCAGCAGCCGCGGTAATTCCAGCTCTCAAAGTGTATATCGGCATTGCTGCGGGTAAGAAGCTCGTAGTTGGATCTGCGCCTCAGGACCTGGTCCGTCTTTT

>ant-Nnud-27.479.1_93

CTTTCGATGGTAGTTTATGTGCCTACCATGGTTGTAACGGGTAACGGAGAATAARGGTTCGACTCCGGARAGGGAGCCTGAGAAACGGMTACCACATCCMAGGAAGGCRGCARGCGCGCAAATTACCCACTCTCGKCATGAGGAGGTAGTGACGAAAAATAACGAGACCGTTCTCTTCGAGGCCGGTTATCGGAATGGGTACAATTTAAACCCGTTAACGAGGATCTATGAGAGGGCAAGTCTGGTGCCAGCAGCCGCGGTAATTCCAGCTCTCAAAGTGTATATCGKCATTGCTGCGKKTAARAAGCTCGTAGTTGGATCTGCGCCTCAGGACCTGGTCCGTCTTTTGSACGAGAACTGGGATCCTAGGCKAGTACTGTCGGTTTTCCCTGCGTTGCCTTCATCGGTCGCGCAKGGTGGCCGGYGAGTTTACCTTGAAAAAATTAGAGTGCTCAACGCGGKCTGACGCCTGAATACTCGTGCATGGAATAATGARATAGGATCTCGGTTCTATTTTGTTGGTTTTCTGATCTGAGATCCTGCGTTGCCTTCATCGGTCGCGCAGGGTGGCCGGYGAGTTTACCTTGAAAAAATTAGAGTGCTCAACGCGGGCTGACGCCTGAATACTCGTGCATGGAATAATGARATAGGATCTCGGTTCTATTTTGTTGGTTTTCTGATCTGAGATAATGGTTAA

>ant-Nnud-28.486.1_93_edit

TTTACTGGACGAGCGCATCTATTAGGTTAAAACCAATCGGGCTTTGCTCGTCAATTGGTGACTCTGAATAGCTATGGCTGATCGCATGGTCTTGTACCGGCGACATGTCTATCAAGTGTCTGCCTTATCAACTTTCGATGGTAGTTTATGTGCCTACCATGGTTGTAACGGGTAACGGAGAATAARGGTTCGACTCCGGAGAGGGAGCCTGAGAAACGGCTACCACATCCAAGGAAGGCRGCARGCGCGCAAATTACCCACTCTCGKCATGAGGAGGTAGTGACGAAAAATAACGAGACCGTTCTCTTCGAGGCCGGTTATCGGAATGGGTACAATTTAAACCCGTTAACGAGGATCTATGAGAGGGCAAGTCTGGTGCCAGCAGCCGCGGTAATTCCAGCTCTCAAAGTGTATATCGKCATTGCTGCGGKTAARAAGCTCGTAGTTGGATCTGCGCCTCAGGACCTGGTCCGTCTTTTGGACGAGAACTGGGATCCTAGGCKAGTACTGTCGGTTTTCCCTGCGTTGCCTTCATCGGTCGCGCAGGGTGGCCGGYGAGTTTACCTTGAAAAAATTAGAGTGCTCAACGCGGKCTGACGCCTGAATACTCGTGCATGGAATAATGARATAGGATCTCGGTTCTATTTTGTTGGTTTTCTGATCTGAGATAATGGATCTGAGATAATGGG

>ant-Nnud-33.496.1_NC5

GTGCATAAATTCCATTTGCGCGTAATCGTGAGCCATGCAGCAAGCCATACACATGTGGTGGCAGCCGTCAGCTGTTTTGTGGCAGACAATGGCTTTGGCTTGCTGTGTGTGTTGAGGGGAAGTGAGTGACCCGATATGCTTCAAAGGCGGGTCGATTGCGCTCATTTTCTCGTTATTCGTAAAAACGGTATCCACTTTGGCGTCTACGCCGTACCTAGCTACTGCCTGGACCGTCGGTAGCGATGAAAGGTGCGGAGAAAGTTCCTCATTTTGAGTTGAGCAGACTTAATGAGCCCGGCAAGAGGGCCGCCAAAACCAAAACACAACCATTTCTTTATTTTTAATAGTTGATCGGTGAATCGGGGTGTTCGTTATGTGGCAACATGTGGTTGTCGCTGCGGATGTCTTGGTGAATCGTTAACTACAAATCTTGGCGGTGGATCACTCGGTTCGTGGATCGATGAAGAACGCAGCCAGCTGCGATAAATAGTGCGAATTGCAGACACATTGAGCACTAAAATTTCGAACGCACATTGCGCCATCGGGTTCATTCCCGCTGGCACGTCTGGCTGAGGGTCGAAATGTTCGACACTATCCGCACAATACTTCCTGTGAGCAGGGAAGTGTGTGGTGCATTCGGCAAGCAGTGATTCTTGAGCATTGCTCTCGAAAGCGCTCCTTGCTTAGACGGAACGGTAAATAATTGCTAAATTTTACTCGGTAAGGTGCAATTATAGCCGCGAATATGTTGCTTCTCGATGCGGCTTCCAGTATTTGTTGATTGTCGATAGCGACTGGAAGTATGGCATCGGATGCCTGATAGAGTGATATGTCTTGGTTGTTAAKGTGTTTTTGACCTCAGCTCAGTCGTGATTACCCGCTGAATTTAAGCATATAATTAAGCGAGAAA

>ant-Nnud-35.503.1_93

TCAATTGGTGACTCTGAATAGCTATGGCTGATCGCATGGTCTTGTACCGGCGACATGTCTATCAAGTGTCTGCCTTATCAACTTTCGATGGTAGTTTATGTGCCTACCATGGTTGTAACGGGTAACGGAGAATAARGGTTCGACTCCGGARAGGGAGCCTGAGAAACGGMTACCACATCCAAGGAAGGCRGCARGCGCGCAAATTACCCACTCTCGKCATGAGGAGGTAGTGACGAAAAATAACGAGACCGTTCTCTTCGAGGCCGGTTATCGGAATGGGTACAATTTAAACCCGTTAACGAGGATCTATGAGAGGGCAAGTCTGGTGCCAGCAGCCGCGGTAATTCCAGCTCTCAAAGTGTATATCGKCATTGCTGCGAGAGGGCAAGTCTGGTGCCAGCAGCCGCGGTAATTCCAGCTCTCAAAGTGTATATCGGCATTGCTGCGGGTAARAAGCTCGTAGTTGGATCTGCGCCTCAGGACCTGGTCCGTCTTTTGGACGAGAACTGGGATCCTAGGCTAGTACTGTCGGTTTTCCCTGCGTTGCCTTCATCGGTCGCGCAGGGTGGCCGGYGAGTTTACCTTGAAAAAATTAGAGTGCTCAACGCGGGCTGACGCCTGAATACTCGTGCATGGAATAATG

>ant-Nnud-36.504.1_93

ATTGGTGACTCTGAATAGCTATGGCTGATCGCATGGTCTTGTACCGGCGACATGTCTATCAAGTGTCTGCCTTATCAACTTTCGATGGTAGTTTATGTGCCTACCATGGTTGTAACGGGTAACGGAGAATAARGGTTCGACTCCGGAGAGGGAGCCTGAGAAACGGCTACCACATCCAAGGAAGGCRGCARGCGCGCAAATTACCCACTCTCGKCATGAGGAGGTAGTGACGAAAAATAACGAGACCGTTCTCTTCGAGGCCGGTTATCGGAATGGGTACAATTTAAACCCGTTAACGAGGATCTATGAGAGGGCAAGTCTGGTGCCAGCAGCCGCGGTAATTCCAGCTCTCAAAGTGTATATCGKCATTGCTGCGGKTAARAAGCTCGTAGTTGGATCTGCGCCTCAGGACCTGGTCCGTCTTTTGGACGAGAACTGGGATCCTAGGCTAGTACTGTCGGTTTTCCCTGCGTTGCCTTCATCGGTCGMGCA

>ant-Nnud-38.509.1_93

GCATCTATTAGGTTAAAACCWATCGGGCTTTGCTCGTCAATTGGTGACTCTGAATAGCTATGGCTGATCGCATGGTCTTGTACCGGCGACATGTCTATCAAGTGTCTGCCTTATCAACTTTCGATGGTAGTTTATGTGCCTACCATGGTTGTAACGGGTAACGGAGAATAARGGTTCGACTCCGGAGAGGGAGCCTGAGAAACGGCTACCACATCCAAGGAAGGCRGCARGCGCGCAAATTACCCACTCTCGKCATGAGGAGGTAGTGACGAAAAATAACGAGACCGTTCTCTTCGAGGCCGGTTATCGGAATGGGTACAATTTAAACCCGTTAACGAGGATCTATGAGAGGGCAAGTCTGGTGCCAGCAGCCGCGGTAATTCCAGCTCTCAAAGTGTATATCGKCATTGCTGCGGKTAARAAGCTCGTAGTTGGATCTGCGCCTCAGGACCTGGTCCGTCTTTTGGACGAGAACTGGGATCCTAGGCKAGTACTGTCGGTTTTCCCTGCGTTGCCTTCATCGGTCGCGCAGGGTGGCCGGYGAGTTTACCTTGAAAAAATTAGAGTGCTCAACGCGGGCTGACGCCTGAATACTCGTGCATGGAATAATGARATAGGATCTCGGTTCTATTTTGTTGGTTTTCTGATCTGAGATAATGGTTAAGAGGGACGKACGGRTTTTGTTGGTTTTCTGATCTGAGATAATGGTTAAGAGGGACGKACGGRGGYATTC

>ant-Nnud-39.512.1_93

TTGGTGACTCTGAATAGCTATGGCTGATCGCATGGTCTTGTACCGGCGACATGTCTATCAMGTGTCTGCCTTATCAACTTTCGATGGTAGTTTATGTGCCTACCATGGTTGTAACGGGTAACGGAGAATAARGGTTCGACTCCGGARAGGGAGCCTGAGAAACGGMTACCACATCCAAGGAAGGCRGCARGCGCGCAAATTACCCACTCTCGKCATGAGGAGGTAGTGACGAAAAATAACGAGACCGTTCTCTTCGAGGCCGGTTATCGGAATGGGTACAATTTAAACCCGTTAACGAGGATCTATGAGAGGGCAAGTCTGGTGCCAGCAGCCGCGGTAATTCCAGCTCTCAAAGTGTATATCGYCATTGCTGCGGKTAARAAGCTCGTAGTTGGATCTGCGCCTCAGGACCTGGTCCGTCTTTTGRACGAGAACTGGGATCCTAGGCTAGTACTGTCGGTTTTCCCTGCGTTGCCTTCATC

>ant-Nnud-40.515.1_NC5

TCTCCCAACGTGCATACCATCCATTTGCATGTTGTTGTGAGCCGCATAGAAACTCATACACGCGTGGTGGCAGCCGTCTGCTGTGCTTTATCGTGCAGACAATGGCTTATGAGTGGCTGTGTGATTGTTGAACAACGGTGACCAATTTGGCGTCTACGCCGTATCTAGCTTCCGCCTGGACCGTCGGTAGCGATGAAAGATGCGGAGGAAGTTCCTCTGTTTTGGTTTCAACGCTAACGCAGAGTTGAGCAGACTTAATGAGCCACGCTTGGTGGCCGCCAAAACCCAAAACACAACCAGTCTATTTTAACGTTTGTTGATATGTTAATGTACAAATCTTGGCGGTGGATCACTCGGTTCGTGGATCGATGAAGAACGCAGCCAGCTGCGATAAATAGTGCGAATTGCAGACACATTGAGCACTAAGAATTCGAACGCACATTGCGCTATCGGGTTCATTCCCGATGGCACGTCTGGCTGAGGGTCGAATTGTAGTAAACTGTCTTCAAGTACTTTTTATGGTCGTGAAGTATTCGGCAAGCAGTTGTCGGATAGNTTGTTTTTGTTGATCGTCCGTTCGTTCGGTCGGTCCAGAGCAATATTCTGAGGCTCCTTGCTTAGTTGCGTTTTGGTAGACGTTAACACCCGAACAATATGTGGTGGTGATAWTKGNTGATGNCNANAATCATGCCGCTTCAATGNNGCAGCAACCAGCATACGCTAATGACAGTTGGTTGATTGAAGCCAGGNCAACGGAGTGATGTGTGGCGATCATTTAACGTTTGTATTTGACCTCAGCTCAGTCGTGATTACCCGCTGAATTTAAGCATATAATTANCGGA

>ant-Nnud-6.392.1_NC5

TCTCCCANCGTGCATACCATCCATTTGCATGTTGTTGTGAGCCGCATAGAAACTCATACACGCGTGGTGGCAGCCGTCTGCTGTGCTTTATCGTGCAGACAATGGCTTATGAGTGGCTGTGTGATTGTTGAACAACGGTGACCAATTTGGCGTCTACGCCGTATCTAGCTTCCGCCTGGACCGTCGGTAGCGATGAAAGATGCGGAGGAAGTTCCTCTGTTTTGGTTTCAACGCTAACGCAGAGTTGAGCAGACTTAATGAGCCACGCTTGGTGGCCGCCAAAACCCAAAACACAACCAGTCTATTTTAACGTTTGTTGATATGTTAATGTACAAATCTTGGCGGTGGATCACTCGGTTCGTGGATCGATGAAGAACGCAGCCAGCTGCGATAAATAGTGCGAATTGCAGACACATTGAGCACTAAGAATTCGAACGCACATTGCGCTATCGGGTTCATTCCCGATGGCACGTCTGGCTGAGGGTCGAATTGTAGTAAACTGTCTTCAAGTACTTTTTATGGTCGTGAAGTATTCGGCAAGCAGTTGTCGGATAGTTGTTTTTGTTGATCGTCCGTTCGTTCGGTCGGTCCAGAGCAATATTCTGAGGCTCCTTGCTTAGTTGCGTTTTGGTAGACGTTAACACCCGAACAATATGTGGTGGTGATATTGNTGATGNCGAGAATCATGCCGCTTCAATGGGGCAGCAACCAGCATACGCTAATGACAGTTGGTTGATTGAAGCCAGGNCAACGGAGTGATGTGTGKCGATCATTTARCGTTTGKATTTGACCTCAGCTCAGNCGYGATTACCCGCTGAATTC

>ant-Nnud-6.394.1_NC5

TNNNTNTGCGCGTAATCGTGAGCCATGCAGCAAGCCATACACATGTGGTGGCAGCCGTCAGCTGTTTTGTGGCAGACAATGGCTTTGGCTTGCTGTGTGTGTTGAGGGGAAGTGAGTGACCCGATATGCTTCAAAGGCGGGTCGATTGCGCTCATTTTCTCGTTATTCGTAAAAACGGTATCCACTTTGGCGTCTACGCCGTACCTAGCTACTGCCTGGACCGTCGGTAGCGATGAAAGGTGCGGAGAAAGTTCCTCATTTTGAGTTGAGCAGACTTAATGAGCCCGGCAAGAGGGCCGCCAAAACCAAAACACAACCATTTCTTTATTTTTAATAGTTGATCGGTGAATCGGGGTGTTCGTTATGTGGCAACATGTGGTTGTCGCTGCGGATGTCTTGGTGAATCGTTAACTACAAATCTTGGCGGTGGATCACTCGGTTCGTGGATCGATGAAGAACGCAGCCAGCTGCGATAAATAGTGCGAATTGCAGACACATTGAGCACTAAAATTTCGAACGCACATTGCGCCATCGGGTTCATTCCCGCTGGCACGTCTGGCTGAGGGTCGAAATGTTCGACACTATCCGCACAATACTTCCTGTGAGCAGGGAAGTGTGTGGTGCATTCGGCAAGCAGTGATTCTTGAGCATTGCTCTCGAAAGCGCTCCTTGCTTAGACGGAACGGTAAATAATTGCTAAATTTTACTCGGTAAGGTGCAATTATAGCCGCGAATATGTTGCTTCTCGATGCGGCTTCCAGTATTTGTTGATTGYCGATAGCGACTGKAAGTATGGCATCKSATGCCTGATAMAK

>ant-Nnud-8.401.1_NC5

YATACACGCGTGGTGGCAGCCGTCTGCTGTGCTTTATCGTGCAGACAATGGCTTATGAGTGGCTGTGTGATTGTTGAACAACGGTGACCAATTTGGCGTCTACGCCGTATCTAGCTTCCGCCTGGACCGTCGGTAGCGATGAAAGATGCGGAGGAAGTTCCTCTGTTTTGGTTTCAACGCTAACGCAGAGTTGAGCAGACTTAATGAGCCACGCTTGGTGGCCGCCAAAACCCAAAACACAACCAGTCTATTTTAACGTTTGTTGATATGTTAATGTACAAATCTTGGCGGTGGATCACTCGGTTCGTGGATCGATGAAGAACGCAGCCAGCTGCGATAAATAGTGCGAATTGCAGACACATTGAGCACTAAGAATTCGAACGCACATTGCGCTATCGGGTTCATTCCCGATGGCACGTCTGGCTGAGGGTCGAATTGTAGTAAACTGTCTTCAAGTACTTTTTATGGTCGTGAAGTATTCGGCAAGCAGTTGTCGGATAGTTGTTTTTGTTGATCGTCCGTTCGTTCGGTCGGTCCAGAGCAATATTCTGAGGCTCCTTGCTTAGTTGCGTTTTGGTAGACGTTAACACCCGAACAATATGTGGTGGTGATATTGGTGATGGCGAGAATCATGCCGCTTCAATGGGGCAGCAACCAGCATACGCTAATGACAGTTGGTTGATTGAAGCCAGGGCAACGGAGTGATGTGTGGCGATCATTTAACGTTTGTATTTGACCTCAGCTCAGTCGTGATTACCCGCTGAATTTAAGCATATAATTAAGCGGAGGAAAANAAAAC

>ant-Nnud-9.408.1_93

TTTACTGGACGAGCGCATCTATTAGGTTAAAACCAATCGGGCTTTGCTCGTCAATTGGTGACTCTGAATAGCTATGGCTGATCGCATGGTCTTGTACCGGCGACATGTCTATCAAGTGTCTGCCTTATCAACTTTCGATGGTAGTTTATGTGCCTACCATGGTTGTAACGGGTAACGGAGAATAARGGTTCGACTCCGGAGAGGGAGCCTGAGAAACGGCTACCACATCCAAGGAAGGCRGCARGCGCGCAAATTACCCACTCTCGGCATGAGGAGGTAGTGACGAAAAATAACGAGACCGTTCTCTTCGAGGCCGGTTATCGGAATGGGTACAATTTAAACCCGTTAACGAGGATCTATGAGAGGGCAAGTCTGGTGCCAGCAGCCGCGGTAATTCCAGCTCTCAAAGTGTATATCGKCATTGCTGCGGKTAAAAAGCTCGTAGTTGGATCTGCGCCTCAGGACCTGGTCCGTCTTTTGGACGAGAACTGGGATCCTAGGCKAGTACTGTCGGTTTTCCCTGCGTTGCCTTCATCGGTCGCGCAGGGTGGCCGGYGAGTTTACCTTGAAAAAATTAGAGTGCTCAACGCGGTCTGACGCCTGAATACTCGTGCATGGAATAATGARATAGGATCTCGGTTCTATTTTGTTGGTTTTCTGATCTGAGATAATGGTTAAGAGGGACGKACGGKTAAGAGGGACGKACG

>ant-Lsqu-1.1.1_NC5

NGTCTCCCAACGTGCATACCATCCATTTGCATGTTGTTGTGAGCCGCATAGAAACTCATACACGCGTGGTGGCAGCCGTCTGCTGTGCTTTATCGTGCAGACAATGGCTTATGAGTGGCTGTGTGATTGTTGAACAACGGTGACCAATTTGGCGTCTACGCCGTATCTAGCTTCCGCCTGGACCGTCGGTAGCGATGAAAGATGCGGAGGAAGTTCCTCTGTTTTGGTTTCAACGCTAACGCAGAGTTGAGCAGACTTAATGAGCCACGCTTGGTGGCCGCCAAAACCCAAAACACAACCAGTCTATTTTAACGTTTGTTAATATGTTAATGTACAAATCTTGGCGGTGGATCACTCGGTTCGTGGATCGATGAAGAACGCAGCCAGCTGCGATAAATAGTGCGAATTGCAGACACATTGAGCACTAAGAATTCGAACGCACATTGCGCTATCGGGTTCATTCCCGATGRCACGTCTGGCTGAGGRTCGAATTGTAGTAAACTGTCTTCAAGTACTTTTTATGRTCGTGAAGTATTCGGCAAGCAGTTGTCGGATAGTTGTTTTTGTTGANNGN

>ant-Lsqu-14.131.1_NC5

GNGCCATGCAGCAAGCCATACACATGTGGTGGCAGCCGTCAGCTGTTTTGTGGCAGACAATGGCTTTGGCTTGCTGTGTGTGTTGAGGGGAAGTGAGTGACCCGATATGCTTCAAAGGCGGGTCGATTGCGCTCATTTTCTCGTTATTCGTAAAAACGGTATCCACTTTGGCGTCTACGCCGTACCTAGCTACTGCCTGGACCGTCGGTAGCGATGAAAGGTGCGGAGAAAGTTCCTCATTTTGAGTTGAGCAGACTTAATGAGCCCGGCAAGAGGGCCGCCAAAACCAAAACACAACCATTTCTTTATTTTTAATAGTTGATCGGTGAATCGGGGTGTTCGTTATGTGGCAACATGTGGTTGTCGCTGCGGATGTCTTGGTGAATCGTTAACTACAAATCTTGGCGGTGGATCACTCGGTTCGTGGATCGATGAAGAACGCAGCCAGCTGCGATAAATAGTGCGAATTGCAGACACATTGAGCACTAAAATTTCGAACGCACATTGCGCCATCGGGTTCATTCCCGCTGGCACGTCTGGCTGAGGGTCGAAATGTTCGACACTATCCGCACAATACTTCCTGTGAGCAGGGAAGTGTGTGGTGCATTCGGCAAGCAGTGATTCTTGAGCATTGCTCTCGAAAGCGCTCCTTGCTTAGACGGAACGGTAAATAATTGCTAAATTTTACTCGGTAAGGTGCAATTATAGCCGCGAATATGTTGCTTCTCGATGCGGCTTCCAGTATTTGTTGATTGTCGATAGCGACTGGAAGTATGGCATCGGATGCCTGATAGAGTGATATGTCTTGSYTGTTATG

>ant-Lsqu-15.143.1_NC5

ATACCNTCCATTTGCATGTTGTTGTGAGCCGCATAGAAACTCATACACGCGTGGTGGCAGCCGTCTGCTGTGCTTTATCGTGCAGACAATGGCTTATGAGTGGCTGTGTGATTGTTGAACAACGGTGACCAATTTGGCGTCTACGCCGTATCTAGCTTCCGCCTGGACCGTCGGTAGCGATGAAAGATGCGGAGGAAGTTCCTCTGTTTTGGTTTCAACGCTAACGCAGAGTTGAGCAGACTTAATGAGCCACGCTTGGTGGCCGCCAAAACCCAAAACACAACCAGTCTATTTTAACGTTTGTTGATATGTTAATGTACAAATCTTGGCGGTGGATCACTCGGTTCGTGGATCGATGAAGAACGCAGCCAGCTGCGATAAATAGTGCGAATTGCAGACACATTGAGCACTAAGAATTCGAACGCACATTGCGCTATCGGGTTCATTCCCGATGGCACGTCTGGCTGAGGGTCGAATTGTAGTAAACTGTCTTCAAGTACTTTTTATGGTCGTGAAGTATTCGGCAAGCAGTTGTCGGATAGTTGTTTTTGTTGATCGTCCGTTCGTTCGGTCGGTCCAGAGCAATATTCTGAGGCTCCTTGCTTAGTTGCGTTTTGGTAGACGTTAACACCCGAACAATATGTGGTGGTGATATTGGTGATGGCGAGAATCATGCCGCTTCAATGGGGCAGCAACCAGCATACGCTAATGACAGTTGGTTGATTGAAGCCAGGRCAACGGAGTGATGTGTGGCGATCATTTAACGTTTGTATTTGACCTCAGCTCAGTCGTGATTACCCGCTGAATTTAAGCATATAATTAAGCGYAGSAAA

>ant-Lsqu-18.162.1_NC5

TGCATACCATCCATTTGCATGTTGTTGTGAGCCGCATAGAAACTCATACACGCGTGGTGGCAGCCGTCTGCTGTGCTTTATCGTGCAGACAATGGCTTATGAGTGGCTGTGTGATTGTTGAACAACGGTGACCAATTTGGCGTCTACGCCGTATCTAGCTTCCGCCTGGACCGTCGGTAGCGATGAAAGATGCGGAGGAAGTTCCTCTGTTTTGGTTTCAACGCTAACGCAGAGTTGAGCAGACTTAATGAGCCACGCTTGGTGGCCGCCAAAACCCAAAACACAACCAGTCTATTTTAACGTTTGTTGATATGTTAATGTACAAATCTTGGCGGTGGATCACTCGGTTCGTGGATCGATGAAGAACGCAGCCAGCTGCGATAAATAGTGCGAATTGCAGACACATTGAGCACTAAGAATTCGAACGCACATTGCGCTATCGGGTTCATTCCCGATGGCACGTCTGGCTGAGGGTCGAATTGTAGTAAACTGTCTTCAAGTACTTTTTATGGTCGTGAAGTATTCGGCAAGCAGTTGTCGGATAGTTGTTTTTGTTGATCGTCCGTTCGTTCGGTCGGTCCAGAGCAATATTCTGAGGCTCCTTGCTTAGTTGCGTTTTGGTAGACGTTAACACCCGAACAATATGTGGTGGTGATATTGKTGATGN

>ant-Lsqu-20.187.1_NC5

GGCAKACAANGGSTTTGGCTTGCTGTGTGTGTTGAGGGGAAGTGAGTGACCCGATATGCTTCAAAGGCGGGTCGATTGCGCTCATTTTCTCGTTATTCGTAAAAACGGTATCCACTTTGGCGTCTACGCCGTACCTAGCTACTGCCTGGACCGTCGGTAGCGATGAAAGGTGCGGAGAAAGTTCCTCATTTTGAGTTGAGCAGACTTAATGAGCCCGGCAAGAGGGCCGCCAAAACCAAAACACAACCATTTCTTTATTTTTAATAGTTGATCGGTGAATCGGGGTGTTCGTTATGTGGCAACATGTGGTTGTCGCTGCGGATGTCTTGGTGAATCGTTAACTACAAATCTTGGCGGTGGATCACTCGGTTCGTGGATCGATGAAGAACGCAGCCAGCTGCGATAAATAGTGCGAATTGCAGACACATTGAGCACTAAAATTTCGAACGCACATTGCGCCATCGGGTTCATTCCCGCTGGCACGTCTGGCTGAGGGTCGAAATGTTCGACACTATCCGCACAATACTTCCTGTGAGCAGGGAAGTGTGTGGTGCATTCGGCAAGCAGTGATTCTTGAGCATTGCTCTCGAAAGCGCTCCTTGCTTAGACGGAACGGTAAATAATTGCTAAATTTTACTCGGTAAGGTGCAATTATAGCCGCGAATATGTTGCTTCTCGATGCGGCTTCCAGTATTTGTTGATTGTCGATAGCGACTGGAAGTATGGCATCGGATGCCTGATAGAGTGATATGTCTTGGTTGTTATGTGTTTTTGACCTCAGCTCAGTCGTGATTACCCGCTGAATTTAAGCATATAATTAAGCGGAGGAAAARRAAACTAAAA

>ant-Lsqu-25.231.1_NC5

NCGTGCATAAATTCCATTTGCGCGTAATCGTGAGCCATGCAGCAAGCCATACACATGTGGTGGCAGCCGTCAGCTGTTTTGTGGCAGACAATGGCTTTGGCTTGCTGTGTGTGTTGAGGGGAAGTGAGTGACCCGATATGCTTCAAAGGCGGGTCGATTGCGCTCATTTTCTCGTTATTCGTAAAAACGGTATCCACTTTGGCGTCTACGCCGTACCTAGCTACTGCCTGGACCGTCGGTAGCGATGAAAGGTGCGGAGAAAGTTCCTCATTTTGAGTTGAGCAGACTTAATGAGCCCGGCAAGAGGGCCGCCAAAACCAAAACACAACCATTTCTTTATTTTTAATAGTTGATCGGTGAATCGGGGTGTTCGTTATGTGGCAACATGTGGTTGTCGCTGCGGATGTCTTGGTGAATCGTTAACTACAAATCTTGGCGGTGGATCACTCGGTTCGTGGATCGATGAAGAACGCAGCCAGCTGCGATAAATAGTGCGAATTGCAGACACATTGAGCACTAAAATTTCGAACGCACATTGCGCCATCGGGTTCATTCCCGCTGGCACGTCTGGCTGAGGGTCGAAATGTTCGACACTATCCGCACAATACTTCCTGTGAGCAGGGAAGTGTGTGGTGCATTCGGCAAGCAGTGATTCTTGAGCATTGCTCTCGAAAGCGCTCCTTGCTTAGACGGAACGGTAAATAATTGCTAAATTTTACTCGGTAAGGTGCAATTATAGCCGCGAATATGTTGCTTCTCGATGCGGCTTCCAGTATTTGTTGATTGTCGATAGCGACTGGAAGTATGGCATCGGATGCCTGATAGAGTGATATGTCTTGTYTGYTATG

>ant-Lsqu-26.235.1_NC5

CGTGCATAAATTCCATTTGCGCGTAATCGTGAGCCATGCAGCAAGCCATACACATGTGGTGGCAGCCGTCAGCTGTTTTGTGGCAGACAATGGCTTTGGCTTGCTGTGTGTGTTGAGGGGAAGTGAGTGACCCGATATGCTTCAAAGGCGGGTCGATTGCGCTCATTTTCTCGTTATTCGTAAAAACGGTATCCACTTTGGCGTCTACGCCGTACCTAGCTACTGCCTGGACCGTCGGTAGCGATGAAAGGTGCGGAGAAAGTTCCTCATTTTGAGTTGAGCAGACTTAATGAGCCCGGCAAGAGGGCCGCCAAAACCAAAACACAACCATTTCTTTATTTTTAATAGTTGATCGGTGAATCGGGGTGTTCGT

>ant-Lsqu.28.245.1_NC5

NTCTCCCANCGTGCATACCATCCATTTGCATGTTGTTGTGAGCCGCATAGAAACTCATACACGCGTGGTGGCAGCCGTCTGCTGTGCTTTATCGTGCAGACAATGGCTTATGAGTGGCTGTGTGATTGTTGAACAACGGTGACCAATTTGGCGTCTACGCCGTATCTAGCTTCCGCCTGGACCGTCGGTAGCGATGAAAGATGCGGAGGAAGTTCCTCTGTTTTGGTTTCAACGCTAACGCAGAGTTGAGCAGACTTAATGAGCCACGCTTGGTGGCCGCCAAAACCCAAAACACAACCAGTCTATTTTAACGTTTGTTGATATGTTAATGTACAAATCTTGGCGGTGGATCACTCGGTTCGTGGATCGATGAAGAACGCAGCCAGCTGCGATAAATAGTGCGAATTGCAGACACATTGAGCACTAAGAATTCGAACGCACATTGCGCTATCGGGTTCATTCCCGATGGCACGTCTGGCTGAGGGTCGAATTGTAGTAAACTGTCTTCAAGTACTTTTTATGGTCGTGAAGTATTCGGCAAGCAGTTGTCGGATAGTTGTTTTTGTTGATCGTCCGTTCGTTCGGTCGGTCCAGAGCAATATTCTGAGGCTCCTTGCTTAGTTGCGTTTTGGTAGACGTTAACACCCGAACAATATGTGGTGGTGATATTGGTGATGGCGAGAATCATGCCGCTTCAATGGGGCAGCAACCAGCATACGCTAATGACAGTTGGTTGATTGAAGCCAGGGCAACGGAGTGATGTGTGGCGATCATTTAACGTTTGTATTTGACCTCAGCTCAGTCGTGATTACCCGCTGAATTTAAGCATATAATTAAGCGGAGGAAANNRAAACTAAAA

>ant-Lsqu-31.266.1_NC5

CCANCGTGCATACCATCCATTTGCATGTTGTTGTGAGCCGCATAGAAACTCATACACGCGTGGTGGCAGCCGTCTGCTGTGCTTTATCGTGCAGACAATGGCTTATGAGTGGCTGTGTGATTGTTGAACAACGGTGACCAATTTGGCGTCTACGCCGTATCTAGCTTCCGCCTGGACCGTCGGTAGCGATGAAAGATGCGGAGGAAGTTCCTCTGTTTTGGTTTCAACGCTAACGCAGAGTTGAGCAGACTTAATGAGCCACGCTTGGTGGCCGCCAAAACCCAAAACACAACCAGTCTATTTTAACGTTTGTTGATATGTTAATGTACAAATCTTGGCGGTGGATCACTCGGTTCGTGGATCGATGAAGAACGCAGCCAGCTGCGATAAATAGTGCGAATTGCAGACACATTGAGCACTAAGAATTCGAACGCACATTGCGCTATCGGGTTCATTCCCGATGGCACGTCTGGCTGAGGGTCGAATTGTAGTAAACTGTCTTCAAGTACTTTTTATGGTCGTGAAGTATTCGGCAAGCAGTTGTCGGATAGTTGTTTTTGTTGATCGTCCGTTCGTTCGGTCGGTCCAGAGCAATATTCTGAGGCTCCTTGCTTAGTTGCGTTTTGGTAGACGTTAACACCCGAACAATATGTGGTGGTGATATTGGTGATGGCGAGAATCATGCCGCTTCAATGGGGCAGCAACCAGCATACGCTAATGACAGTTGGTTGATTGAAGCCAGGGCAACGGAGTGATGTGTGGCGATCATTTAACGTTTGTATTTGACCTCAGCTCAGTCGTGATTACCCGCTGAATTTAAGCATATAATTAAGCGGAGGAAAAGAAAACTAAAN

>ant-Lsqu-35.281.1_NC5

ANNNCTCCCACGTGCATACCATCCATTTGCATGTTGTTGTGAGCCGCATAGAAACTCATACACGCGTGGTGGCAGCCGTCTGCTGTGCTTTATCGTGCAGACAATGGCTTATGAGTGGCTGTGTGATTGTTGAACAACGGTGACCAATTTGGCGTCTACGCCGTATCTAGCTTCCGCCTGGACCGTCGGTAGCGATGAAAGATGCGGAGGAAGTTCCTCTGTTTTGGTTTCAACGCTAACGCAGAGTTGAGCAGACTTAATGAGCCACGCTTGGTGGCCGCCAAAACCCAAAACACAACCAGTCTATTTTAACGTTTGTTGATATGTTAATGTACAAATCTTGGCGGTGGATCACTCGGTTCGTGGATCGATGAAGAACGCAGCCAGCTGCGATAAATAGTGCGAATTGCAGACACATTGAGCACTAAGAATTCGAACGCACATTGCGCTATCGGGTTCATTCCCGATGGCACGTCTGGCTGAGGGTCGAATTGTAGTAAACTGTCTTCAAGTACTTTTTATGGTCGTGAAGTATTCGGCAAGCAGTTGTCGGATAGTTGTTTTTGTTGATCGTCCGTTCGTTCGGTCGGTCCAGAGCAATATTCTGAGGCTCCTTGCTTAGTTGCGTTTTGGTAGACGTTAACACCCGAACAATATGTGGTGGTGATATTGGTGATGGCGAGAATCATGCCGCTTCAATGGGGCAGCAACCAGCATACGCTAATGACAGTTGGTTGATTGAAGCCAGGGCAACGGAGTGATGTGTGGCGATCATTTAACGTTTGTATTTGACCTCAGCTCAGTCGTGATTACCCGCTGAATTTAAGCATATAATAAGCGAGAAAAAAAAAAA

>ant-Lsqu-37.287.1_NC5

ATACCATCCATTTGCATGTTGTTGTGAGCCGCATAGAAACTCATACACGCGTGGTGGCAGCCGTCTGCTGTGCTTTATCGTGCAGACAATGGCTTATGAGTGGCTGTGTGATTGTTGAACAACGGTGACCAATTTGGCGTCTACGCCGTATCTAGCTTCCGCCTGGACCGTCGGTAGCGATGAAAGATGCGGAGGAAGTTCCTCTGTTTTGGTTTCAACGCTAACGCAGAGTTGAGCAGACTTAATGAGCCACGCTTGGTGGCCGCCAAAACCCAAAACACAACCNGTCTATTTTAACGTTTGTTGATATGTTAATGTA

>ant-Lsqu-39.302.1_NC5

NNTNTGCGCGTAATCGTGAGCCATGCAGCAAGCCATACACATGTGGTGGCAGCCGTCAGCTGTTTCGTGGCAGACAATGGCTTTGGCTTGCTGTGTGTGTTGAGGGGAAGTGAGTGACCCGATATGCTTCAAAGGCGGGTCGATTGCGCTCATTTTCTCGTTATTCGTAAAAACGGTATCCACTTTGGCGTCTACGCCGTACCTAGCTACTGCCTGGACCGTCGGTAGCGATGAAAGGTGCGGAGAAAGTTCCTCATTTTGAGTTGAGCAGACTTAATGAGCCCGGCAAGAGGGCCGCCAAAACCAAAACACAACCATTTCTTTATTTTTAATAGTTGATCGGTGAATCGGGGTGTTCGTTATGTGGCAACATGTGGTTGTCGCTGCGGATGTCTTGGTGAATCGTTAACTACAAATCTTGGCGGTGGATCACTCGGTTCGTGGATCGATGAAGAACGCAGCCAGCTGCGATAAATAGTGCGAATTGCAGACACATTGAGCACTAAAATTTCGAACGCACATTGCGCCATCGGGTTCATTCCCGCTGGCACGTCTGGCTGAGGGTCGAAATGTTCGACACTATCCGCACAATACTTCCTGTGAGCAGGGAAGTGTGTGGTGCATTCGGCAAGCAGTGATTCTTGAGCATTGCTCTCGAAAGCGCTCCTTGCTTAGACGGAACGGTAAATAATTGCTAAATTTTACTCGGTAAGGTGCAATTATAGCCGCGAATATGTTGCTTCTCGATGCGGCTTCCAGTATTTGTTGATTGTCGATAGCGACTGGAAGTATGGCATCGGATGCCTGATAGAGTGATATGTCTTGGTTGTTATGTGTTTTTGACCTCAGCTCAGTCGTGATTACCCGCTGAATTTAAGCATATAATTAAGCGGAGGAAAARMAAACTAA

>ant-Lsqu-4.53.1_NC5

TCTCCCAACGTGCATACCATCCATTTGCATGTTGTTGTGAGCCGCATAGAAACTCATACACGCGTGGTGGCAGCCGTCTGCTGTGCTTTATCGTGCAGACAATGGCTTATGAGTGGCTGTGTGATTGTTGAACAACGGTGACCAATTTGGCGTCTACGCCGTATCTAGCTTCCGCCTGGACCGTCGGTAGCGATGAAAGATGCGGAGGAAGTTCCTCTGTTTTGGTTTCAACGCTAACGCAGAGTTGAGCAGACTTAATGAGCCACGCTTGGTGGCCGCCAAAACCCAAAACACAACCAGTCTATTTTAACGTTTGTTGATATGTTAATGTACAAATCTTGGCGGTGGATCACTCGGTTCGTGGATCGATGAAGAACGCAGCCAGCTGCGATAAATAGTGCGAATTGCAGACACATTGAGCACTAAGAATTCGAACGCACATTGCGCTATCGGGTTCATTCCCGATGGCACGTCTGGCTGAGGGTCGAATTGTAGTAAACTGTCTTCAAGTACTTTTTATGGTCGTGAAGTATTCGGCAAGCAGTTGTCGGATAGTTGTTTTTGTTGATCGTCCGTTCGTTCGGTCGGTCCAGAGCAATATTCTGAGGCTCCTTGCTTAGTTGCGTTTTGGTAGACGTTAACACCCGAACAATATGTGGTGGTGATATTTGGTGATGNCGAGAATCATGCCGCTTCAATGGGGCAGCAACCAGCATACGCTAATGACAGTTGGTTGATTGAAGCCAGGGCAACGGAGTGATGTGTGGCGATCATTTAACGTTTGTATTTGACCTCAGCTCAGTCGTGATTACCCGCTGAATTTAAGCATATAATTAAGCGGAGGAAARMA

>ant-Lsqu-40.316.1_NC5

NTCTCCCANCGTGCATACCATCCATTTGCATGTTGTTGTGAGCCGCATAGAAACTCATACACGCGTGGTGGCAGCCGTCTGCTGTGCTTTATCGTGCAGACAATGGCTTATGAGTGGCTGTGTGATTGTTGAACAACGGTGACCAATTTGGCGTCTACGCCGTATCTAGCTTCCGCCTGGACCGTCGGTAGCGATGAAAGATGCGGAGGAAGTTCCTCTGTTTTGGTTTCAACGCTAACGCAGAGTTGAGCAGACTTAATGAGCCACGCTTGGTGGCCGCCAAAACCCAAAACACAACCAGTCTATTTTAACGTTTGTTGATATGTTAATGTACAAATCTTGGCGGTGGATCACTCGGTTCGTGGATCGATGAAGAACGCAGCCAGCTGCGATAAATAGTGCGAATTGCAGACACATTGAGCACTAAGAATTCGAACGCACATTGCGCTATCGGGTTCATTCCCGATGGCACGTCTGGCTGAGGGTCGAATTGTAGTAAACTGTCTTCAAGTACTTTTTATGGTCGTGAAGTATTCGGCAAGCAGTTGTCGGATAGTTGTTTTTGTTGATCGTCCGTTCGTTCGGTCGGTCCAGAGCAATATTCTGAGGCTCCTTGCTTAGTTGCGTTTTGGTAGACGTTAACACCCGAACAATATGTGGTGGTGATATTGGTGATGGCGAGAATCATGCCGCTTCAATGGGGCAGCAACCAGCATACGCTAATGACAGTTGGTTGATTGAAGCCAGGGCAACGGAGTGATGTGTGGCGATCATTTAACGTTTGTATTTGACCTCAGCTCAGTCGTGATTACCCGCTGAATTTAAGCATATAATTAAGCGGAGAAAANNAAAAA

>ant-Lsqu-41.315.1_NC5

ACGTGCATAAATTCCATTTGCGCGTAATCGTGAGCCATGCAGCAAGCCATACACATGTGGTGGCAGCCGTCAGCTGTTTTGTGGCAGACAATGGCTTTGGCTTGCTGTGTGTGTTGAGGGGAAGTGAGTGACCCGATATGCTTCAAAGGCGGGTCGATTGCGCTCATTTTCTCGTTATTCGTAAAAACGGTATCCACTTTGGCGTCTACGCCGTACCTAGCTACTGCCTGGACCGTCGGTAGCGATGAAAGGTGCGGAGAAAGTTCCTCATTTTGAGTTGAGCAGACTTAATGAGCCCGGCAAGAGGGCCGCCAAAACCAAAACACAACCATTTCTTTATTTTTAATAGTTGATCGGTGAATCGGGGTGTTCGTTATGTGGCAACATGTGGTTGTCGCTGCGGATGTCTTGGTGAATCGTTAACTACAAATCTTGGCGGTGGATCACTCGGTTCGTGGATCGATGAAGAACGCAGCCAGCTGCGATAAATAGTGCGAATTGCAGACACATTGAGCACTAAAATTTCGAACGCACATTGCGCCATCGGGTTCATTCCCGCTGGCACGTCTGGCTGAGGGTCGAAATGTTCGACACTATCCGCACAATACTTCCTGTGAGCAGGGAAGTGTGTGGTGCATTCGGCAAGCAGTGATTCTTGAGCATTGCTCTCGAAAGCGCTCCTTGCTTAGACGGAACGGTAAATAATTGCTAAATTTTACTCGGTAAGGTGCAATTATAGCCGCGAATATGTTGCTTCTCGATGCGGCTTCCAGTATTTGTTGATTGTCGATAGCGACTGGAAGTATGGCATCGGATGCCTGATAGAGTGATATGTCTTGGTTGTTATGTGTTTTTGACCTCAGCTCAGTCGTGATTACCCGCTGAATTTAAGCATATAATTAACCGAGA

>ant-Lsqu-42.321.1_NC5

NNCGTGCATACCATCCATTTGCATGTTGTTGTGAGCCGCATAGAAACTCATACACGCGTGGTGGCAGCCGTCTGCTGTGCTTTATCGTGCAGACAATGGCTTATGAGTGGCTGTGTGATTGTTGAACAACGGTGACCAATTTGGCGTCTACGCCGTATCTAGCTTCCGCCTGGACCGTCGGTAGCGATGAAAGATGCGGAGGAAGTTCCTCTGTTTTGGTTTCAACGCTAACGCAGAGTTGAGCAGACTTAATGAGCCACGCTTGGTGGCCGCCAAAACCCAAAACACAACCAGTCTATTTTAACGTTTGTTGATATGTTAATGTACAAATCTTGGCGGTGGATCACTCGGTTCGTGGATCGATGAAGAACGCAGCCAGCTGCGATAAATAGTGCGAATTGCAGACACATTGAGCACTAAGAATTCGAACGCACATTGCGCTATCGGGTTCATTCCCGATGGCACGTCTGGCTGAGGGTCGAATTGTAGTAAACTGTCTTCAAGTACTTTTTATGGTCGTGAAGTATTCGGCAAGCAGTTGTCGGATAGTTGTTTTTGTTGATCGTCCGTTCGTTCGGTCGGTCCAGAGCAATATTCTGAGGCTCCTTGCTTAGTTGCGTTTTGGTAGACGTTAACACCCGAACAATATGTGGTGGTGATATTTGGTGATGNGCGAGAATCATGCCGCTTCAATGGGGCAGCAACCAGCATACGCTAATGACAGTTGGTTGATTGAAGCCAGGGCAACGGAGTGATGTGTGGCGATCATTTAACGTTTGTATTTGACCTCAGCTCAGTCGTGATTACCCGCTGAATTTAAGCATATAATTAAGCGAGAAANNNAAAA

>ant-Lsqu-43.332.1_NC5

CTCCCAACGTGCATACCATCCATTTGCATGTTGTTGTGAGCCGCATAGAAACTCATACACGCGTGGTGGCAGCCGTCTGCTGTGCTTTATCGTGCAGACAATGGCTTATGAGTGGCTGTGTGATTGTTGAACAACGGTGACCAATTTGGCGTCTACGCCGTATCTAGCTTCCGCCTGGACCGTCGGTAGCGATGAAAGATGCGGAGGAAGTTCCTCTGTTTTGGTTTCAACGCTAACGCAGAGTTGAGCAGACTTAATGAGCCACGCTTGGTGGCCGCCAAAACCCAAAACACAACCAGTCTATTTTAACGTTTGTTGATATGTTAATGTACAAATCTTGGCGGTGGATCACTCGGTTCGTGGATCGATGAAGAACGCAGCCAGCTGCGATAAATAGTGCGAATTGCAGACACATTGAGCACTAAGAATTCGAACGCACATTGCGCTATCGGGTTCATTCCCGATGGCACGTCTGGCTGAGGGTCGAATTGTAGTAAACTGTCTTCAAGTACTTTTTATGGTCGTGAAGTATTCGGCAAGCAGTTGTCGGATAGTTGTTTTTGTTGATCGTCCGTTCGTTCGGTCGGTCCAGAGCAATATTCTGAGGCTCCTTGCTTAGTTGCGTTTTGGTAGACGTTAACACCCGAACAATATGTGGTGGTGATATTTGGTGATGGCGAGAATCATGCCGCTTCAATGGGGCAGCAACCAGCATACGCTAATGACAGTTGGTTGATTGAAGCCAGGNCAACGGAGTGATGTGTGGCGATCATTTAACGTTTGTATTTGACCTCAGCTCAGTCGTGATTACCCGCTGAATTTAAGCATATAATTAAGCGGNGNAA

>ant-Lsqu-44.340.1_NC5

GTGCATAAATTCCATTTGCGCGTAATCGTGAGCCATGCAGCAAGCCATACACATGTGGTGGCAGCCGTCAGCTGTTTTGTGGCAGACAATGGCTTTGGCTTGCTGTGTGTGTTGAGGGGAAGTGAGTGACCCGATATGCTTCAAAGGCGGGTCGATTGCGCTCATTTTCTCGTTATTCGTAAAAACGGTATCCACTTTGGCGTCTACGCCGTACCTAGCTACTGCCTGGACCGTCGGTAGCGATGAAAGGTGCGGAGAAAGTTCCTCATTTTGAGTTGAGCAGACTTAATGAGCCCGGCAAGAGGGCCGCCAAAACCAAAACACAACCATTTCTTTATTTTTAATAGTTGATCGGTGAATCGGGGTGTTCGTTATGTGGCAACATGTGGTTGTCGCTGCGGATGTCTTGGTGAATCGTTAACTACAAATCTTGGCGGTGGATCACTCGGTTCGTGGATCGATGAAGAACGCAGCCAGCTGCGATAAATAGTGCGAATTGCAGACACATTGAGCACTAAAATTTCGAACGCACATTGCGCCATCGGGTTCATTCCCGCTGGCACGTCTGGCTGAGGGTCGAAATGTTCGACACTATCCGCACAATACTTCCTGTGAGCAGGGAAGTGTGTGGTGCATTCGGCAAGCAGTGATTCTTGAGCATTGCTCTCGAAAGCGCTCCTTGCTTAGACGGAACGGTAAATAATTGCTAAATTTTACTCGGTAAGGTGCAATTATAGCCGCGAATATGTTGCTTCTCGATGCGGCTTCCAGTATTTGTTGATTGTCGATAGCGACTGGAAGTATGGCATCGGATGCCTGATAGAGTGATATGTCTTGNTTGYTATGYGTTTT

>ant-Lsqu-49.364.1_NC5

TCTCCCAACGTGCATACCATCCATTTGCATGTTGTTGTGAGCCGCATAGAAACTCATACACGCGTGGTGGCAGCCGTCTGCTGTGCTTTATCGTGCAGACAATGGCTTATGAGTGGCTGTGTGATTGTTGAACAACGGTGACCAATTTGGCGTCTACGCCGTATCTAGCTTCCGCCTGGACCGTCGGTAGCGATGAAAGATGCGGAGGAAGTTCCTCTGTTTTGGTTTCAACGCTAACGCAGAGTTGAGCAGACTTAATGAGCCACGCTTGGTGGCCGCCAAAACCCAAAACACAACCAGTCTATTTTAACGTTTGTTAATATGTTAATGTACAAATCTTGGCGGTGGATCACTCGGTTCGTGGATCGATGAAGAACGCAGCCAGCTGCGATAAATAGTGCGAATTGCAGACACATTGAGCACTAAGAATTCGAACGCACATTGCGCTATCGGGTTCATTCCCGATGGCACGTCTGGCTGAGGGTCGAATTGTAGTAAACTGTCTTCAAGTACTTTTTATGGTCGTGAAGTATTCGGCAAGCAGTTGTCGGATAGTTGTTTTTGTTGATCGTCCGTTCGTTCGGTCGGTCCAGAGCAATATTCTGAGGCTCCTTGCTTAGTTGCGTTTTGGTAGACGTTAACACCCGAACAATATGTGGTGGTGATATTGGTGATGNCGAGAATCATGCCGCTTCAATGNNGNAGCNACCAGCATACGCTAATGACAGWTGGYTGAYTGMAGSCAGGAAWAMGTAYTGATGTG
